# Mechanistic insights into the role of FAT10 in modulating NCOA4-mediated ferroptosis in pancreatic acinar cells during acute pancreatitis

**DOI:** 10.1038/s41419-025-07715-9

**Published:** 2025-05-15

**Authors:** Lingpeng Liu, Ben Che, Wenming Zhang, Dongnian Du, Dandan Zhang, Jiajuan Li, Zehao Chen, Xuzhe Yu, Miao Ye, Wei Wang, Zijing Li, Fei Xie, Qing Wang, Leifeng Chen, Jianghua Shao

**Affiliations:** 1 Department of General Surgery, Second Affiliated Hospital of Nanchang University, Nanchang, China; 2 Jiangxi Province Key Laboratory of Molecular Medicine, Second Affiliated Hospital of Nanchang University, Nanchang, China; 3 Liver Cancer Institute, Nanchang University, Nanchang, China; 4 Jiangxi Province Clinical Research Center of General Surgery, Second Affiliated Hospital of Nanchang University, Nanchang, China; 5 Precision Oncology Medicine Center, Second Affiliated Hospital of Nanchang University, Nanchang, Jiangxi Province China; 6 The MOE Basic Research and Innovation Center for the Targeted Therapeutics of Solid Tumors, Nanchang University, Nanchang, China

**Keywords:** Ubiquitylation, Acute pancreatitis

## Abstract

Acute pancreatitis (AP) is characterised by inflammation and cell death in pancreatic tissue, with ferroptosis playing a critical role in its pathophysiology by mediating cellular damage and exacerbating inflammation. This study investigated the role of human leukocyte antigen (HLA)-F adjacent transcript 10 (FAT10) in AP, specifically its involvement in ferroptosis within pancreatic acinar cells. We observed that FAT10 expression was significantly elevated in AP tissues, which correlated with increased ferroptosis. Overexpression of FAT10 in pancreatic acinar cells enhances ferroptosis, whereas its knockdown reduced levels of ferroptosis markers. Furthermore, we confirmed that FAT10 enhanced ferroptosis in pancreatic acinar cells primarily by upregulating nuclear receptor coactivator 4 (NCOA4) expression. Mechanistic investigations revealed that FAT10 regulates NCOA4 expression to promote ferroptosis in a complex manner. FAT10 inhibits NCOA4 ubiquitination by reducing ubiquitin-NCOA4 complexes. Meanwhile, NCOA4 expression increased alongside the increase in FAT10-NCOA4 complexes, which are resistant to proteasomal degradation. Notably, we identified silibinin, a natural compound, as an effective inhibitor of the FAT10-NCOA4 axis, leading to reduced ferroptosis and alleviation of pancreatic damage in vivo. Silibinin treatment decreased the levels of ferroptosis-related proteins and inflammatory markers in both cell and animal models. Our findings highlight the FAT10-NCOA4 axis as a crucial regulator of ferroptosis in pancreatic acinar cells and suggest that targeting this pathway could offer a therapeutic strategy for mitigating AP. This study provides new insights into the regulatory mechanisms of ferroptosis in pancreatic acinar cells, identifying FAT10 as a potential therapeutic target for AP management.

## Introduction

Acute pancreatitis (AP) is a complex inflammatory condition characterised by impaired exocrine function, potentially leading to high morbidity and mortality [1]. It is the fifth most common cause of inpatient mortality worldwide, and its rising global incidence has become a major public health concern [2]. The pathophysiology of AP primarily involves the abnormal activation of zymogens in pancreatic acinar cells owing to various factors, resulting in the release of a numerous inflammatory mediators. This triggers an acute inflammatory response, which ultimately results in acinar cell death [3, 4]. Recent studies have highlighted the significant roles of various factors in mediating programmed cell death in pancreatic acinar cells during AP progression [5–7]. Therefore, investigating novel mediators of programmed cell death in pancreatic acinar cells is essential for comprehending AP pathogenesis and may provide new therapeutic avenues.

Ferroptosis is a programmed cell death process characterised by iron-dependent lipid peroxidation, resulting in elevated levels of reactive oxygen species (ROS) and subsequent cellular damage and death [8, 9]. In the context of AP, ferroptosis has emerged as a significant contributor to acinar cell injury and death. The inflammatory environment associated with AP may enhance iron accumulation and oxidative stress, promoting ferroptosis [10]. Studies have shown that the interplay between inflammation and iron metabolism can exacerbate pancreatic injury by increasing lipid peroxidation and cell death, further complicating disease progression. Additionally, the dysregulation of iron homeostasis in AP may lead to a self-perpetuating cycle of inflammation and cell death, underscoring the importance of ferroptosis in disease pathogenesis [3, 11]. Understanding the role of ferroptosis in AP is critical for elucidating the mechanisms underlying acinar cell death and inflammation. Targeting ferroptosis through modulation of iron levels or enhancement of antioxidant defences may offer new therapeutic strategies for managing AP.

Nuclear receptor coactivator 4 (NCOA4) is an important intracellular protein within the nuclear receptor coactivator family. It primarily functions as a coactivator for transcription factors by binding to nuclear receptors, enhancing their transcriptional activity, and thereby regulating gene expression [12]. Beyond transcriptional regulation, NCOA4 is involved in various biological processes, including cell growth, differentiation, metabolism, and particularly in ferroptosis [13–15]. NCOA4 functions as a selective cargo receptor that facilitates ferritinophagy. It forms a complex with ferritin heavy chain (FTH1) by binding to the surface residue R23 of FTH1 through the C-terminal amino acid residues I489 and W497 of NCOA4 [16]. This complex is then directed into the autophagolysosome, where ferritin is degraded through autophagy, releasing of Fe²⁺ ions. This release enhances the Fenton reaction, intensifying the peroxidation of polyunsaturated fatty acid-containing phospholipids and producing substantial amounts of reactive oxygen species (ROS), leading to ferroptosis in diverse cell types [8]. Research has reported that several factors regulate NCOA4 expression, thereby mediating ferroptosis in different cell types. For example, YTHDC2 reduces the m6A methylation of NCOA4, stabilising its expression, and promoting ferritinophagy, ultimately leading to ferroptosis in rat neuronal cells [17]. ATM kinase promotes the phosphorylation of NCOA4, enhancing ferritinophagy and consequently exacerbating ferroptosis in mouse fibroblasts [18]. Phosphorylated STAT3 upregulates NCOA4 protein expression, promoting ferroptosis in cardiomyocytes [19]. However, it remains unclear whether NCOA4 mediates ferroptosis in pancreatic acinar cells and which regulatory factors influence this process, necessitating further investigation.

Post-translational modifications (PTMs) are critical for regulating protein function, activity, and localisation. Among these modifications, ubiquitin-like proteins have emerged as key regulators [20]. Recent studies have highlighted the importance of ubiquitin-like proteins in modulating various forms of programmed cell death, particularly ferroptosis [21, 22]. These ubiquitin-like proteins influence the stability, interactions, and activity of key molecules in the ferroptosis pathway, thereby playing pivotal roles in the initiation and execution of ferroptosis [14]. Human leukocyte antigen (HLA)-F adjacent transcript 10 (FAT10), a member of the ubiquitin-like protein family, consists of two ubiquitin-like domains connected by a short linker and plays an important role in various diseases, including tumours, myocardial injury, and fatty liver disease [23–25]. Under hypoxia and inflammatory environments, the expression of FAT10 is significantly increased [26]. This upregulation is typically associated with cellular stress responses, indicating that FAT10 plays a key role in regulating responses to hypoxia and inflammation [27]. FAT10 is a unique ubiquitin-like protein that can directly mediate ubiquitin-independent proteasomal degradation of substrates [28–30]. Recently, our research confirmed that FAT10 stabilises the expression of substrate proteins, revealing a dual role for FAT10 within the cell [23, 31–33]. FAT10 can enhance the stability of specific substrates through direct interaction, thereby preserving their function. Notably, FAT10 is involved not only in immune-mediated inflammation, cell cycle regulation, and intracellular signalling but has also been reported to promote apoptosis [34–38]. Studies have indicated that FAT10 expression is elevated in human immunodeficiency virus-associated kidney disease, leading to apoptosis in renal tubular epithelial cells [39]. FAT10 promotes apoptosis in mouse fibroblasts via a caspase-dependent mechanism [40]. However, it is unclear whether FAT10 is involved in ferroptosis of pancreatic acinar cells during AP.

In this study, we explored the function of FAT10 in AP, focusing on its role in ferroptosis of pancreatic acinar cells. Using a rat model of AP and cell-based experiments, we confirmed that FAT10 plays a critical role in modulating ferroptosis in pancreatic acinar cells. Furthermore, we demonstrated that FAT10 promotes ferroptosis by upregulating the expression of NCOA4. Mechanistically, we revealed that FAT10 stabilises NCOA4 protein levels by antagonising its ubiquitination and forming a FAT10-NCOA4 complex resistant to proteasomal degradation. Additionally, our results showed that the FAT10 inhibitor silibinin suppresses ferroptosis by inhibiting the FAT10-NCOA4 axis, thereby alleviating inflammatory response and tissue damage. These findings provide new insights into the regulatory mechanisms underlying ferroptosis in pancreatic acinar cells, and suggest that FAT10 may serve as a potential therapeutic target for AP.

## Results

### FAT10 expression and ferroptosis levels are increased in a rat model of AP induced by cerulein

**Fig. 1:**
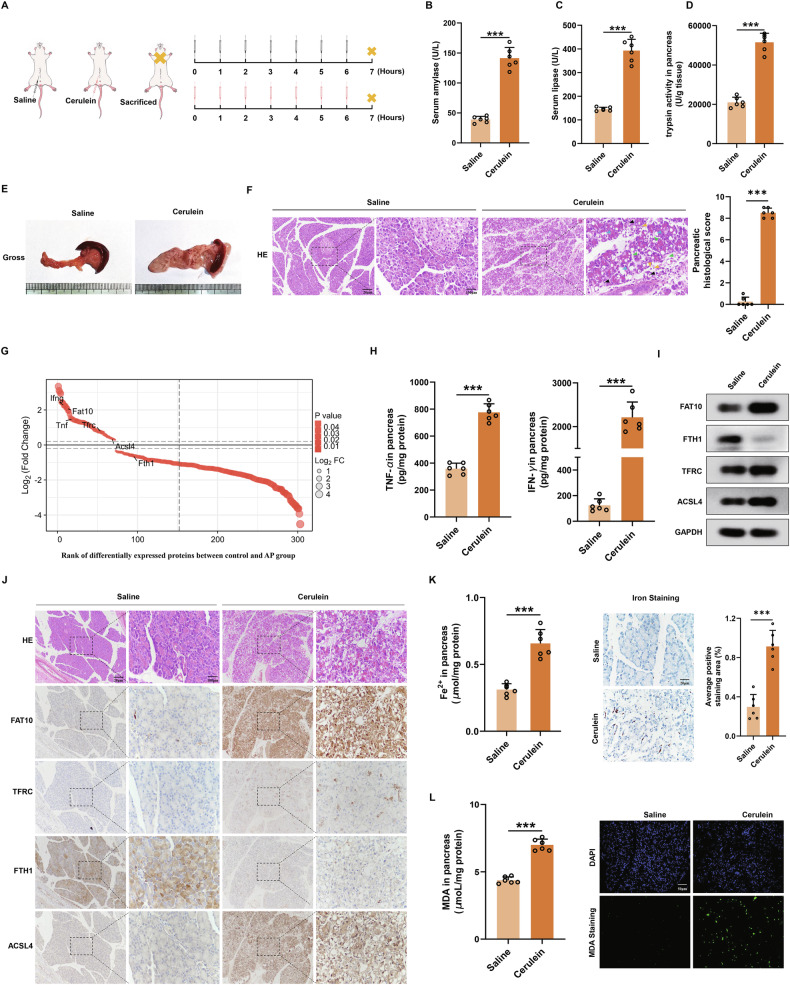
FAT10 expression and ferroptosis levels are increased in a rat model of AP induced by cerulein. **A** The experimental schedule of control and cerulein-induced AP groups (n = 6 each group); **B**–**D** Colorimetric analysis of serum concentration of amylase, lipase and tissue concentration of trypsin; **E** gross appearance of pancreas; **F** The pancreatic injury was determined using HE staining (Scale bar: 100 μm, 20 μm) (black arrows: acinar cell death; blue arrows: acinar vacuolisation; yellow arrows: infiltration of inflammatory cells; green arrows: pancreatic interstitial oedema); **G** Rank of differentially expressed proteins between control and AP groups; **H** Colorimetric analysis of tissue concentration of TNF-α and IFN-γ; **I** Western blotting analysis of FAT10, FTH1, TFRC and ACSL4 expression in the pancreas of rats. **J** Representative immunohistochemical staining of FAT10, FTH1, TFRC and ACSL4 in the pancreas of rats (Scale bar:100 μm, 20 μm); **K** Colorimetric analysis of Fe^2+^ levels and Perls Prussian blue staining of pancrea slices (Scale bar: 20 μm); **L** Colorimetric analysis of MDA levels and MDA staining of pancreas slices (Scale bar: 50 μm). ***p < 0.001.

To investigate whether FAT10 is involved in regulating ferroptosis in pancreatic acinar cells, we first established a rat model of AP using cerulein and measured changes in serum amylase (AMY) and lipase (LPS) levels as well as tissue trypsin activity. Serum AMY and LPS levels, along with tissue trypsin activity, were significantly increased in cerulein-treated rats (Fig. 1A−D). The pancreatic tissue in the cerulein group exhibited morphological changes, including diffuse oedema, increased capsule tension, and surface congestion (Fig. 1E). Hematoxylin and eosin (HE) staining confirmed typical histopathological characteristics of AP in the cerulein group, including acinar cell death, vacuolation, interstitial oedema, and inflammatory cell infiltration, as evidenced by increased histological scores (Fig. 1F). Taken together, these findings indicate that intraperitoneal injection of cerulein successfully induced AP in rats.

Next, we performed a proteomic analysis of pancreatic tissues from the AP and control groups (n = 3 each group). Expression of FAT10 was significantly elevated in pancreatic tissues from the AP group, accompanied by a notable increase in the expression of inflammation-related proteins TNF and IFNG. Concurrently, the expression of ferroptosis-related proteins TFRC and ACSL4 increased, while FTH1 expression significantly decreased (Figs. 1G and S1A). An enzyme-linked immunosorbent assay (ELISA) further confirmed the significant increase in TNF-α and IFN-γ concentrations in pancreatic tissues of the AP group (Fig. 1H). Both western blotting and immunohistochemistry (IHC) analyses revealed upregulation of FAT10, TFRC, and ACSL4 expression, alongside a downregulation of FTH1 expression in pancreatic tissues of the AP group (Figs. 1I, J and S1B). To assess ferroptosis, we measured Fe^2+^ and malondialdehyde (MDA) levels in pancreatic tissues. Colorimetric assays and Perls Prussian blue staining showed a significant increase in Fe^2+^ levels in the AP group compared to the control group (Fig. 1K). Additionally, colorimetric assays and tissue immunofluorescence revealed a marked increase in MDA levels in pancreatic tissues of the AP group (Fig. 1L). Collectively, these results demonstrate a significant upregulation of FAT10 expression and an enhancement of ferroptosis in a cerulein-induced AP rat model.

### Overexpression of FAT10 enhances ferroptosis in pancreatic acinar cells

**Fig. 2:**
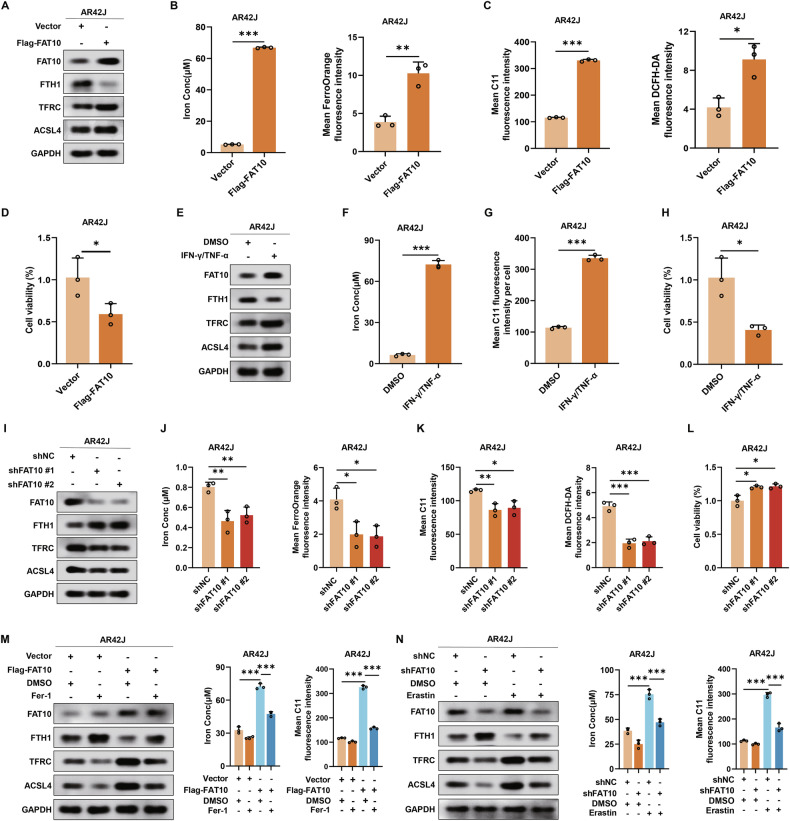
Overexpression of FAT10 enhances ferroptosis in pancreatic acinar cells. **A** Western blotting analysis of FAT10, FTH1, TFRC and ACSL4 expression in control and FAT10-stabled overexpressing AR42J cells; **B** Colorimetric analysis of Fe^2+^ levels and quantification of mean FerroOrange fluoresence intensity in control and FAT10-stabled overexpressing AR42J cells; **C** Quantification of mean C11 fluoresence intensity and mean DCFH-DA fluoresence intensity in control and FAT10-stabled overexpressing AR42J cells; **D** Cell viability of control and FAT10- stabled overexpressing AR42J cells were assessed by CCK-8 assay; **E** Western blotting analysis of FAT10, FTH1, TFRC and ACSL4 expression in DMSO and IFN-γ/TNF-α stimulated AR42J cells; **F** Colorimetric analysis of Fe^2+^ levels in DMSO and IFN-γ/TNF-α stimulated AR42J cells; **G** Quantification of mean C11 fluoresence intensity of DMSO and IFN-γ/TNF-α stimulated AR42J cells stained with C11-BODIPY; **H** Cell viability of DMSO and IFN-γ/TNF-α stimulated AR42J cells were assessed by CCK-8 assay; **I** Western blotting analysis of FAT10, FTH1, TFRC and ACSL4 expression in shNC and shFAT10 transfected AR42J cells; **J** Colorimetric analysis of Fe^2+^ levels and quantification of mean FerroOrange fluoresence intensity in shNC and shFAT10 transfected AR42J cells; **K** Quantification of mean C11 fluoresence intensity and mean DCFH-DA fluoresence intensity of shNC and shFAT10 transfected AR42J cells; L Cell viability of shNC and shFAT10 transfected AR42J cells were assessed by CCK-8 assay; **M** Western blotting analysis of FAT10, FTH1, TFRC and ACSL4 expression in the corresponding groups; colorimetric analysis of Fe^2+^ levels using commercial kits in the corresponding groups and quantification of mean C11 fluoresence intensity of AR42J cells stained with C11-BODIPY in the corresponding groups; **N** Western blotting analysis and quantification of FAT10, FTH1, TFRC and ACSL4 expression in the corresponding groups colorimetric analysis of Fe^2+^ levels using commercial kits in the corresponding groups and quantification of mean C11 fluoresence intensity of AR42J cells stained with C11-BODIPY in the corresponding groups. *p < 0.05; **p < 0.01; ***p < 0.001.

To explore whether FAT10 regulates ferroptosis in pancreatic acinar cells, we first established a stable AR42J cell line with high FAT10 expression (Fig. S2A) and assessed its effects on ferroptosis-related proteins using western blotting analysis. Compared to the vector group, the expression of the ferroptosis-related protein FTH1 decreased in the Flag-FAT10 group, while TFRC and ACSL4 expression levels increased (Figs. 2A and S2B). Further analysis of iron content and FerroOrange staining revealed a significant increase in Fe^2+^ levels in the Flag-FAT10 group (Figs. 2B and S2C). C11-BODIPY and diacetyldichlorofluorescein diacetate staining (DCFH-DA) also showed a notable increase in ROS levels in the Flag-FAT10 group (Figs. 2C and S2D, E). The cell counting kit-8 (CCK-8) assay revealed a significant decrease in cell viability of AR42J cells overexpressing FAT10 (Fig. 2D). Additionally, previous studies have confirmed that IFN-γ/TNF-α can induce endogenous FAT10 expression [27]. Therefore, we treated AR42J cells with IFN-γ/TNF-α and examined changes in ferroptosis-related proteins. Upregulation of endogenous FAT10 expression led to a decrease in FTH1 expression, while TFRC and ACSL4 expression levels increased (Figs. 2E and S2F). Additionally, levels of Fe^2+^ and MDA increased, while cell viability significantly decreased (Figs. 2F–H and S2G). These findings suggest that FAT10 overexpression enhances ferroptosis in pancreatic acinar cells.

To further validate the impact of FAT10 on ferroptosis in pancreatic acinar cells, we used FAT10-targeting short hairpin RNA (shRNA) to reduce FAT10 expression in AR42J cells (Fig. S2H) and assessed changes in ferroptosis. As shown in Figs. 2I and S2I, the downregulation of FAT10 significantly increased FTH1 expression, while TFRC and ACSL4 levels decreased. It also led to reduced levels of Fe^2+^ and ROS, along with increased cell viability (Figs. 2J−L and S2J–L). Furthermore, rescue experiments revealed that treatment with a ferroptosis inhibitor Fer-1 did not affect FAT10 expression but mitigated the increase in Fe^2+^ and ROS levels caused by FAT10 overexpression (Figs. 2M and S2M). Additionally, knockdown of FAT10 expression alleviated the increase in Fe^2+^ and ROS levels induced by a ferroptosis activator erastin (Figs. 2N and S2N). Collectively, these results demonstrate that FAT10 plays a critical role in modulating ferroptosis in pancreatic acinar cells.

### FAT10 regulates NCOA4 to promote ferroptosis in pancreatic acinar cells

**Fig. 3:**
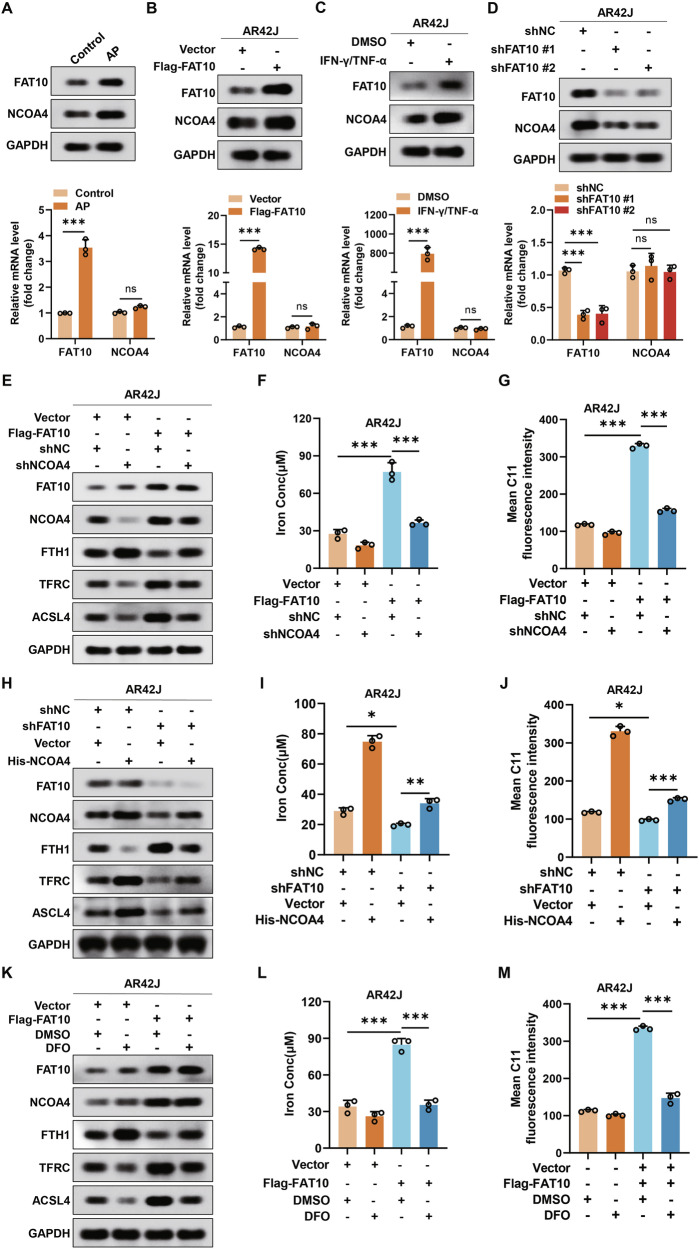
FAT10 regulates NCOA4 to promote ferroptosis in pancreatic acinar cells. **A** Western blotting and qRT-PCR analysis of FAT10 and NCOA4 expression in control and AP pancreatic tissues; **B** Western blotting and qRT-PCR analysis of FAT10 and NCOA4 expression in control and FAT10-stabled overexpressing AR42J cells. **C** Western blotting and qRT-PCR analysis of FAT10 and NCOA4 expression in DMSO and IFN-γ/TNF-α stimulated AR42J cells; **D** Western blotting and qRT-PCR analysis of FAT10 and NCOA4 expression in shNC and shFAT10 transfected AR42J cells; **E** Western blotting analysis of FAT10, NCOA4, FTH1, TFRC, ACSL4 expression in control and FAT10-stabled overexpressing AR42J cells, with or without shNCOA4; **F** Colorimetric analysis of Fe^2+^ levels in control and FAT10-stabled overexpressing AR42J cells, with or without shNCOA4; **G** Quantification of mean C11 fluoresence intensity of control and FAT10-stabled overexpressing AR42J cells, with or without NCOA4 knokdown; **H** Western blotting analysis of FAT10, NCOA4, FTH1, TFRC, ACSL4 expression in shNC and shFAT10 transfected AR42J cells, with or without NCOA4 overexpression; **I** Colorimetric analysis of Fe^2+^ levels in shNC and shFAT10 transfected AR42J cells, with or without NCOA4 overexpression. **J** Quantification of mean C11 fluoresence intensity of shNC and shFAT10 transfected AR42J cells, with or without NCOA4 overexpression; **K** Western blotting analysis of FAT10, NCOA4, FTH1, TFRC, ACSL4 expression in control and FAT10-stabled overexpressing AR42J cells, with or without DFO treatment; **L** Colorimetric analysis of Fe^2+^ levels in control and FAT10-stabled overexpressing AR42J cells, with or without DFO treatment; **M** Quantification of mean C11 fluoresence intensity of control and FAT10-stabled overexpressing AR42J cells, with or without DFO treatment. *p < 0.05; **p < 0.01; ***p < 0.001. ns not significant.

Studies have demonstrated that NCOA4 plays an important role in ferroptosis by regulating iron levels [41, 42]. Therefore, we explored whether FAT10 promotes ferroptosis in pancreatic acinar cells by modulating NCOA4. Western blotting and quantitative reverse transcription PCR (qRT-PCR) analyses revealed that FAT10 protein and mRNA levels were significantly elevated in pancreatic tissues of rats with cerulein-induced AP, while NCOA4 protein levels increased without a corresponding change in mRNA expression (Fig. 3A). Next, we investigated the relationship between FAT10 and NCOA4 expression in AR42J cells by modulating FAT10 expression. Western blotting and qRT-PCR experiments showed that compared to the vector group, NCOA4 protein expression increased in the Flag-FAT10 group, while there was no significant change in NCOA4 mRNA levels (Fig. 3B). Similar results were observed when IFN-γ/TNF-α was used (Fig. 3C). In contrast, compared to the negative control (shNC) groups, NCOA4 protein levels were reduced in the shFAT10 groups of pancreatic acinar cells, with no significant change in mRNA expression (Fig. 3D). These findings indicate that FAT10 promotes ferroptosis in pancreatic acinar cells by regulating NCOA4 expression at the protein level.

To further investigate whether FAT10 regulates ferroptosis in pancreatic acinar cells via NCOA4, we performed rescue experiments. We knocked down NCOA4 expression in Flag-FAT10 AR42J cells and assessed the expression of ferroptosis-related proteins using western blotting. NCOA4 knockdown reversed the elevated levels of NCOA4, TFRC, and ACSL4 proteins induced by FAT10 overexpression, as well as the reduction in FTH1 protein levels (Fig. 3E). Furthermore, NCOA4 knockdown mitigated the increase in Fe^2+^ and ROS levels induced by FAT10 overexpression (Figs. 3F, G and S3A). In contrast, NCOA4 overexpression could block the decrease in ferroptosis caused by FAT10 knockdown and also reversed the reduction in Fe^2+^ and ROS levels following FAT10 knockdown (Figs. 3H−J and S3B). Finally, treatment with DFO, an iron chelator, did not affect FAT10 and NCOA4 protein expression; however, it could reverse the increase in NCOA4, TFRC, and ACSL4 protein levels, as well as the decrease in FTH1 protein levels caused by FAT10 overexpression (Fig. 3K). Additionally, DFO mitigated the increase in Fe^2+^ and ROS levels induced by FAT10 overexpression (Figs. 3L, M and S3C). Taken together, these findings confirm that FAT10 enhances ferroptosis in pancreatic acinar cells primarily by upregulating NCOA4 expression.

### FAT10 stabilises NCOA4 expression by inhibiting its ubiquitination in pancreatic acinar cells

**Fig. 4:**
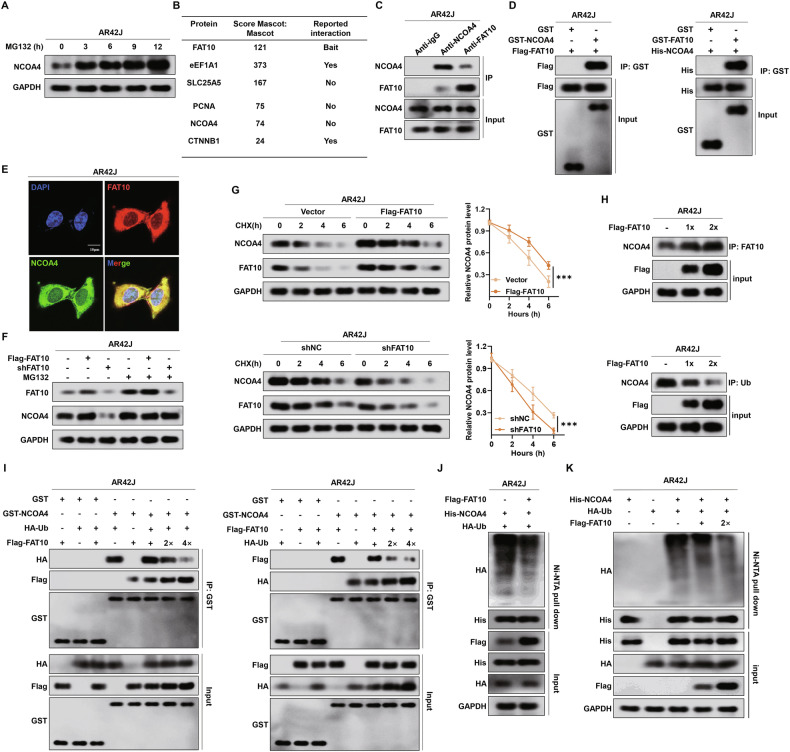
FAT10 stabilises NCOA4 expression by inhibiting its ubiquitination in pancreatic acinar cells. **A** AR42J cells were treated with MG132 (15 μM) for the indicated time, NCOA4 levels were detected by western blotting; **B** Quantitive shotgun analysis showed substrate proteins interaction with FAT10; **C** Co-IP for FAT10 and NCOA4 in AR42J cells; **D** GST-pull-down analysis for FAT10 and NCOA4 in AR42J cells; **E** Colocalisation of FAT10 and NCOA4 in AR42J cells (Scale bar: 10 μm); **F** Western blotting analysis of FAT10 and NCOA4 expression in AR42J cells transfected with the indicated plasmids treated with or without MG132 (15 μM); **G** The cells were exposed to CHX (20 μM) at the indicated times, and the degradation of exogenous FAT10 and NCOA4 was detected in AR42J cells transfected with the indicated plasmids; **H** AR42J cells were transfected with increasing amounts of Flag-FAT10 plasmid. The cells were lysed for immunoprecipitation using anti-Ub and anti-FAT10 beads to detect NCOA4 binding; **I** Binding of NCOA4 during the course of the competition was analysed by GST-pull-down experiments; **J**, **K** AR42J cells transfected with the indicated plasmid were treated with MG132 (15 μM) and the ubiquitination of NCOA4 was detected by western blotting analysis. ***p < 0.001.

We investigated the molecular mechanisms by which FAT10 regulates NCOA4 protein expression. Our previous studies demonstrated that FAT10 modulates the expression of substrate proteins by suppressing their ubiquitination [23, 33]. Studies have also confirmed that NCOA4 is degraded via the ubiquitin-proteasome pathway [16, 43]. Consistently, treatment with the proteasome inhibitor MG132 led to significant accumulation of endogenous NCOA4 protein in AR42J cells, suggesting that NCOA4 is degraded via the proteasome pathway in pancreatic acinar cells (Fig. 4A). Therefore, we hypothesised that FAT10 regulates NCOA4 expression by inhibiting its ubiquitination. Mass spectrometry analysis of proteins interacting with FAT10 in AR42J cells identified NCOA4 as a binding partner (Fig. 4B). Coimmunoprecipitation (Co-IP) analysis confirmed the interaction between FAT10 and NCOA4 in rat primary pancreatic acinar cells (rPPACs) and AR42J cells (Figs. 4C and S4A). Purified glutathione S-transferase (GST) pull-down experiment revealed that NCOA4 bind to FAT10 in vitro (Fig. 4D). Confocal microscopy confirmed the colocalisation of FAT10 and NCOA4 in AR42J cells, providing additional evidence of an interaction between these proteins (Fig. 4E). These findings indicate that FAT10 directly binds to NCOA4 in pancreatic acinar cells.

Next, to determine whether FAT10 is involved in regulating the degradation of NCOA4 protein, we transfected shFAT10 and Flag-FAT10 plasmids into AR42J cells and assessed the effects of varying FAT10 levels on NCOA4 expression, with or without MG132 treatment. Decreasing or increasing FAT10 expression had no significant effect on NCOA4 expression after MG132 treatment (Fig. 4F). The degradation dynamics assay showed that the half-life of exogenously expressed NCOA4 was significantly increased in FAT10-overexpressing pancreatic acinar cells compared to that in control cells, while FAT10 knockdown significantly reduced NCOA4’s half-life (Fig. 4G). Furthermore, we investigated whether FAT10 inhibits NCOA4 ubiquitination. In our previous study, we confirmed that FAT10 inhibits the ubiquitination of substrates by competing with ubiquitin for substrate binding. Our data indicated that overexpressed FAT10 can compete with ubiquitin to bind NCOA4, leading to increased formation of the FAT10-NCOA4 complex (Fig. 4H). A GST-pull-down assay showed that as FAT10 levels increased, the FAT10-NCOA4 complex level also gradually increased, whereas those of ubiquitin-NCOA4 complexes gradually decreased (Fig. 4I). Besides, FAT10 overexpression led to a substantial decrease in NCOA4 polyubiquitination (Fig. 4J). We also found that the decrease in NCOA4 polyubiquitination level was dose-dependent, with an increase in FAT10 (Fig. 4K). Taken together, our data confirm that FAT10 stabilises NCOA4 protein expression by inhibiting its ubiquitination in AR42J cells.

### FAT10-NCOA4 complex is not degraded by the proteasome pathway

**Fig. 5:**
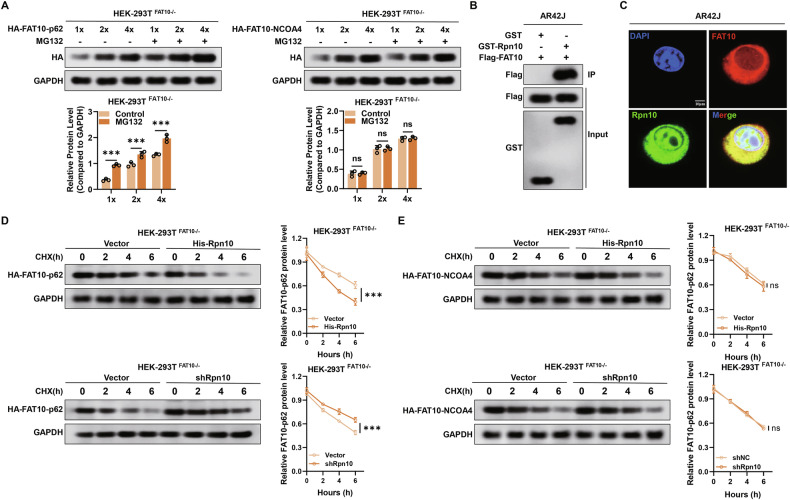
FAT10-NCOA4 complex is not degraded by the proteasome pathway. **A** HA-FAT10-p62 and HA-FAT10-NCOA4 fusion plasmids were transfected into FAT10-/- HEK293T cells at different doses. The expression of HA-FAT10-p62 and HA-FAT10-NCOA4 complexes was observed with or without MG132 (15 μM); **B** GST- pull-down analysis for FAT10 and RPN10 in AR42J cells; **C** Colocalisation of FAT10 and RPN10 in AR42J cells (Scale bar: 10 μm); **D**, **E** FAT10-/- HEK293T cells were transfected with HA-FAT10-p62 and HA-FAT10-NCOA4 plasmids combined with His-Rpn10 or shRpn10 plasmids and were then exposed to cycloheximide (CHX, 20 μM). Western blotting analysis was performed for the indicated time and quantified by ImageJ software. ***p < 0.001. ns not significant.

Our previous studies in HEK293T cells have shown that FAT10 competes with ubiquitin to bind eukaryotic translation elongation factor 1A1 (eEF1A1) to form a FAT10-eEF1A1 complex, which is not recognised by the FAT10 receptor RPN10 in the 26S proteasome and is not degraded by proteasomes, resulting in increased eEF1A1 protein expression [23]. In this study, we aimed to investigate whether the FAT10-NCOA4 complex is recognised by RPN10 or evades proteasomal degradation. Therefore, we constructed HA-FAT10-p62 and HA-FAT10-NCOA4 fusion plasmids. Using the FAT10-p62 complex as a positive control, we assessed whether FAT10-NCOA4 complexes undergo degradation by the proteasome. In FAT10 knockout (FAT10-KO) HEK293T cells, HA-FAT10-p62 expression significantly increased with MG132, while HA-FAT10-NCOA4 levels remained unchanged, regardless of MG132 treatment (Fig. 5A). These results indicate that HA-FAT10-p62 is degraded by the proteasome, whereas HA-FAT10-NCOA4 is not. To further confirm that FAT10-NCOA4 is resistant to proteasomal degradation, we first demonstrated that RPN10 binds to FAT10 in pancreatic acinar cells (Fig. 5B, C). Next, we examined the degradation rates of FAT10-p62 and FAT10-NCOA4 in response to altered RPN10 expression in FAT10-KO HEK293T cells. In RPN10-overepxressing cells, the half-life of ectopically expressed HA-FAT10-p62 decreased significantly, compared to that in control cells, while RPN10 significantly reduced HA-FAT10-p62 degradation rates. However, RPN10 manipulation had no effect on the degradation rate of HA-FAT10-NCOA4 (Fig. 5D, E). Collectively, these findings demonstrate that FAT10-NCOA4 complexes are not degraded by proteasomes.

### Silibinin inhibits the FAT10-NCOA4 axis to reduce ferroptosis in vitro and in vivo

**Fig. 6:**
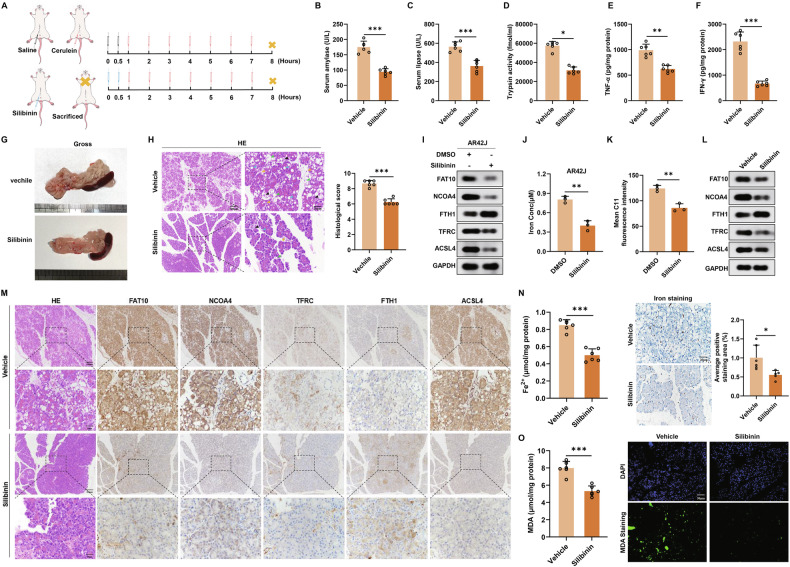
Silibinin inhibits the FAT10-NCOA4 axis to reduce ferroptosis in vitro and in vivo. **A** The experimental schedule of cerulein-induced AP with saline and Silibinin pretreatment (n = 6 each group); **B**–**D** Colorimetric analysis of serum concentration of amylase, lipase and tissue concentration of trypsin; **E**, **F** Colorimetric analysis of tissue concentration of TNF-α and IFN-γ; **G** Gross appearance of pancreas; **H** The pancreatic injury was determined by HE staining. (Scale bar: 100 μm, 20 μm) (black arrows: acinar cell death; blue arrows: acinar vacuolisation; yellow arrows: infiltration of inflammatory cells; green arrows: pancreatic interstitial oedema); **I** Western blotting analysis of FAT10, NCOA4, FTH1, TFRC and ACSL4 expression in DMSO and Silibinin-treated AR42J cells; **J** Colorimetric analysis of Fe^2+^ levels in DMSO and Silibinin-treated AR42J cells; **K** Quantification of mean C11 fluoresence intensity of DMSO and Silibinin-treated AR42J cells stained with C11-BODIPY; **L** Western blotting analysis of FAT10, NCOA4, FTH1, TFRC and ACSL4 expression in the pancreas of rats; **M** Representative immunohistochemical staining of FAT10, NCOA4, FTH1, TFRC and ACSL4 in the pancreas of rats. (Scale bar: 100 μm, 20 μm); **N** Colorimetric analysis of Fe^2+^ levels and Perls Prussian blue staining of pancrea slices (Scale bar: 20 μm); **O** Measurement of MDA levels and MDA staining of pancrea slices (Scale bar: 50 μm). *p < 0.05; **p < 0.01; ***p < 0.001.

Finally, we identified a drug that targeted FAT10 to suppress NCOA4-induced ferroptosis in pancreatic acinar cells, thereby improving AP. Silibinin is a flavonoid extracted from the milk thistle (*Silybum marianum*) plant [44]. Studies have confirmed that silibinin inhibits FAT10 expression in tumour cells [25, 44]. Therefore, we investigated whether silibinin could effectively improve AP by assessing its effect on tissue damage in a rat model. Compared to the control group, rats treated with silibinin exhibited significantly decreased serum AMY and LPS levels, as well as reduced pancreatic tissue trypsin activity (Fig. 6A−D). Additionally, concentrations of inflammatory markers TNF-α and IFN-γ in pancreatic tissue were significantly decreased in the silibinin group (Fig. 6E, F). Moreover, the silibinin-treated group showed a reduction in diffuse oedema, capsule tension, and surface congestion in the pancreatic tissue (Fig. 6G). Additionally, HE staining revealed significant improvements in pancreatic interstitial oedema, acinar vacuolisation, acinar cell death, and inflammatory cell infiltration in the silibinin group, along with a marked decrease in pathological scores (Fig. 6H). These findings demonstrate that silibinin effectively reduces inflammation and tissue damage associated with AP in rats.

Next, we examined whether silibinin inhibits the FAT10-NCOA4 axis to suppress ferroptosis, potentially improving AP. After treating AR42J cells with silibinin, we analysed changes in the expression of FAT10, NCOA4, and ferroptosis-related proteins using western blotting. In silibinin-treated AR42J cells, the expression of FAT10 and NCOA4 proteins decreased, whereas the expression of the ferroptosis-related protein FTH1 increased. The expression of ACSL4 and TFRC proteins also decreased (Fig. 6I). Furthermore, we found that the levels of Fe^2+^ and ROS decreased in silibinin-treated AR42J cells (Figs. 6J, K and S5A). These findings suggest that silibinin inhibits ferroptosis in pancreatic acinar cells by suppressing the FAT10-NCOA4 axis in vitro. Subsequently, we examined the effects of silibinin on FAT10, NCOA4, and ferroptosis-related proteins in a rat model using western blotting and IHC. Compared to the control group, the expression of FAT10, NCOA4, TFRC, and ACSL4 decreased in the silibinin-treated group, while the expression of FTH1 increased (Figs. 6L, M and S5B). Additionally, colorimetric assays and tissue immunofluorescence analyses revealed that the levels of Fe^2+^ and MDA were significantly reduced in the silibinin-treated group (Fig. 6N, O). Taken together, these findings suggest that silibinin inhibits ferroptosis by inhibiting the FAT10-NCOA4 axis, thereby alleviating inflammatory responses and reducing tissue damage in AP.

## Discussion

AP is characterised by inflammation of the pancreas, often triggered by factors such as gallstones, alcohol abuse, drug reactions, or metabolic abnormalities. It presents as inflammation-mediated pancreatic damage, typically leading to systemic inflammatory response syndrome (SIRS) and multiple organ dysfunction (MODS), increasing patient mortality rates [1]. A central event in AP pathology is the death of pancreatic acinar cells, which occurs through complex mechanisms involving factors such as abnormal activation of enzymes, release of inflammatory mediators, oxidative stress, microcirculatory disturbances, and ferroptosis [3]. The relationship between ferroptosis and AP is particularly significant, as the accumulation of iron and ROS can exacerbate pancreatic inflammation and tissue damage [11]. This study presents compelling evidence that FAT10 is a critical regulator of ferroptosis in AP. Our findings suggest that FAT10 enhances ferroptosis by interacting with NCOA4, a key regulator of iron metabolism, thereby contributing to the pathophysiology of AP. This relationship provides new insights into potential therapeutic targets for managing AP, a condition frequently associated with high morbidity and mortality.

Ubiquitin-like proteins share a sequence and structural similarities with ubiquitin and modulate protein expression and function via enzyme-catalysed cascades that modify to substrate proteins, affecting various cellular processes [20]. Research has shown that ubiquitin-like proteins play an important role in cellular ferroptosis. For instance, SUMO1 can promote the SUMOylation of ACSL4, stabilising its expression and leading lipid peroxide accumulation, and inducing ferroptosis in squamous carcinoma cells, which drives head and neck squamous cell carcinoma progression [45]. Metformin can inhibit the expression of the ubiquitin-like protein UFM1, which in turn suppresses the UFMylation of SLC7A11, leading to its reduced expression and induction of ferroptosis in breast cancer cells, thereby inhibiting cancer progression [46]. As a ubiquitin-like protein, FAT10 performs various functions in the regulation of diverse cellular functions. However, there have been no reports on FAT10’s involvement in ferroptosis in pancreatic acinar cells during AP. In this study, we found that the levels of FAT10 and ferroptosis markers significantly increased in a rat model of AP. Further investigation revealed that FAT10 is a key factor in the regulation of ferroptosis in pancreatic acinar cells. Overexpression of FAT10 enhances ferroptosis in pancreatic acinar cells, whereas its knockdown reverses the increase in Fe^2+^ and ROS levels induced by the ferroptosis activator erastin. To the best of our knowledge, this is the first study to report the regulatory role of FAT10 in cellular ferroptosis, expanding our understanding of FAT10 functions.

NCOA4 is an important nuclear receptor coactivator that was initially discovered through yeast two-hybrid screening for to its ability to bind the oestrogen receptor (ER) and enhance transcriptional activity [47]. Recent studies have shown that NCOA4 plays a crucial role in iron metabolism, particularly as the sole selective autophagy receptor that mediates ferritin degradation in autophagosomes [41, 48]. NCOA4 co-localises with the autophagy-related protein LC3B, promoting ferritin autophagy and leading to the accumulation of intracellular iron, a classic pathway for mediating ferroptosis [48]. Recent studies have indicated that the regulation of NCOA4-mediated ferroptosis has significant implications for various diseases. For instance, ZNF350 enhances ferroptosis in glioma cells by reducing transcriptional repression of NCOA4, thereby promoting glioma progression, elevating STING levels to increase NCOA4 binding, enhancing ferroptosis in renal tubular epithelial cells, and exacerbating acute kidney injury [49, 50]. Conversely, reduced YAP levels promote the binding of NCOA4 to FTH1, intensifying hepatocyte ferroptosis in non-alcoholic fatty liver disease [51]. However, whether NCOA4 mediates ferroptosis in pancreatic acinar cells and the underlying regulatory mechanisms remain unclear. Our study revealed that FAT10 levels were significantly elevated in the pancreatic tissues of rats with AP, correlating with increased NCOA4 protein levels. Besides, we demonstrated that overexpression of FAT10 led to increased NCOA4 protein levels. Furthermore, rescue experiments indicated that FAT10 enhanced ferroptosis by promoting NCOA4 expression, as knocking it down reversed the effects on ferroptosis-related proteins and mitigated the increase in Fe^2+^ and ROS levels. Additionally, NCOA4 overexpression counteracted the reduction in ferroptosis observed following FAT10 knockdown. Taken together, these results demonstrate that FAT10 is a key regulator of NCOA4-mediated ferroptosis in pancreatic acinar cells.

Next, we investigated the mechanism through which FAT10 regulates NCOA4 expression. The ubiquitin-proteasome-mediated degradation of NCOA4 is a key mechanism regulating NCOA4 levels in cells [43]. As a multifunctional regulatory factor, FAT10 profoundly affects cell fate and function through its role in protein degradation. FAT10 is currently the only known ubiquitin-like protein to possess a dual function: being able to directly degrade as well as stabilise substrates. This unique ability allows FAT10 to play a key role in regulatory networks within cells by adjusting the fate of substrates based on cellular needs [23, 28]. Our previous studies have confirmed that FAT10 plays a significant role in stabilising substrate proteins in cardiovascular diseases and cancer. For instance, FAT10 protects against ischaemia-induced ventricular arrhythmia by binding to Nav1.5, preventing its degradation by the ubiquitin-proteasome system (UPS) after myocardial infarction (MI) [24]. In hepatocellular carcinoma, FAT10 promotes invasion and metastasis by stabilising β-catenin [31]. Moreover, FAT10 limits the efficacy of anti-VEGF therapy for hepatocellular carcinoma by simultaneously stabilising multiple proteins, including HIF1α, β-catenin, STAT3, and TAB3 proteins [33]. In this study, we discovered a new mechanism that regulates NCOA4 expression, in which FAT10 stabilises NCOA4 protein expression in pancreatic acinar cells in a complex manner. FAT10 antagonises NCOA4 ubiquitination to increase its expression by decreasing ubiquitin–eEF1A1 complexes. In contrast, the FAT10-NCOA4 complex is not degraded by the proteasome, leading to stabilisation of NCOA4 protein expression by FAT10. This conclusion is based on several key observations: First, the expression of NCOA4 was increased in pancreatic acinar cells, and reducing the expression of FAT10 in these cells reduced NCOA4 protein levels without affecting its mRNA expression. Second, FAT10 overexpression reduced the formation of ubiquitin-NCOA4 complexes, decreasing NCOA4 ubiquitination and enhancing NCOA4 expression. Moreover, FAT10 competes with ubiquitin (Ub) for binding to the same sites on substrate proteins, thereby stabilising them [23, 31]. The stabilisation of NCOA4 by FAT10 may also involve competition at these binding sites. Third, the expression of FAT10-NCOA4 was not affected by the presence or absence of MG132, indicating that this complex was not degraded by the proteasome. Finally, altering RPN10 expression did not affect the degradation rate or accumulation of FAT10-NCOA4. In addition, our previous study demonstrated that FAT10 can stabilise β-Catenin independent of the diglycine (GG) at its C-terminus [32]. In this study, we investigated the relationship between the diglycine (GG) mutation at the C-terminus of FAT10, the NCOA4 protein, and ferroptosis. Immunoprecipitation results indicated that even after the mutation of the diglycine (GG) at the C-terminus of FAT10, it can still interact with the NCOA4 protein. Transfection of the Flag-FAT10ΔGG plasmid into pancreatic acinar cells resulted in increased NCOA4 expression, which led to elevated lipid ROS levels and ultimately exacerbated ferroptosis in these cells (Fig. S6A–I).

Importantly, we identified silibinin, a potential therapeutic agent that could improve the pathology of AP. Silibinin is the active component of silymarin, a complex of flavonolignans extracted from milk thistle seeds [44]. Clinical studies have indicated that silibinin exerts protective effects against various inflammation-related diseases including hepatitis, cardiovascular disease, and metabolic syndrome [25, 52]. It possesses significant anti-inflammatory properties and inhibits inflammatory responses through multiple mechanisms, such as modulating pro-inflammatory cytokines such as TNF-α and IFN-γ and inhibiting signalling pathways like NF-κB [44, 53]. Recent studies suggest that silibinin effectively downregulates the expression of FAT10, which plays a pivotal role in inflammatory responses [25, 44]. Here, we identified silibinin as an effective inhibitor of the FAT10-NCOA4 axis. By inhibiting this axis, silibinin not only mitigates inflammation, but also reduces ferroptosis, a form of regulated cell death associated with pancreatic damage. Our results indicated that silibinin treatment led to decreased levels of ferroptosis-related proteins (TFRC and ACSL4) and inflammatory markers in both cellular and animal models, highlighting its potential as a therapeutic strategy for alleviating pancreatic injury. Therefore, our study underscores the potential of silibinin as a multifaceted therapeutic agent for AP. Its ability to modulate inflammatory pathways and reduce ferroptosis presents a promising avenue for the development of novel treatments for treating pancreatic injury and improve patient outcomes in inflammation-related disorders.

**Fig. 7:**
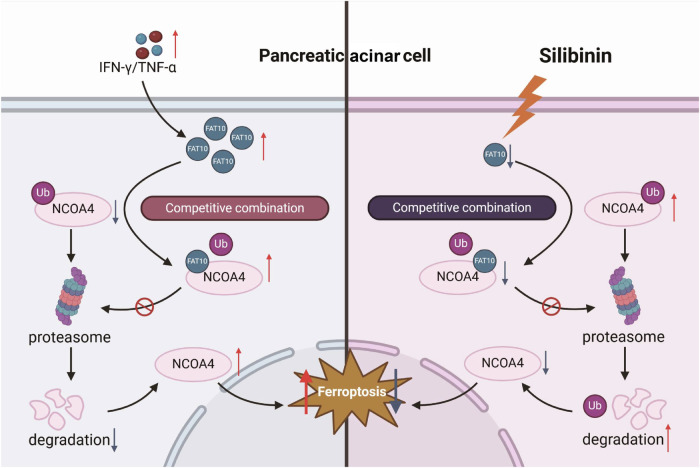
A cartoon summarising our findings. During the progression of AP, FAT10 expression is upregulated. It competes with ubiquitin for binding to NCOA4, thereby antagonising its ubiquitination and forming a stable FAT10-NCOA4 complex that is not degraded via the proteasomal pathway. This results in increased NCOA4 expression, which in turn promotes ferroptosis in pancreatic acinar cells. Silibinin, by targeting and inhibiting the FAT10-NCOA4 pathway, can suppress ferroptosis in pancreatic acinar cells.

In summary, this study elucidates the critical role of FAT10 in the regulation of ferroptosis in pancreatic acinar cells during AP. We established a mechanistic link between FAT10 and NCOA4, both of which are essential in iron metabolism, suggesting that FAT10 enhances ferroptosis by elevating NCOA4 expression. Our findings demonstrate that FAT10 stabilises NCOA4 through a unique mechanism that prevents its ubiquitination and proteasomal degradation, contributing to increased iron accumulation and oxidative stress in pancreatic tissues. Notably, we identified silibinin as a promising therapeutic agent that inhibits the FAT10-NCOA4 axis, effectively reducing both inflammation and ferroptosis in models of AP (Fig. 7). Overall, targeting the FAT10-NCOA4 pathway offers a promising new direction for the development of treatments for AP and its associated complications.

## Methods

### Animals

Male Sprague-Dawley rats (6–8 weeks, 200–250 g) were sourced from GemPharmatech Co., Ltd. (Jiangsu, China). The rats were bred and maintained under standard laboratory conditions at 22 ± 1 °C and a 12-h light/dark cycle. Animal care and handling procedures adhered to the Guide for the Care and Use of Laboratory Animals and received approval from the Ethics Committee for Animal Research at Nanchang University (Nanchang, China) (Approval no. NCULAE-20221031128).

### The AP rat model and silibinin pretreatment

Rats in the cerulein-induced AP group (Ceru, n = 6) were administered a total of 50 μg/kg cerulein (HY-A0190, MedChemExpress) via 7 intraperitoneal (i.p.) injections, with a 1-h interval. Rats in the control group (n = 6) were given an equivalent volume of saline. Rats in the silibinin treatment group (Sili + Ceru, n = 6) were pretreated with 100 mg/kg silibinin (S2357, Selleck) via 2 i.p. injections within 1 h before the first cerulein injection. The animals were sacrificed 1 h after the final cerulein injection, and the pancreas, spleen, and blood were harvested for gross examination and subsequent analysis.

### Label-free liquid chromatography-tandem mass spectrometry (LC-MS/ MS) analysis

Fresh pancreatic samples from the control and AP groups were collected and placed in 15 mL centrifuge tubes, then immediately stored on dry ice. Protein expression was analysed using label-free LC-MS/MS (Personalbio, Shanghai, China).

### Cells and cell culture

The rat pancreatic acinar cell line, AR42J, and human embryonic kidney cell line, HEK293T, were obtained from the Shanghai Cell Bank, Type Culture Collection Committee of the Chinese Academy of Sciences (Shanghai, China), and authenticated by short tandem repeat profiling at the Cell Bank. All cell lines were free of mycoplasmaCells were cultured in DMEM (Gibco, Grand Island, NY, USA) supplemented with 10% foetal bovine serum (FBS) (Gibco, Grand Island, NY, USA) at 37 °C in 5% CO₂. Extraction and culture of rat primary pancreatic acinar cells (rPPACs) as follows. Fresh rat pancreatic tissues were washed in serum-free Ham’s F12 medium. The excised tissues and isolated cells were digested with type II collagenase, filtered sequentially through 100-mesh and 200-mesh filters, and the cells were resuspended in complete medium. Cells were cultured in F12 medium containing 10% FBS at 37 °C in 5% CO₂.

### In vivo ubiquitination assay

Cells transfected with expression construct for His-NCOA4, HA-Ub and Flag-FAT10 were treated with MG132 for 12 h and lysed under denaturing conditions by Binding/Wash buffer (C601005-0500, Sangon). Lysates were incubated twice with Ni-NTA agarose (C600791, Sangon) and then washed with Elution Buffer (20 mM Tris-HCl, 8 M urea, 500 mM NaCl, 500 mM imidazole and pH 8.0), followed by Western blot analysis.

### Coimmunoprecipitation (Co-IP)

Cells were lysed in ice-cold lysis buffer for western blotting and immunoprecipitation (P0013, Beyotime) and centrifuged at 12,000 × *g* for 15 min to remove debris. The cleared lysates were then subjected to immunoprecipitation with either irrelevant IgG (#5415, Cell Signaling Technology) or specific antibodies (FAT10, 13003-2-AP, Proteintech; NCOA4, ab86707, Abcam), and protein A/G PLUS-Agarose (sc-2003, Santa Cruz Biotechnology). The samples were incubated at 4 °C overnight. After three washes with wash buffer, the proteins were separated using an appropriate Bis-Tris gel for SDS-PAGE, followed by western blotting analysis.

### Shotgun LC-MS/ MS analysis

FAT10 was immunoprecipitated from the lysates of AR42J cells for the co-IP assay. Samples were cultured in electrophoresis sample buffer and underwent electrophoresis on sodium dodecyl sulphate-polyacrylamide gels. The proteins resolved from the gels were analysed by shotgun LC-MS/ MS (GeneChem, Shanghai, China).

### GST-pull-down assay

GST, GST-FAT10, and GST-NCOA4 were expressed in *Escherichia coli* strain BL21. Glutathione Sepharose 4B beads (Sigma-Aldrich) were incubated overnight at 4 °C with GST, GST-FAT10, or GST-NCOA4, along with purified His-NCOA4 or Flag-FAT10 (Enzo Life Sciences) in an incubation buffer (20 mmol/L Tris-HCl, pH 7.4, 0.1% Triton X-100). After four washes with the same buffer, Bound proteins were eluted by boiling at 95 °C for 5 min in SDS buffer, separated by SDS-PAGE, and analysed using western blotting.

### Immunofluorescence staining

Cells were washed, methanol-fixed, rinsed thrice with PBS, and blocked for 15 min with PBS containing 5% goat serum. Cells were incubated overnight at 4 °C with anti-FAT10 (ab168680; Abcam) and anti-NCOA4 (ab86707; Abcam), followed by three PBS washes. Subsequently, the cells were stained with Alexa Fluor 546- or 488-conjugated secondary antibodies (1:100 dilution) for 2 h at room temperature. After three PBS washes, cells were stained with the DAPI nuclear stain for 10 s. After three PBS washes again, the cells were visualised using a Leica SP-II confocal laser scanning microscope.

### Plasmids and lentivirus

Lentiviral vectors for silencing and overexpressing FAT10 (shFAT10 and Flag-FAT10), the NCOA4-overexpressing and shNCOA4-expressing plasmids, as well as the RPN10-overexpressing and shRPN10-expressing plasmids, were purchased from GeneChem (Shanghai, China). The target sites of shRNA were as provided in Table S2. For the deficiency of C-terminal double diglycine motif of FAT10 plasmid (FAT10ΔGG), a FAT10 DNA sequence without C-terminal double diglycine motif was synthesised by GeneChem.

### Generation of stable transfected cells

The day before transfection, 1 × 10⁵ AR42J cells were seeded into 6-well plates. Cells were seeded to 70% confluence 12 h before infection, and the cell culture medium was replaced with medium containing the indicated lentivirus. After 12 h of infection, the medium was replaced with fresh medium, and 48 h later, the infected cells were selected using 10 µg/mL puromycin (InvivoGen, San Diego, CA, USA). To select for stably transfected cells, cells were cultured in high-glucose DMEM with 5 µg/mL puromycin for 4 weeks. Clones demonstrating puromycin resistance were selected and expanded.

### RNA extraction and quantitative real-time polymerase chain reaction (qRT-PCR)

Total RNA was extracted from cultured cells using TRIzol reagent (15596026, Invitrogen) following the manufacturer’s instructions. The isolated RNA was reverse-transcribed into complementary DNA (cDNA) using the PrimeScript RT reagent kit (RR036A, Takara). The cDNA was then subjected to qRT-PCR analysis with TB Green® Fast qPCR Mix (RR430A, Takara) and a QuantStudio5 (Applied Biosystems). Specific primers were synthesised by Tsinke (Beijing, China), and the detailed sequences were as provided in Table S2.

### Western blotting

Total protein was extracted from cells or pancreatic specimens, and equal amounts of protein were separated via sodium dodecyl sulphate-polyacrylamide gel electrophoresis (SDS-PAGE). Proteins were transferred to a nitrocellulose membrane (Millipore, Bedford, MA, USA) via electroblotting. After overnight incubation with anti-FAT10 (1:500, 13003-2-AP, Proteintech), anti-NCOA4 (1:1000, ab86707, Abcam), anti-ACSL4 (1:1000, 22401-1-AP, Proteintech), anti-TFRC (1:500, sc-393719, Santa Cruz Biotechnology), and anti-FTH1 (ab183781, Abcam) at 4 °C, the membranes were washed three times with 1×TBST and incubated with horseradish peroxidase-conjugated goat anti-rabbit or anti-mouse IgG secondary antibodies (ZB-2301 or ZB-2305, 1:10 000, ZSGB Biotechnology) for 1 h at room temperature. The membranes were then washed three more times with 1×TBST, and specific antibody interactions were visualised by chemiluminescence (Thermo, Waltham, MA, USA). The intensity of each band was measured using ImageJ 1.53e (National Institutes of Health, USA).

### Cell viability

Cell viability was determined using the CCK-8 assay. AR42J cells (5 × 10³ cells per well) were seeded in a 96-well plate and transfected with Flag-FAT10 or shFAT10, then cultured for 24 h. Subsequently, cell viability was assessed using a CCK-8 kit (HY-K0301, MedChemExpress) at 36 h, incubating the cells with 10 µL CCK-8 solution at 37 °C for 4 h. Determined the absorbance at 450 nm by a Varioskan LUX multimode microplate reader (Thermo Fisher).

### Enzyme-linked immunosorbent assay (ELISA)

Commercially available ELISA kits were used to measure the concentrations or activity of IFN-γ (SEKR-0008, Solarbio), TNF-α (SEKR-0009, Solarbio) in the indicated samples following the manufacturer’s instructions. Determined the absorbance by a Varioskan LUX multimode microplate reader (Thermo Fisher).

### Immunohistochemistry (IHC)

Sections of pancreatic tissue were treated with graded xylene and alcohol, respectively, and then subjected to antigen retrieval in 0.01 M citrate buffer. The sections were blocked with endogenous peroxidase for 10 min, then incubated with goat serum for 30 min, and finally with anti-FAT10 (1:350, 13003-2-AP, Proteintech), anti-NCOA4 (1:1000, PA5-115626, Invitrogen), anti-ACSL4 (1:1000, 22401-1-AP, Proteintech), anti-TFRC (1:50, sc-393719, Santa Cruz Biotechnology), and anti-FTH1 (1:1000, ab183781, Abcam) overnight at 4 °C. The following day, the sections were incubated with the secondary antibody and stained with DAB. The sections were then treated with diluted hydrochloric acid and ammonia, routinely dehydrated, and resin mounted. Images were captured using an Olympus BH-2 microscope.

### Hematoxylin-eosin (HE) staining and histologic examination

A portion of the pancreas and spleen was fixed in 4% PFA solution (BL539A, Biosharp) and subsequently embedded in paraffin. Sections were cut to a thickness of 5 µm and placed on glass slides. HE-stained paraffin sections were subjected to xylene dewaxing, hydration in an ethanol gradient, staining with hematoxylin and eosin, dehydration in an alcohol gradient, followed by xylene treatment, and then mounted. Pancreatic tissue injury was observed under an Olympus BH-2 microscope. The scoring system described previously was applied [54] (details provided in Table S1).

### Serum AMY and LPS analysis

Rat blood samples were collected and left at room temperature for 30 min. Serum was isolated by centrifuging at 5000 × *g* for 10 min. Serum AMY and LPS levels were measured using standard assay kits (AMY: BC0615, LPS: BC2345; Solarbio), following the manufacturer’s instructions. Determined the absorbance by a Varioskan LUX multimode microplate reader (Thermo Fisher).

### Trypsin activity and iron assays

A 0.1 g sample of pancreatic tissue was homogenised on ice in 1 mL of extract fluid (BC2315 for Trypsin; Solarbio) and then centrifuged at 10,000 rpm for 10 min at 4 °C. A 10 μL supernatant was quickly added to 990 μL of working solution, and absorbance was measured at 253 nm at both 10 and 70 s using a Varioskan LUX multimode microplate reader (Thermo Fisher).

Cells (5 × 10^5^ per well) were cultured in a 6-well plate and treated under various conditions for 24 h. Cells were collected, and their iron levels were measured using a standard assay kit (ab83366, Abcam). Cells were homogenised with Iron Assay Buffer 10 times centrifuged at 16,000 rpm for 10 min and the supernatant was collected. After mixing with phenylmethylsulphonyl fluoride, 50 μL of each sample was combined with 50 μL of Iron Assay Buffer in a 96-well plate. For Fe^2+^ detection, 5 μL of Iron Buffer was added, and samples were incubated at 37 °C for 30 min. Subsequently, 100 μL of Iron Probe was added under dark conditions and incubated for 1 h. The absorbance was measured at 593 nm using a Varioskan LUX multimode microplate reader (Thermo Fisher).

### Lipid ROS assay

Lipid ROS was detected using a commercial kit (D3861, Invitrogen). Cells subjected to various treatments were cultured in 6-well tissue culture plates for 24 h, followed by a 2-h incubation with Component B (100 µM) at 37 °C. Subsequently, Component A (10 µM) was added and incubated for 30 min. Cells were washed three times with PBS. Absorbance was measured at 532 nm using a FACSCalibur flow cytometer (BD Biosciences). Data analysis was performed using FlowJo v10.8.1 software.

### ROS assay

Cells treated under various conditions were cultured in 6-well tissue culture plates for 24 h, then incubated with diacetyldichlorofluorescein diacetate (1:1000) (S0033M, Beyotime) at 37 °C for 20 min. Cells were washed three times with serum-free culture medium. Images were captured using a Leica DMI3000 B microscope. Mean DCFH-DA fluoresence intensity was measured using ImageJ 1.53e (National Institutes of Health, USA).

### FerroOrange assay

AR42J cells were seeded into 15 mm confocal dishes and incubated overnight at 37 °C. Cells were washed three times using a serum-free culture medium. Following various treatments, cells were washed again three times with serum-free culture medium, and incubated with 1 μmol/L FerroOrange (F374, DOJINGO) for 30 min at 37 °C in a 5% CO_2_ environment. Images were captured using the Leica DMI3000 B microscope. Mean FerroOrange fluorescence intensity was measured using ImageJ 1.53e (National Institutes of Health, USA).

### Prussian blue staining

Iron levels in pancreatic tissues were detected using a Prussian Blue Iron Stain Kit (G1422-1113, Solarbio). Tissue samples were sequentially treated with graded xylene and alcohol, washed three times in distilled water, and stained with Perls Stain (Solarbio) for 25 min. Samples were rinsed in distilled water, immersed in a solid red nuclear dye solution for 5 min, and rinsed again. The sections were then dehydrated and mounted with resin. Images were captured using an Olympus BH-2 microscope. Data analysis was performed using ImageJ 1.53e (National Institutes of Health, USA).

### Tissue MDA staining

Pancreatic tissue sections were sequentially treated with graded xylene and alcohol, followed by antigen retrieval in 0.01 M citrate buffer. After permeabilization with Triton X-100 for 15 min, sections were treated with an endogenous peroxidase blocker for 10 min, followed by a 30-min incubation with goat serum. Sections were then incubated overnight at 4 °C with FITC-conjugated anti-MDA antibody (1:100; ab27615; Abcam). The following day, sections were incubated with a secondary antibody, counterstained with DAPI, and mounted with an antifade solution (AR1109; Boster Biological Technology). Images were acquired using an Olympus FV3000 Laser Scanning Confocal Microscope.

### Chemicals

Ferrostatin-1 (S7243), Erastin (S7242) and Silibinin (NSC 651520) were obtained from Selleck (Houston, TX, USA). Deferoxamine (DFO, D9533) was obtained from Sigma-Aldrich. Cerulein (HY-A0190, MCE) was obtained from MedChemExpress.

### Statistical analysis

GraphPad Prism (GraphPad Inc.) software (version 26.0, IBM, USA) was used for statistical analysis. Student’s *t*-test was used to compare two groups, while one-way ANOVA was employed for comparisons involving more than two groups. Data were presented as mean ± standard deviation (SD), and *p* < 0.05 was considered statistically significant.

## Supplementary information

Supplementary Figures

Supplementary Table 1

Supplementary Table 2

Supplementary Table 3

Supplemental material-original figure

## Data Availability

The data supporting the findings of this study are available from the corresponding author upon reasonable request.
