# Supplementary material for: Mechanistic insights into the role of FAT10 in modulating NCOA4-mediated ferroptosis in pancreatic acinar cells during acute pancreatitis
Source: Cell Death Dis. 2025 May 15;16(1):385. doi: 10.1038/s41419-025-07715-9 (PMC12081885; doi:10.1038/s41419-025-07715-9)
Supplement: Supplementary file 1 — Supplementary Figures [file 41419_2025_7715_MOESM1_ESM.doc]

**
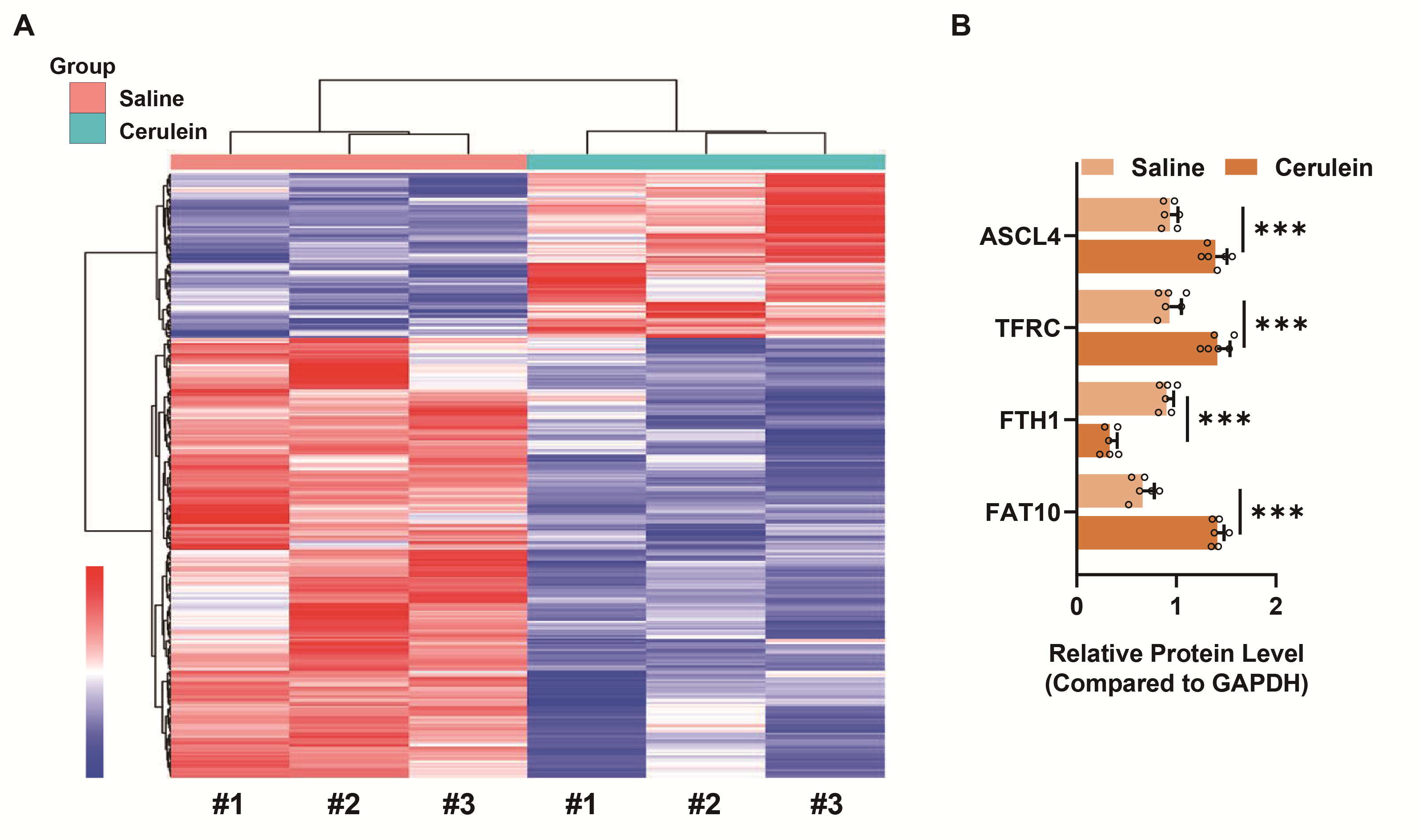
**

**Fig. S1 FAT10 expression and ferroptosis levels are increased in a rat model of AP induced by cerulein.** (A) Heatmap showed the expression level of distinguished proteins between control and AP groups. (B) The bar graph represented the quantification of relative protein expression levels of FAT10, FTH1, TFRC and ACSL4 expression in the pancreas of rats. ***p<0.001.

**
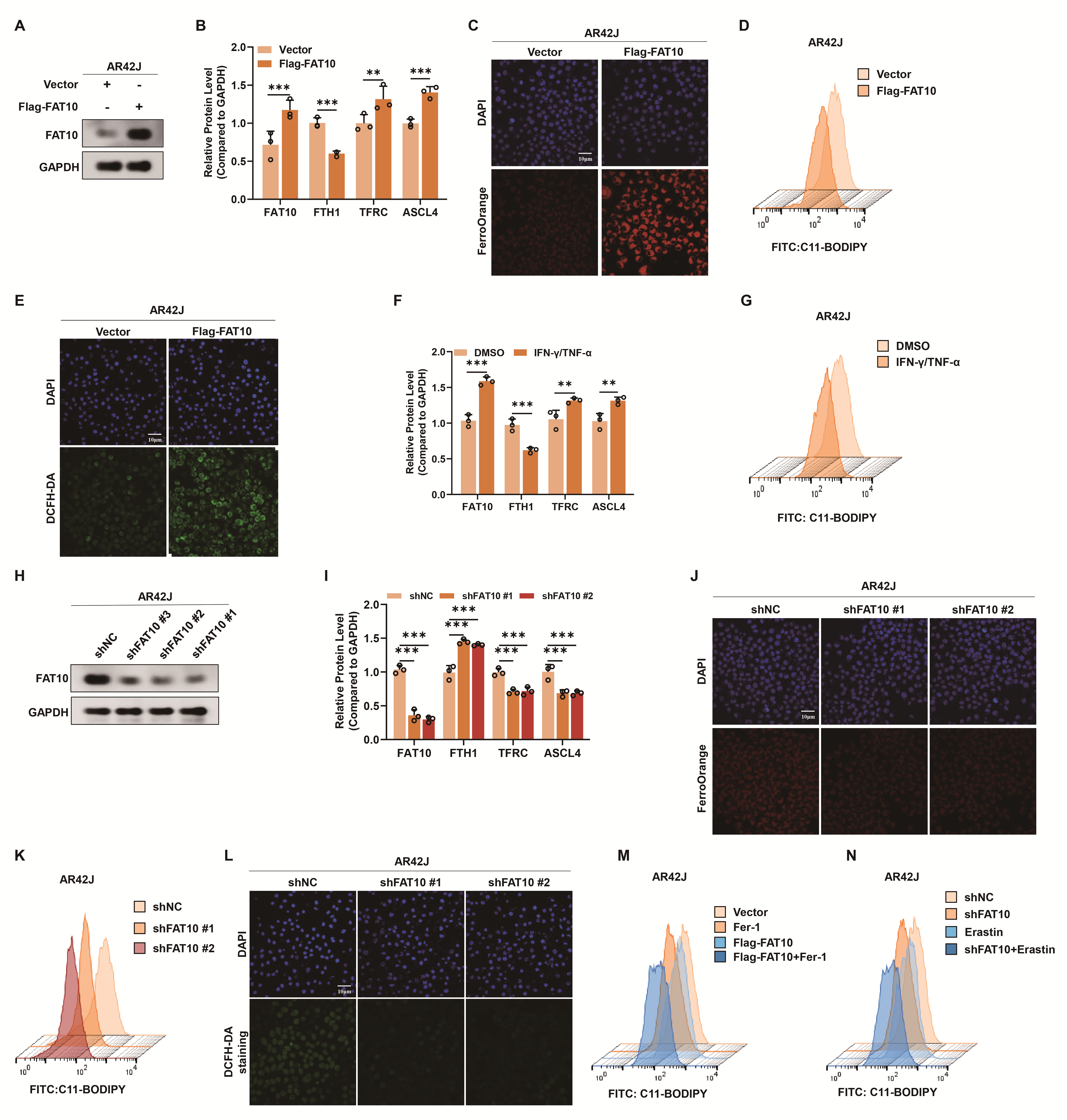
**

**Fig. S2 Overexpression of FAT10 enhances ferroptosis in pancreatic acinar cells.** (A) Evaluation of FAT10 overexpression efficiency in AR42J cells through Western blotting analysis. (B) The bar graph represented the quantification of relative protein expression levels of FAT10, FTH1, TFRC and ACSL4 expression in control and FAT10-stabled overexpressing AR42J cells. (C) FerroOrange staining of control and FAT10-stabled overexpressing AR42J cells (Scale bar: 10 μm). (D) Control and FAT10-stabled overexpressing AR42J cells were stained with C11-BODIPY and lipid ROS was assessed by flow cytometry. (E) DCFH-DA staining of control and FAT10-stabled overexpressing AR42J cells (Scale bar: 10 μm). (F) The bar graph represented the quantification of relative protein expression levels of FAT10, FTH1, TFRC and ACSL4 expression in DMSO and IFN-γ/TNF-α stimulated AR42J cells. (G) DMSO and IFN-γ/TNF-α stimulated AR42J cells were stained with C11-BODIPY and lipid ROS was assessed by flow cytometry. (H) Evaluation of shFAT10 efficiency in AR42J cells through western blotting analysis. (I) The bar graph represented the quantification of relative protein expression levels of FAT10, FTH1, TFRC and ACSL4 expression in shNC and shFAT10 transfected AR42J cells. (J) FerroOrange staining of shNC and shFAT10 transfected AR42J cells (Scale bar: 10 μm). (K) shNC and shFAT10 transfected AR42J cells were stained with C11-BODIPY and lipid ROS was assessed by flow cytometry. (L) DCFH-DA staining of shNC and shFAT10 transfected AR42J cells (Scale bar: 10 μm). (M) AR42J cells with the corresponding treatments were stained with C11-BODIPY and lipid ROS were assessed by flow cytometry. (N) AR42J cells with the corresponding treatments were stained with C11-BODIPY and lipid ROS was assessed by flow cytometry. **p<0.01; ***p<0.001.

**
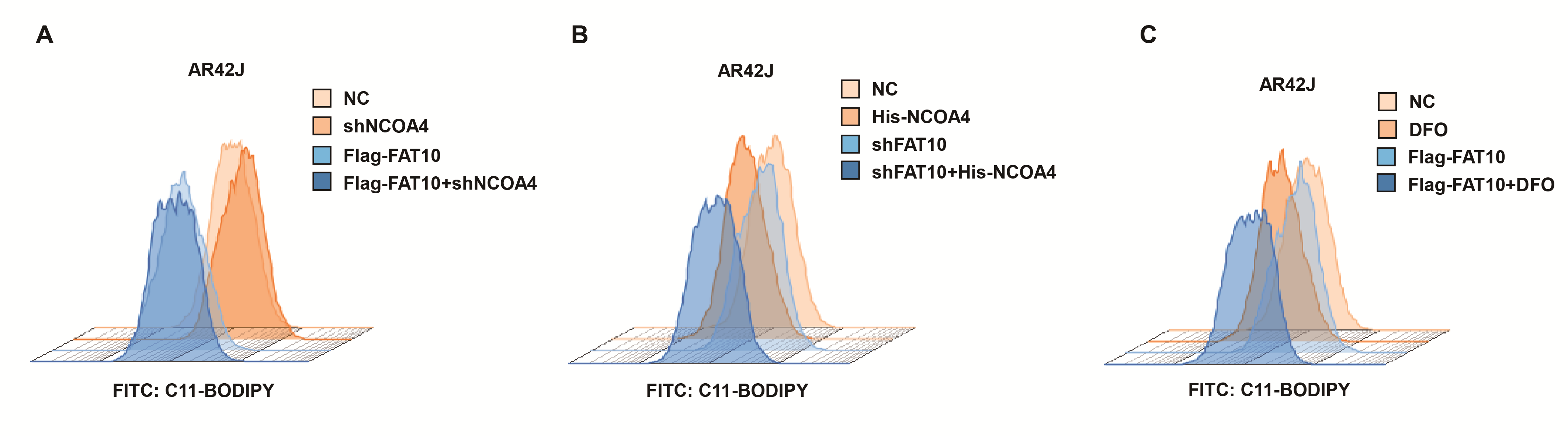
**

**Fig. S3 FAT10 regulates NCOA4 to promote lipid ROS in pancreatic anciar cells.** (A) Control and FAT10-stabled overexpressing AR42J cells, with or without NCOA4 knokdown were stained with C11-BODIPY and lipid ROS was assessed by flow cytometry. (B) shNC and shFAT10 transfected AR42J cells, with or without NCOA4 overexpression were stained with C11-BODIPY and lipid ROS was assessed by flow cytometry. (C) Control and FAT10-stabled overexpressing AR42J cells, with or without DFO treatment were stained with C11-BODIPY and lipid ROS was assessed by flow cytometry.


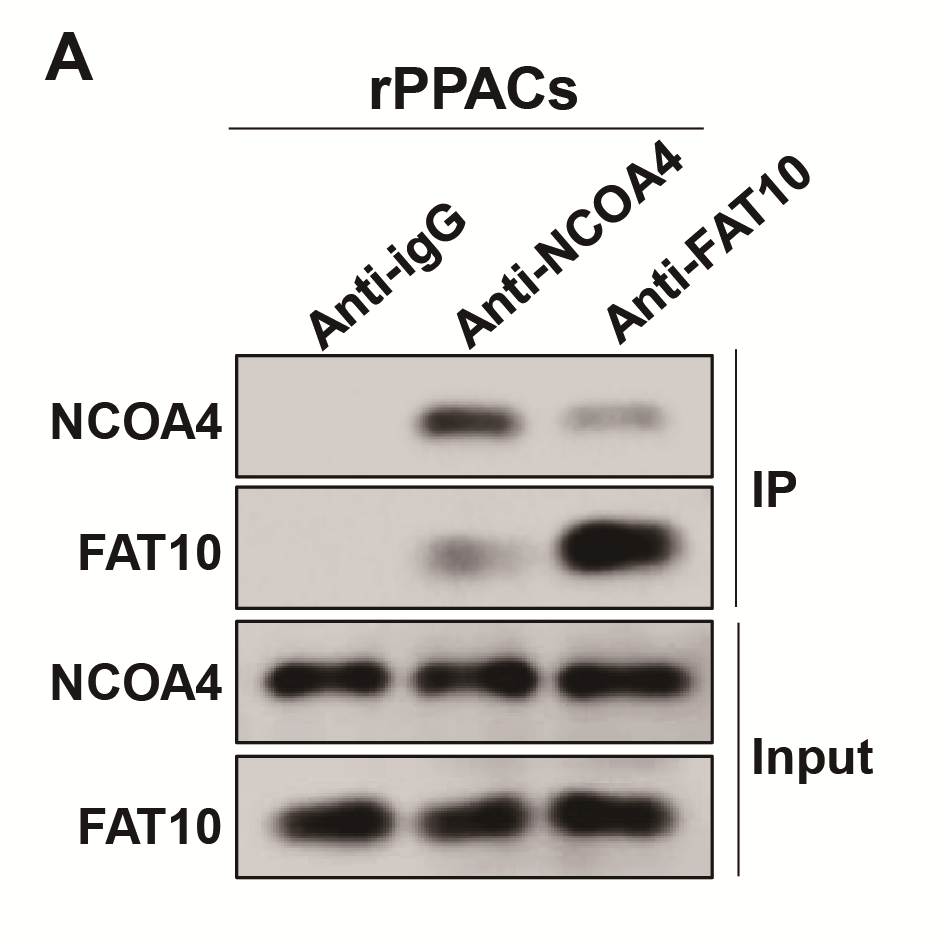


**Fig. S4** **NCOA4 bind to FAT10 in rPPACs.** (A) Co-IP for FAT10 and NCOA4 in rPPACs.


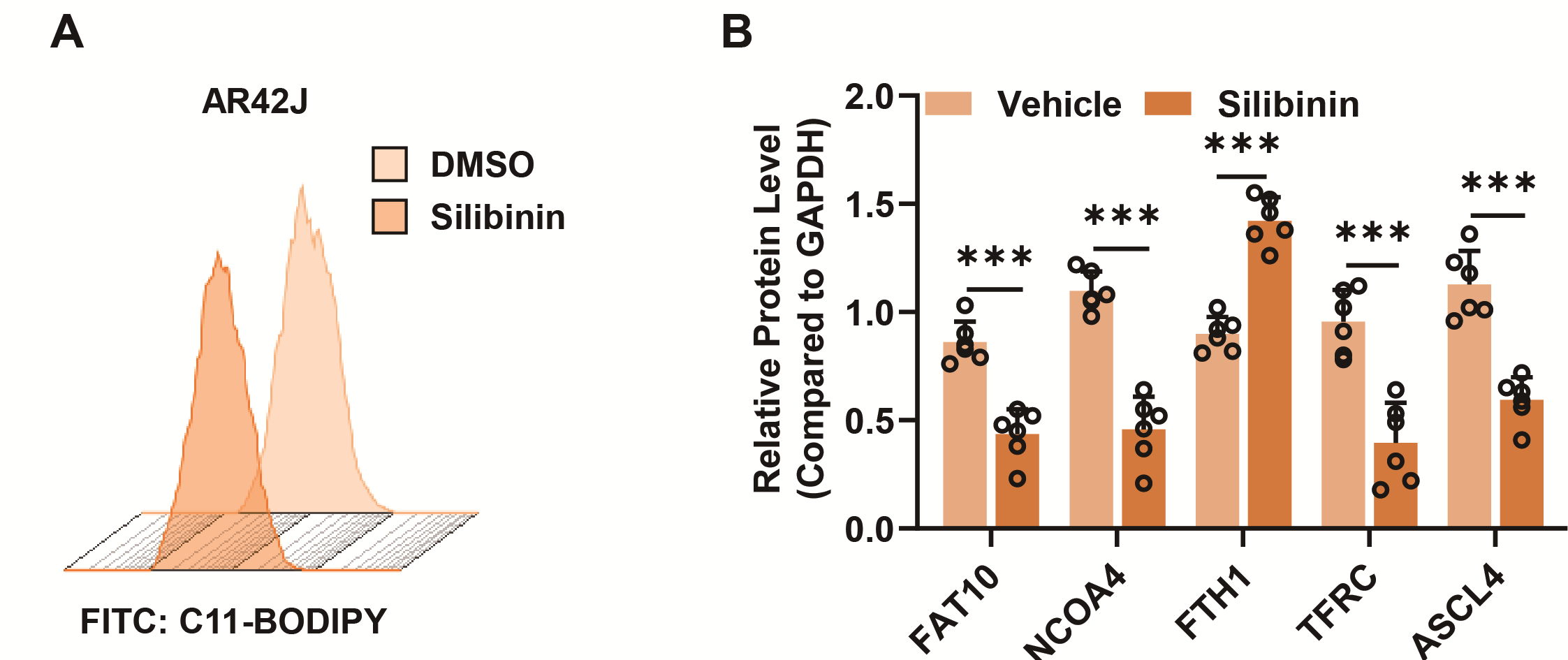


**Fig. S5 Silibinin inhibits the FAT10-NCOA4 axis to reduce ferroptosis *in vivo*.** (A) DMSO and Silibinin-treated AR42J cells were stained with C11-BODIPY and lipid ROS was assessed by flow cytometry. (B) The bar graph represented the quantification of relative protein expression levels of FAT10, NCOA4, FTH1, TFRC and ACSL4 expression in the rat pancreas. ***p<0.001.


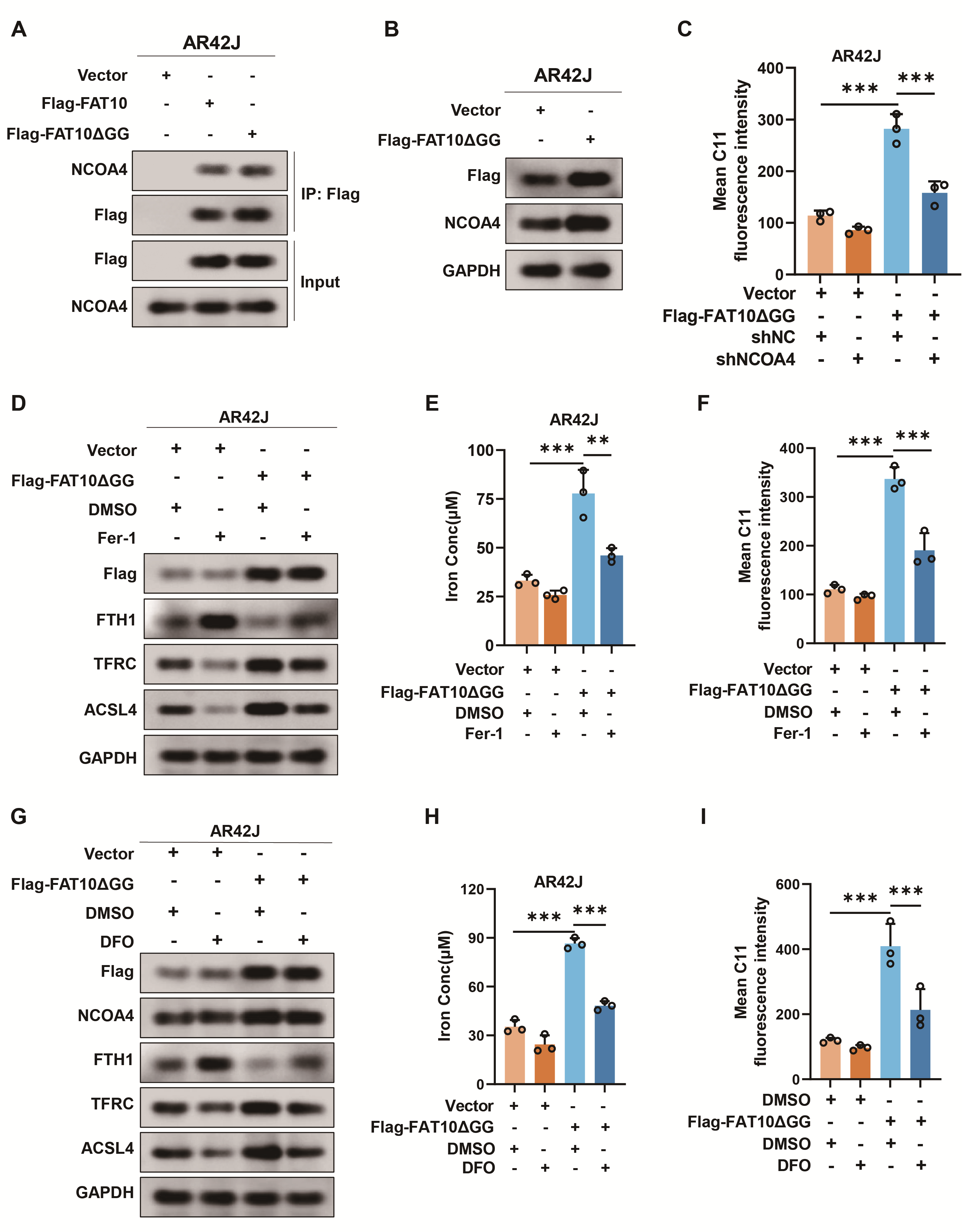


**Fig. S6 FAT10ΔGG regulates NCOA4 to promote ferroptosis in pancreatic anciar cells.**

(A) Co-IP for FAT10ΔGG and NCOA4 in AR42J cells. (B) Western blotting analysis of NCOA4 expression in control and FAT10ΔGG overexpressing AR42J cells. (C) Quantification of mean C11 fluorescence of control and FAT10ΔGG overexpressing AR42J cells, with or without NCOA4 knokdown. (D) Western blotting analysis of FAT10, FTH1, TFRC, ACSL4 expression in control and FAT10ΔGG overexpressing AR42J cells, with or without Fer-1 treatment. (E) Colorimetric analysis of Fe^2+^ levels in control and FAT10ΔGG overexpressing AR42J cells, with or without Fer-1 treatment. (F) Quantification of mean C11 fluorescence of control and FAT10ΔGG overexpressing AR42J cells, with or without Fer-1 treatment. (G) Western blotting analysis of FAT10, NCOA4, FTH1, TFRC, ACSL4 expression in control and FAT10ΔGG overexpressing AR42J cells, with or without DFO treatment. (H) Colorimetric analysis of Fe^2+^ levels in control and FAT10ΔGG overexpressing AR42J cells, with or without DFO treatment. (I) Quantification of mean C11 fluorescence of control and FAT10ΔGG overexpressing AR42J cells, with or without DFO treatment. **p<0.01; ***p<0.001.
