# Supplementary material for: Mechanistic insights into the role of FAT10 in modulating NCOA4-mediated ferroptosis in pancreatic acinar cells during acute pancreatitis
Source: Cell Death Dis. 2025 May 15;16(1):385. doi: 10.1038/s41419-025-07715-9 (PMC12081885; doi:10.1038/s41419-025-07715-9)
Supplement: Supplementary file 2 — Supplementary Table 1 [file 41419_2025_7715_MOESM2_ESM.docx]

**Supplementary Table 1**

**Table S1 The criteria of pancreatic histological score.**

|  | Histological score | Pathologic change |
| --- | --- | --- |
| Inflammation | 0 | None |
|  | 1 | Inflammatory cells present at interlobular areas |
|  | 2 | Present at intralobular areas |
|  | 3 | Present at interacini |
| Cell necrosis | 0 | None |
|  | 1 | <10% necrosis |
|  | 2 | <40% necrosis |
|  | 3 | >40%necrosis |
| Vacuolization | 0 | None |
|  | 1 | <20% acini with vacuoles |
|  | 2 | <50% acini |
|  | 3 | >50% acini |
| Acinar edema | 0 | None |
|  | 1 | Interlobular edema |
|  | 2 | Intralobular edema |
|  | 3 | Interacinar edema |
