# Supplementary material for: Mechanistic insights into the role of FAT10 in modulating NCOA4-mediated ferroptosis in pancreatic acinar cells during acute pancreatitis
Source: Cell Death Dis. 2025 May 15;16(1):385. doi: 10.1038/s41419-025-07715-9 (PMC12081885; doi:10.1038/s41419-025-07715-9)
Supplement: Supplementary file 3 — Supplementary Table 2 [file 41419_2025_7715_MOESM3_ESM.docx]

**Supplementary Table 2**

**Table S2 Primers and shRNA target sequences.**

| **Name** | **Sequences** |
| --- | --- |
| **Primers for quantitative real-time PCR** |  |
| FAT10 sense | 5’-GGAAAGAGGCTGGAAGATGGA-3’ |
| FAT10 antisense | 5’-GCGCTGTGAGAAAGAGCAAAC-3’ |
| NCOA4 sense | 5’-TTATGTGCTGGGTCTGGTAGC-3’ |
| NCOA4 antisense | 5’-TCATTCTGCTCACTGTTCCTTGA-3’ |
| GAPDH sense | 5’-ATGATTCTACCCACGGCAAG-3’ |
| GAPDH antisense | 5’-CTGGAAGATGGTGATGGGTT-3’ |
| **The target sites of shRNA** |  |
| shFAT10-1# | GGCCAUUAAUGACCUUUGA |
| shFAT10-2# | GAGCAUAUUAGGUCCCAAA |
| shFAT10-3# | CUAUCCACCUCACCCUAAA |
| shNCOA4-1# | CCAGGAAAGAAAGTGGGAAAC |
| shNCOA4-2# | AAGAAAGAAGGGAAGGACAAG |
| shNCOA4-3# | GCTGCAGTAGTAGAGAGACAC |
| shUbiquitin-1# | CCAAGATCCAAGATAAAGA |
| shUbiquitin-2# | GAGCCTTTCTTACGGCTAT |
| shUbiquitin-3# | GACGCAACACTCGTTGCAT |
| shRpn10-1# | CCTTATCACACTGGCTAAT |
| shRpn10-2# | CACGTTGAATGGCAAAGAT |
| shRpn10-3# | CCTGACCTAAGCAGTATGA |
