# Supplementary material for: Mechanistic insights into the role of FAT10 in modulating NCOA4-mediated ferroptosis in pancreatic acinar cells during acute pancreatitis
Source: Cell Death Dis. 2025 May 15;16(1):385. doi: 10.1038/s41419-025-07715-9 (PMC12081885; doi:10.1038/s41419-025-07715-9)
Supplement: Supplementary file 4 — Supplementary Table 3 [file 41419_2025_7715_MOESM4_ESM.docx]

**Supplementary Table 3 Data of Shotgun LC-MS/ MS analysis**

| Accession | Gene Name | Coverage [%] | # Peptides | # PSMs | # Unique Peptides | # AAs | MW [kDa] | calc. pI | Score Mascot:Mascot |
| --- | --- | --- | --- | --- | --- | --- | --- | --- | --- |
| Q6T487 | Actn1 | 6 | 5 | 6 | 3 | 914 | 105.4 | 5.39 | 83 |
| A0A0G2JXC3 | Rps21 | 19 | 2 | 2 | 2 | 127 | 14.1 | 9.32 | 93 |
| P15178 | Dars1 | 2 | 1 | 1 | 1 | 501 | 57.1 | 6.44 | 57 |
| A0A8I6ANE0 | Dpysl2 | 8 | 4 | 5 | 4 | 673 | 73.1 | 6.4 | 228 |
| A0A8L2R9H0 | Rps8 | 13 | 3 | 3 | 3 | 209 | 24.3 | 10.32 | 97 |
| P40329 | Rars1 | 2 | 1 | 1 | 1 | 660 | 75.8 | 7.05 | 19 |
| Q4V7D7 | Rbm22 | 2 | 1 | 1 | 1 | 420 | 46.8 | 8.54 | 29 |
| A0A0G2JU96 | Ahnak | 3 | 3 | 4 | 3 | 5322 | 568.5 | 6.24 | 109 |
| A0A8I6GCL3 | Cald1 | 2 | 2 | 2 | 2 | 750 | 86.9 | 5.43 | 68 |
| A6HIL4 | rCG_33529 | 4 | 1 | 1 | 1 | 158 | 18.1 | 9.77 | 43 |
| A0A8I6ANV9 | Anxa2 | 11 | 4 | 4 | 4 | 383 | 43.2 | 8.05 | 131 |
| A6HYK1 | Mrpl21_predicted | 4 | 1 | 1 | 1 | 209 | 23.4 | 9.95 | 66 |
| P09495 | Tpm4 | 19 | 6 | 6 | 4 | 248 | 28.5 | 4.69 | 167 |
| A0A8I6GJN3 | Myo1d | 1 | 1 | 1 | 1 | 1020 | 117.1 | 9.47 | 28 |
| P05942 | S100a4 | 8 | 1 | 1 | 1 | 101 | 11.8 | 5.12 | 46 |
| G3V6W6 | Psmc6 | 3 | 1 | 1 | 1 | 403 | 45.8 | 7.78 | 58 |
| F1LQC8 | Cdk7 | 3 | 1 | 1 | 1 | 347 | 39.2 | 9.16 | 52 |
| F1LS40 | Col1a2 | 1 | 1 | 1 | 1 | 1372 | 129.5 | 9.29 | 43 |
| A0A8L2QPP4 | Capza1 | 19 | 3 | 3 | 3 | 289 | 33 | 7.18 | 77 |
| A0A8I6AM85 | Rpl18a | 4 | 1 | 1 | 1 | 235 | 26.3 | 9.94 | 31 |
| P04961 | Pcna | 5 | 1 | 1 | 1 | 261 | 28.7 | 4.69 | 75 |
| A6KRG3 | Acaa2 | 2 | 1 | 1 | 1 | 397 | 41.9 | 8.15 | 41 |
| A6HBN3 | Mbip_predicted | 3 | 1 | 1 | 1 | 342 | 38.4 | 7.49 | 44 |
| A6HME9 | rCG_27231 | 15 | 6 | 6 | 5 | 357 | 37.1 | 8.31 | 189 |
| Q71UF4 | Rbbp7 | 5 | 2 | 3 | 2 | 425 | 47.8 | 5.05 | 116 |
| F1LP05 | Atp5f1a | 17 | 9 | 10 | 9 | 553 | 59.8 | 9.26 | 413 |
| A0A8I5ZUU5 | Ergic1 | 3 | 1 | 1 | 1 | 313 | 35 | 7.27 | 39 |
| A0A8I5ZYH2 | Hba-a1 | 6 | 1 | 1 | 1 | 199 | 21.9 | 10.15 | 50 |
| P62630 | Eef1a1 | 5 | 3 | 3 | 3 | 462 | 66 | 6.74 | 373 |
| F7FLB2 | Pgm2 | 2 | 1 | 1 | 1 | 666 | 73.6 | 6.65 | 42 |
| A0A8I6GAR6 | Rpl4 | 11 | 4 | 8 | 4 | 421 | 47.3 | 11.02 | 238 |
| D3ZZW6 | Hils1 | 5 | 1 | 1 | 1 | 169 | 18.6 | 12.15 | 38 |
| A0A8L2UJN1 | Mybbp1a | 1 | 1 | 1 | 1 | 1348 | 152.7 | 8.95 | 55 |
| A0A8I6AD55 | Chchd3 | 8 | 2 | 2 | 2 | 232 | 27 | 7.42 | 79 |
| D4A7I6 | Washc4 | 14 | 16 | 17 | 16 | 1172 | 135.9 | 7.46 | 492 |
| D3ZM33 | LOC100362298 | 31 | 6 | 7 | 6 | 152 | 17.8 | 11.11 | 293 |
| F1LRJ9 | Selenbp1 | 3 | 1 | 1 | 1 | 496 | 55.1 | 6.86 | 30 |
| M0R9Q1 | Rbm14 | 5 | 3 | 3 | 3 | 669 | 69.5 | 9.67 | 110 |
| Q3KRE0 | Atad3 | 3 | 2 | 2 | 2 | 591 | 66.7 | 9.29 | 73 |
| E9PT29 | Ddx17 | 12 | 7 | 8 | 1 | 652 | 72.6 | 8.68 | 326 |
| A0A8I5ZNX8 | Rpsa | 4 | 1 | 1 | 1 | 331 | 36.6 | 6.43 | 59 |
| M0RD03 | Eif4g1 | 1 | 1 | 1 | 1 | 1641 | 180.1 | 5.33 | 22 |
| Q7TP54 | Ripor2 | 1 | 1 | 1 | 1 | 1310 | 144.6 | 5.47 | 87 |
| F1LT35 | RGD1564606 | 18 | 4 | 4 | 4 | 171 | 19.5 | 10.48 | 120 |
| G3V7L6 | Psmc2 | 3 | 1 | 1 | 1 | 433 | 48.6 | 5.95 | 22 |
| F1LM66 | Eftud2 | 1 | 1 | 1 | 1 | 972 | 109.4 | 5 | 31 |
| M0R5J4 | Eno1-ps1 | 26 | 9 | 11 | 9 | 434 | 47 | 6.37 | 365 |
| A0A8I6GIA0 | Rps13 | 17 | 4 | 4 | 4 | 177 | 20 | 10.18 | 126 |
| A0A8I5ZJK0 | Arf4 | 10 | 2 | 3 | 1 | 186 | 20.8 | 8.32 | 133 |
| A0A0G2K654 | H1f2 | 29 | 8 | 8 | 5 | 212 | 21.3 | 11 | 278 |
| A0A8I6A8L3 | Dnpep | 3 | 1 | 1 | 1 | 480 | 53 | 7.01 | 31 |
| A6JP10 | Pcmt1 | 9 | 2 | 2 | 2 | 286 | 30.4 | 7.28 | 68 |
| A6IXE0 | Tcof1_predicted | 1 | 1 | 1 | 1 | 1395 | 144.6 | 9.16 | 51 |
| A0A8I6A1H0 | AABR07060610.1 | 23 | 3 | 3 | 3 | 194 | 21 | 9.76 | 90 |
| A0A8I6G4V0 | Rpl10l1 | 8 | 2 | 3 | 2 | 214 | 24.5 | 10.11 | 82 |
| F1M853 | Rrbp1 | 2 | 3 | 3 | 3 | 1453 | 157.4 | 9.07 | 92 |
| F7F271 | Sec61b | 10 | 1 | 1 | 1 | 96 | 10 | 11.56 | 67 |
| G3V6I9 | Rpl26 | 9 | 2 | 2 | 2 | 210 | 24.4 | 10.3 | 94 |
| A0A8I5ZP83 | Chmp4b | 32 | 7 | 7 | 7 | 224 | 24.9 | 4.82 | 179 |
| A0A0G2K3Z9 | Prdx1l1 | 25 | 5 | 5 | 5 | 199 | 22.2 | 8.4 | 135 |
| Q5M819 | Psph | 3 | 1 | 1 | 1 | 225 | 25 | 5.67 | 45 |
| A0A8L2QTU4 | Nexn | 2 | 1 | 1 | 1 | 670 | 79.8 | 5.27 | 55 |
| D3ZGE6 | Cttn | 1 | 1 | 1 | 1 | 557 | 62.2 | 5.35 | 43 |
| A0A8I6B017 | Rps17 | 15 | 2 | 2 | 2 | 209 | 23.2 | 9.74 | 39 |
| A0A0G2JWU1 | LOC500959 | 17 | 4 | 4 | 4 | 253 | 27.4 | 6.54 | 181 |
| A0A8I6ALC1 | Hnrnpa1 | 22 | 6 | 6 | 4 | 373 | 38.8 | 9.13 | 229 |
| D3ZX01 | Rps4y2 | 10 | 3 | 3 | 3 | 304 | 33.5 | 10.07 | 88 |
| D3ZE63 | LOC679748 | 10 | 1 | 1 | 1 | 115 | 12.6 | 7.9 | 36 |
| A0A8L2QDI1 | Hspa5 | 31 | 18 | 21 | 17 | 655 | 72.4 | 5.2 | 745 |
| A0A0G2K2P5 | Tjp1 | 8 | 12 | 12 | 12 | 1765 | 197 | 6.6 | 430 |
| A0A8I6ASU0 | ENSRNOG00000066549 | 12 | 3 | 3 | 3 | 225 | 25.2 | 8.46 | 86 |
| A0A8L2UJE8 | Rpl35 | 15 | 2 | 2 | 2 | 124 | 14.7 | 11.05 | 42 |
| A0A8I6A2Z2 | Fasn | 1 | 2 | 2 | 2 | 2521 | 274.4 | 6.44 | 53 |
| A0A8I6GGC8 | Lgals1 | 8 | 1 | 1 | 1 | 146 | 16.1 | 5.14 | 59 |
| A0A8I6A9D8 | Dek | 2 | 1 | 1 | 1 | 390 | 44.2 | 6.51 | 0 |
| Q6P3E1 | Rps16 | 30 | 5 | 5 | 5 | 161 | 18 | 10.2 | 114 |
| A0A8I6ASB9 | Myo1c | 1 | 1 | 1 | 1 | 1063 | 121.8 | 9.35 | 43 |
| E9PTI6 | Raly | 15 | 6 | 6 | 6 | 381 | 39.8 | 10.14 | 198 |
| A6IAC1 | rCG_56483 | 4 | 1 | 1 | 1 | 423 | 46.6 | 6.6 | 15 |
| A0A8I5ZR22 | Krt79 | 6 | 4 | 5 | 1 | 528 | 57.3 | 7.75 | 178 |
| G3V6H2 | Prpf8 | 4 | 10 | 11 | 10 | 2335 | 273.4 | 8.84 | 271 |
| A0A8I6AAZ0 | Ruvbl2 | 23 | 11 | 12 | 11 | 476 | 52.4 | 6.39 | 371 |
| P62329 | Tmsb4x | 25 | 1 | 1 | 1 | 44 | 5.1 | 5.06 | 35 |
| P28480 | Tcp1 | 3 | 2 | 2 | 2 | 556 | 60.3 | 6.21 | 70 |
| A6HK76 | rCG_63505 | 4 | 1 | 1 | 1 | 233 | 26.8 | 9.22 | 63 |
| A0A8I5ZSP3 | Ywhae | 10 | 3 | 4 | 2 | 262 | 29.7 | 4.89 | 107 |
| A0A8L2QUM7 | Cfl1 | 5 | 1 | 1 | 1 | 213 | 23 | 8.07 | 58 |
| A6KEZ2 | Tpm1 | 33 | 10 | 10 | 3 | 248 | 28.5 | 4.77 | 343 |
| A0A8I6AIB7 | Eif3k | 5 | 1 | 1 | 1 | 264 | 30.1 | 5.1 | 25 |
| P60123 | Ruvbl1 | 11 | 4 | 4 | 4 | 456 | 50.2 | 6.42 | 138 |
| A0A8L2PZP1 | Tra2b | 3 | 1 | 1 | 1 | 308 | 36.4 | 10.56 | 49 |
| A6KCR6 | Kb9 | 6 | 4 | 4 | 1 | 585 | 62.1 | 8.48 | 123 |
| A6HWZ2 | rCG_47496 | 2 | 1 | 1 | 1 | 305 | 31.8 | 6.57 | 47 |
| A0A8I6AHN0 | LOC103690796 | 5 | 1 | 1 | 1 | 193 | 21.8 | 8.54 | 53 |
| A0A8I6GF99 | Tmpo | 5 | 3 | 3 | 3 | 716 | 79.4 | 9.33 | 117 |
| A0A8I6AK10 | Fscn1 | 4 | 2 | 2 | 2 | 493 | 54.5 | 6.89 | 77 |
| A6JHP5 | Usmg5 | 12 | 1 | 1 | 1 | 85 | 9.7 | 9.94 | 50 |
| M0R757 | LOC100360413 | 19 | 7 | 7 | 7 | 462 | 50.1 | 9.01 | 220 |
| A6JH60 | Pycs_predicted | 2 | 2 | 2 | 2 | 795 | 87.3 | 7.56 | 68 |
| A6HVR9 | Rpl34 | 15 | 2 | 2 | 2 | 117 | 13.3 | 11.47 | 67 |
| Q66HF1 | Ndufs1 | 1 | 1 | 1 | 1 | 727 | 79.4 | 5.9 | 39 |
| A0A0G2K3K2 | Actb | 40 | 13 | 24 | 5 | 378 | 42.1 | 5.48 | 662 |
| A0A8L2Q453 | Eps8 | 3 | 2 | 2 | 2 | 825 | 92.2 | 8.1 | 41 |
| A0A8I6GB84 | Vdac2 | 7 | 2 | 2 | 2 | 303 | 32.4 | 7.25 | 81 |
| Q641Y8 | Ddx1 | 3 | 2 | 3 | 2 | 740 | 82.4 | 7.23 | 51 |
| P15791 | Camk2d | 2 | 1 | 1 | 1 | 533 | 60 | 7.27 | 21 |
| A0A8I5ZQ38 | Hnrnpm | 17 | 12 | 14 | 1 | 679 | 72 | 8.12 | 480 |
| A0A8I6A8A1 | Tubg2 | 2 | 1 | 1 | 1 | 477 | 53.8 | 8.57 | 34 |
| A0A8I5Y1J2 | ENSRNOG00000067128 | 12 | 2 | 2 | 2 | 154 | 16.8 | 7.81 | 106 |
| P43278 | H1-0 | 9 | 2 | 2 | 2 | 194 | 20.9 | 10.9 | 85 |
| D4ACW1 | Nop2 | 1 | 1 | 1 | 1 | 776 | 85.3 | 9.31 | 40 |
| A0A0G2JWB2 | Rpl11 | 10 | 2 | 2 | 2 | 178 | 20.3 | 9.6 | 82 |
| A0A8I6ACY4 | Rps12 | 5 | 1 | 1 | 1 | 148 | 16 | 8.53 | 37 |
| A0A8I6AHY5 | Eppk1 | 6 | 13 | 14 | 13 | 4284 | 472.4 | 5.96 | 343 |
| P81795 | Eif2s3 | 10 | 4 | 4 | 4 | 472 | 51 | 8.4 | 158 |
| A0A8I6AVL2 | Acad10 | 4 | 6 | 6 | 6 | 1492 | 165.3 | 7.9 | 237 |
| P67779 | Phb1 | 4 | 1 | 1 | 1 | 272 | 29.8 | 5.76 | 49 |
| A6I6H7 | rCG_40478 | 8 | 2 | 2 | 2 | 277 | 30.6 | 9.48 | 96 |
| F1LV01 | Wdr19 | 2 | 1 | 1 | 1 | 1341 | 151.4 | 6.52 | 0 |
| B0BMW0 | Rab14 | 8 | 2 | 2 | 2 | 215 | 23.9 | 6.21 | 81 |
| A6KLF5 | Them2_predicted | 7 | 1 | 1 | 1 | 140 | 15.3 | 8.84 | 37 |
| A0A8L2QIW0 | Rpl6 | 7 | 2 | 2 | 2 | 303 | 34.1 | 10.7 | 63 |
| Q6IFU9 | Krt16 | 11 | 7 | 14 | 3 | 463 | 50.7 | 5.12 | 519 |
| A0A8I5Y4N3 | Alb | 7 | 3 | 5 | 3 | 616 | 69.6 | 6.3 | 166 |
| Q27W01 | Rbm8a | 5 | 1 | 1 | 1 | 174 | 19.9 | 5.72 | 52 |
| P84092 | Ap2m1 | 4 | 2 | 2 | 2 | 435 | 49.6 | 9.54 | 74 |
| A6HZV0 | RGD1562784_predicted | 5 | 4 | 4 | 4 | 758 | 86.1 | 4.87 | 114 |
| Q64428 | Hadha | 4 | 4 | 4 | 4 | 763 | 82.6 | 9.06 | 113 |
| Q6IFU7 | Krt42 | 19 | 9 | 11 | 3 | 452 | 50.2 | 5.16 | 346 |
| D3ZY51 | Pkp1 | 1 | 1 | 1 | 1 | 728 | 80.9 | 8.91 | 61 |
| F1LSS1 | Smc1a | 1 | 1 | 1 | 1 | 1233 | 143.1 | 7.64 | 40 |
| A0A8I5Y747 | Ncl | 10 | 7 | 7 | 4 | 748 | 81 | 4.78 | 257 |
| A0A8I6A0G6 | Krt33a | 2 | 1 | 1 | 1 | 411 | 46.5 | 4.84 | 52 |
| A0A8I6A6H8 | Serpinb6a | 2 | 1 | 1 | 1 | 400 | 45.1 | 6.47 | 46 |
| A0A8I6AIH6 | Suclg2 | 2 | 1 | 1 | 1 | 438 | 47.7 | 8.12 | 34 |
| A0A8I6A2P8 | Akr1b1 | 2 | 1 | 1 | 1 | 321 | 35.8 | 7.05 | 58 |
| A0A0G2KA90 | Dsc1 | 1 | 1 | 1 | 1 | 896 | 100 | 5.59 | 51 |
| A6I8F8 | rCG_40707 | 6 | 1 | 2 | 1 | 148 | 16.9 | 10.61 | 95 |
| A0A0G2JSR7 | Matr3 | 12 | 10 | 10 | 10 | 845 | 94.4 | 6.25 | 352 |
| A7VJC2 | Hnrnpa2b1 | 42 | 12 | 14 | 10 | 353 | 37.5 | 8.95 | 372 |
| Q6IG05 | Krt75 | 12 | 7 | 8 | 1 | 542 | 59 | 8.07 | 271 |
| A0A8I6A0A3 | Gsr | 2 | 1 | 1 | 1 | 502 | 53.7 | 8.03 | 18 |
| A0A8I6GBX4 | Dld | 2 | 1 | 1 | 1 | 538 | 57.4 | 7.01 | 38 |
| A0A0G2K1C0 | Actr3 | 23 | 8 | 8 | 8 | 419 | 47.6 | 6.18 | 241 |
| A6IJ31 | rCG_55135 | 1 | 1 | 1 | 1 | 2577 | 273.5 | 6.32 | 27 |
| A0A8L2QVY8 | Rpl3 | 11 | 3 | 3 | 3 | 419 | 47.7 | 10.2 | 92 |
| G3V9N0 | Pabpc4 | 3 | 2 | 2 | 1 | 660 | 72.4 | 9.35 | 71 |
| A1A5L2 | Pgm1 | 4 | 2 | 2 | 2 | 583 | 63.3 | 6.4 | 41 |
| A0A8I5ZMM2 | Cad | 1 | 2 | 2 | 2 | 2321 | 253.2 | 6.7 | 65 |
| A0A8I5ZKR8 | Diras2 | 6 | 1 | 1 | 1 | 199 | 22.5 | 8.76 | 55 |
| P62755 | Rps6 | 12 | 3 | 3 | 3 | 249 | 28.7 | 10.84 | 119 |
| G3V7T6 | Sf3b1 | 1 | 1 | 1 | 1 | 1304 | 145.7 | 7.09 | 54 |
| F7F7A6 | Eif4a1 | 12 | 4 | 4 | 3 | 411 | 46.8 | 5.68 | 165 |
| Q91V33 | Khdrbs1 | 4 | 3 | 3 | 3 | 443 | 48.3 | 8.72 | 67 |
| A0A9K3Y6T2 | Snrpa | 2 | 1 | 1 | 1 | 281 | 31.2 | 9.83 | 40 |
| A6I7B6 | Hbb-b2 | 16 | 2 | 2 | 2 | 147 | 16 | 8.07 | 43 |
| A0A8L2QF86 | Prpf19 | 3 | 2 | 2 | 2 | 523 | 57.3 | 6.61 | 82 |
| A0A8L2UK98 | Fbl | 3 | 1 | 1 | 1 | 330 | 34.5 | 10.24 | 31 |
| G3V8U9 | Psmb4 | 3 | 1 | 1 | 1 | 339 | 37.2 | 8.15 | 51 |
| A0A0G2K4X8 | Skp1 | 7 | 1 | 1 | 1 | 165 | 18.9 | 4.54 | 75 |
| A0A0G2K8V2 | Vcl | 1 | 1 | 1 | 1 | 1134 | 123.6 | 5.71 | 43 |
| D3ZQM0 | Sf3a1 | 1 | 1 | 1 | 1 | 791 | 88.5 | 5.22 | 43 |
| A0A8I5ZUG8 | Psmc3 | 2 | 1 | 1 | 1 | 449 | 50.2 | 5.39 | 50 |
| Q6AYK8 | Eif3d | 5 | 3 | 3 | 3 | 548 | 63.9 | 6.05 | 79 |
| F7ELS2 | Cct6a | 3 | 2 | 2 | 2 | 563 | 61.7 | 7.08 | 98 |
| B1WC67 | Slc25a24 | 2 | 1 | 1 | 1 | 475 | 52.9 | 8.05 | 55 |
| P62138 | Ppp1ca | 6 | 2 | 2 | 2 | 330 | 37.5 | 6.33 | 88 |
| A0A8I6AST2 | Gsn | 3 | 2 | 2 | 2 | 795 | 87.6 | 5.99 | 58 |
| P62845 | Rps15 | 8 | 1 | 1 | 1 | 145 | 17 | 10.39 | 0 |
| A0A0G2JZE6 | Rack1 | 8 | 3 | 3 | 3 | 323 | 35.5 | 7.91 | 95 |
| A0A0G2JSH9 | Prdx2 | 5 | 1 | 1 | 1 | 198 | 21.8 | 5.59 | 32 |
| P62832 | Rpl23 | 7 | 1 | 1 | 1 | 140 | 14.9 | 10.51 | 41 |
| A6ITL0 | Capzb | 16 | 5 | 6 | 5 | 277 | 31.3 | 5.74 | 285 |
| D3ZM39 | Dsg1 | 2 | 2 | 2 | 2 | 1060 | 113.8 | 4.86 | 80 |
| A0A8I5Y1A4 | Cycs | 6 | 1 | 1 | 1 | 140 | 15.5 | 9.39 | 29 |
| A0A8L2QEL3 | Hnrnpl | 3 | 2 | 2 | 2 | 630 | 68.5 | 8.56 | 44 |
| A0A8I6AI09 | Rpl31 | 4 | 1 | 1 | 1 | 233 | 26.5 | 9.64 | 53 |
| G3V727 | Ddx47 | 3 | 1 | 1 | 1 | 457 | 51 | 8.85 | 57 |
| A0A0G2JSI1 | Aldh9a1 | 2 | 1 | 2 | 1 | 521 | 56.4 | 7.18 | 90 |
| A0A8I5ZNK4 | ENSRNOG00000062895 | 9 | 4 | 6 | 4 | 494 | 53.5 | 10.35 | 202 |
| Q5RKG9 | Eif4b | 3 | 1 | 1 | 1 | 611 | 69 | 5.81 | 64 |
| A6HBA3 | RGD1565406_predicted | 7 | 1 | 1 | 1 | 165 | 19.1 | 6.52 | 31 |
| Q6AYR1 | Tfg | 5 | 2 | 2 | 2 | 398 | 43.1 | 5.1 | 81 |
| A0A8I5ZP07 | Gnb2 | 3 | 1 | 1 | 1 | 382 | 41.4 | 6.6 | 42 |
| Q5RKI5 | Flii | 1 | 1 | 1 | 1 | 1270 | 144.8 | 6 | 44 |
| Q66HD0 | Hsp90b1 | 11 | 8 | 8 | 7 | 804 | 92.7 | 4.81 | 281 |
| Q3B8Q2 | Eif4a3 | 26 | 10 | 11 | 9 | 411 | 46.8 | 6.73 | 323 |
| A0A096MKE5 | Zfp326 | 2 | 1 | 1 | 1 | 581 | 65.2 | 5.12 | 54 |
| D4A6W6 | RGD1561333 | 11 | 2 | 2 | 2 | 257 | 28.1 | 10.78 | 78 |
| A0A8I6AKU4 | Ddx39b | 6 | 3 | 3 | 3 | 456 | 52.3 | 5.68 | 119 |
| A0A8I6ATX8 | Washc3 | 9 | 2 | 2 | 2 | 231 | 25.6 | 5.34 | 72 |
| A6KEG3 | Hnrpc | 26 | 9 | 9 | 9 | 313 | 34.4 | 5.05 | 256 |
| A6HE59 | Hnrnpab | 10 | 3 | 4 | 2 | 332 | 36.2 | 6.95 | 116 |
| A0A0H2UHK2 | Pgrmc1 | 4 | 1 | 1 | 1 | 222 | 24.7 | 4.5 | 45 |
| A0A8L2Q875 | Ckb | 3 | 1 | 1 | 1 | 383 | 43 | 5.58 | 68 |
| A6HBR2 | LOC368070 | 1 | 1 | 1 | 1 | 729 | 82.6 | 7.4 | 70 |
| A0A8I5ZTC3 | Dapk3 | 2 | 1 | 1 | 1 | 517 | 58.8 | 8.25 | 0 |
| B3GNI6 | Septin11 | 2 | 1 | 1 | 1 | 431 | 49.7 | 6.68 | 36 |
| A0A8I6G7P2 | Rps27l | 7 | 1 | 1 | 1 | 112 | 12.5 | 9.57 | 44 |
| A0A8I6GKL5 | Rpl35a | 12 | 2 | 2 | 2 | 135 | 15.2 | 10.26 | 62 |
| A0A0G2KAZ7 | Hnrnpdl | 4 | 2 | 3 | 1 | 419 | 46.3 | 9.52 | 111 |
| P62282 | Rps11 | 11 | 2 | 2 | 2 | 158 | 18.4 | 10.3 | 82 |
| A0A8I6GLE7 | Cct2 | 1 | 1 | 1 | 1 | 546 | 58.5 | 6.49 | 41 |
| Q5D059 | Hnrnpk | 16 | 6 | 7 | 6 | 464 | 51 | 5.33 | 256 |
| A0A8L2RAD9 | Tuba1b | 11 | 5 | 5 | 2 | 475 | 52.6 | 5.49 | 141 |
| Q6P6Q2 | Krt5 | 24 | 16 | 17 | 8 | 576 | 61.8 | 7.8 | 517 |
| D4A2C6 | Lsm4 | 5 | 1 | 1 | 1 | 138 | 15.2 | 10.05 | 42 |
| A0A8I6A2X3 | Myo18a | 2 | 5 | 5 | 5 | 2085 | 236.1 | 6.1 | 158 |
| P25113 | Pgam1 | 4 | 1 | 1 | 1 | 254 | 28.8 | 7.18 | 52 |
| F1LNJ2 | Snrnp200 | 2 | 5 | 5 | 5 | 2139 | 244.7 | 6.09 | 130 |
| D3Z9L0 | Agk | 2 | 1 | 1 | 1 | 490 | 54.2 | 8.6 | 33 |
| A6KMN8 | rCG_40950 | 3 | 1 | 1 | 1 | 306 | 31.6 | 7.91 | 52 |
| P09527 | Rab7a | 5 | 1 | 1 | 1 | 207 | 23.5 | 6.7 | 65 |
| F1LS79 | Cspg4 | 5 | 9 | 9 | 9 | 2326 | 251.9 | 5.48 | 320 |
| A0A8L2RAI7 | Zfr | 1 | 1 | 1 | 1 | 1082 | 117.7 | 9.01 | 34 |
| Q8CGX0 | Igf2bp1 | 3 | 2 | 2 | 2 | 577 | 63.4 | 9.2 | 60 |
| B0BNK1 | Rab5c | 5 | 1 | 1 | 1 | 216 | 23.4 | 8.41 | 35 |
| A0A8I6A906 | Eif3g | 9 | 2 | 2 | 2 | 413 | 45.8 | 7.39 | 73 |
| P38656 | Ssb | 10 | 4 | 5 | 2 | 415 | 47.7 | 9.73 | 214 |
| A0A8I5ZU07 | Rcc1 | 2 | 1 | 1 | 1 | 434 | 46.6 | 8.35 | 47 |
| P04785 | P4hb | 9 | 4 | 5 | 4 | 509 | 56.9 | 4.93 | 134 |
| A0A8I6AS27 | Anxa5 | 9 | 4 | 4 | 4 | 398 | 44.3 | 6.02 | 159 |
| P68101 | Eif2s1 | 4 | 1 | 1 | 1 | 315 | 36.1 | 5.08 | 41 |
| Q5XIH3 | Ndufv1 | 4 | 2 | 2 | 2 | 464 | 50.7 | 8.07 | 75 |
| A0A8I6ANA7 | Eif3i | 14 | 5 | 5 | 5 | 365 | 40.8 | 5.87 | 184 |
| A0A8I6AAB9 | Ldha | 2 | 1 | 1 | 1 | 390 | 42.9 | 9.36 | 54 |
| A0A8L2QEA3 | Hsp90ab1 | 12 | 7 | 7 | 4 | 725 | 83.4 | 5.03 | 274 |
| Q6AYE3 | Tada2a | 4 | 2 | 2 | 2 | 443 | 51.4 | 7.24 | 53 |
| D3ZH53 | RGD1561871 | 8 | 1 | 1 | 1 | 169 | 19.5 | 9.51 | 60 |
| A0A0G2K2B8 | Ywhah | 9 | 3 | 3 | 2 | 274 | 31.7 | 5.21 | 62 |
| A0A096MJW2 | Far1 | 2 | 1 | 1 | 1 | 515 | 59.3 | 9.23 | 49 |
| A0A8I6AWL3 | Sar1b | 5 | 1 | 1 | 1 | 222 | 24.3 | 7.25 | 33 |
| G3V6S8 | Srsf6 | 16 | 5 | 5 | 5 | 339 | 39 | 11.46 | 142 |
| A0A8L2Q2H9 | Npm1 | 5 | 2 | 2 | 2 | 351 | 39.2 | 5.02 | 61 |
| A0A8I6A7U0 | Fkbp15 | 8 | 8 | 9 | 8 | 1215 | 133 | 5.19 | 312 |
| A0A8I5ZLD0 | Eif3l | 7 | 4 | 5 | 4 | 621 | 72.7 | 8.16 | 199 |
| A0A8I6GAH4 | Pdia3 | 8 | 4 | 4 | 4 | 510 | 57 | 6.21 | 127 |
| P16617 | Pgk1 | 8 | 3 | 3 | 3 | 417 | 44.5 | 7.9 | 74 |
| Q62826 | Hnrnpm | 16 | 12 | 14 | 1 | 690 | 73.7 | 8.75 | 455 |
| D4AAY6 | RGD1559972 | 7 | 1 | 1 | 1 | 157 | 17.6 | 11.11 | 77 |
| D3ZFJ6 | Lactb | 4 | 2 | 2 | 2 | 550 | 60.4 | 8.82 | 64 |
| A0A0H2UHL5 | Arpc2 | 6 | 2 | 2 | 2 | 300 | 34.3 | 7.36 | 48 |
| B5DF91 | Elavl1 | 16 | 5 | 5 | 5 | 326 | 36.1 | 9.04 | 137 |
| F1LYQ7 | LOC680700 | 6 | 1 | 1 | 1 | 221 | 25.3 | 9.85 | 35 |
| D4A5U3 | Tgm3 | 2 | 1 | 1 | 1 | 693 | 77.2 | 6.89 | 45 |
| A0A0G2JZS2 | Pabpc1 | 6 | 3 | 3 | 2 | 636 | 70.5 | 9.41 | 98 |
| P10760 | Ahcy | 7 | 3 | 3 | 3 | 432 | 47.5 | 6.54 | 115 |
| A0A8I5ZPD2 | Fau | 7 | 2 | 2 | 2 | 156 | 17 | 10.21 | 40 |
| D4A3X7 | Snx33 | 4 | 2 | 2 | 2 | 574 | 65.2 | 6.92 | 105 |
| A0A8I6AKB2 | Pfn1 | 4 | 1 | 1 | 1 | 209 | 22.5 | 8.79 | 33 |
| A0A0G2K1G0 | Bclaf1 | 1 | 2 | 2 | 2 | 1645 | 184.3 | 9.38 | 44 |
| D3ZTY9 | Nvl | 1 | 1 | 1 | 1 | 855 | 94.4 | 6.14 | 33 |
| P24368 | Ppib | 26 | 5 | 5 | 5 | 216 | 23.8 | 9.5 | 118 |
| A0A8I6GJ92 | Eprs1 | 1 | 1 | 1 | 1 | 1519 | 170.6 | 7.64 | 24 |
| A0A8L2PYP9 | Rbmx | 4 | 2 | 2 | 2 | 406 | 44.3 | 9.82 | 66 |
| A0A8I6A5S4 | Eif3e | 12 | 5 | 6 | 5 | 454 | 53.2 | 6.55 | 134 |
| F7FIS4 | Tpm1 | 17 | 8 | 8 | 1 | 326 | 37.4 | 4.72 | 215 |
| A0A8I6GMR8 | Srsf1 | 23 | 6 | 6 | 6 | 253 | 28.3 | 10.08 | 167 |
| A0A8I6G5T6 | Pkm | 12 | 5 | 5 | 5 | 591 | 64.4 | 7.44 | 183 |
| P04636 | Mdh2 | 17 | 5 | 5 | 5 | 338 | 35.7 | 8.68 | 179 |
| Q3B8Q1 | Ddx21 | 3 | 2 | 2 | 2 | 782 | 85.9 | 9.33 | 70 |
| P82995 | Hsp90aa1 | 4 | 3 | 3 | 1 | 733 | 84.8 | 5.01 | 109 |
| A0A8I6AFP0 | Washc5 | 12 | 15 | 15 | 15 | 1161 | 134.4 | 7.81 | 477 |
| D4A0E8 | Prmt5 | 2 | 1 | 1 | 1 | 637 | 72.6 | 6.27 | 27 |
| A0A8I6AE98 | Nme1 | 14 | 2 | 3 | 2 | 203 | 22.9 | 6.4 | 77 |
| A0A8J8XU90 | Myh9 | 27 | 49 | 53 | 49 | 1962 | 226.5 | 5.64 | 1737 |
| A6K669 | Anxa3 | 2 | 1 | 1 | 1 | 324 | 36.5 | 6.01 | 38 |
| G3V8L3 | Lmna | 16 | 10 | 11 | 10 | 665 | 74.3 | 6.98 | 310 |
| Q9WU82 | Ctnnb1 | 3 | 1 | 1 | 1 | 781 | 86 | 7.01 | 24 |
| A0A8I5ZY58 | LOC100362479 | 8 | 2 | 2 | 2 | 204 | 24.2 | 11.58 | 75 |
| A0A0G2K0Y2 | Igf2bp3 | 2 | 1 | 1 | 1 | 579 | 63.6 | 8.87 | 42 |
| D3ZWI4 | Sgo2 | 2 | 2 | 2 | 2 | 1162 | 130.4 | 8.65 | 81 |
| A0A8I6AEH8 | Pdia4 | 3 | 2 | 2 | 2 | 678 | 76.6 | 5.19 | 60 |
| A0A8I6AWT3 | ENSRNOG00000062927 | 6 | 2 | 2 | 1 | 257 | 27.7 | 10.49 | 73 |
| A0A0G2K4T7 | Gtf2i | 3 | 3 | 3 | 3 | 999 | 112.2 | 6.39 | 125 |
| A0A8I5ZNS1 | Atad2 | 1 | 1 | 1 | 1 | 1373 | 155.9 | 7.34 | 61 |
| A0A8L2Q8H0 | Magoh | 12 | 2 | 2 | 2 | 161 | 18.9 | 6.54 | 60 |
| A0A0G2K2Z0 | Eml4 | 7 | 6 | 6 | 6 | 979 | 108.8 | 6.68 | 178 |
| A0A8I5ZP79 | Eif3h | 3 | 1 | 1 | 1 | 352 | 39.6 | 6.71 | 55 |
| Q3MIE4 | Vat1 | 2 | 1 | 1 | 1 | 404 | 43.1 | 6.62 | 41 |
| A6IHJ4 | Rpl22l1 | 25 | 3 | 3 | 3 | 122 | 14.5 | 9.45 | 123 |
| P61354 | Rpl27 | 20 | 3 | 3 | 3 | 136 | 15.8 | 10.56 | 89 |
| D3ZC07 | Pkn3 | 1 | 1 | 1 | 1 | 958 | 107.1 | 7.91 | 67 |
| A0A8I6A562 | Cdc5l | 1 | 1 | 1 | 1 | 805 | 92.4 | 6.87 | 39 |
| A0A8I6A8Y6 | Sf3b2 | 2 | 2 | 2 | 2 | 885 | 98.4 | 5.39 | 73 |
| A6IF00 | LOC286911 | 7 | 2 | 2 | 2 | 247 | 26.3 | 7.49 | 76 |
| A0A8I5ZSD4 | Erh | 27 | 2 | 2 | 2 | 104 | 12.3 | 5.92 | 71 |
| A0A8I6GEL5 | Rps7-ps23 | 4 | 1 | 1 | 1 | 210 | 23.9 | 10.15 | 54 |
| Q9QZX1 | NRP | 7 | 5 | 5 | 2 | 715 | 77.4 | 4.67 | 165 |
| P27952 | Rps2 | 6 | 2 | 2 | 2 | 293 | 31.2 | 10.24 | 81 |
| O08651 | Phgdh | 3 | 1 | 1 | 1 | 533 | 56.5 | 6.71 | 73 |
| D3ZV50 | RGD1566136 | 5 | 1 | 1 | 1 | 194 | 22.6 | 10.32 | 58 |
| Q9ER34 | Aco2 | 1 | 1 | 1 | 1 | 780 | 85.4 | 7.83 | 41 |
| P62804 | H4c2 | 51 | 5 | 7 | 5 | 103 | 11.4 | 11.36 | 182 |
| P46462 | Vcp | 9 | 7 | 8 | 7 | 806 | 89.3 | 5.26 | 200 |
| P09034 | Ass1 | 2 | 1 | 1 | 1 | 412 | 46.5 | 7.78 | 24 |
| A6ISG8 | AK2 | 5 | 1 | 1 | 1 | 239 | 26.4 | 7.43 | 29 |
| G3V8M5 | Ppp4c | 3 | 1 | 1 | 1 | 307 | 35.1 | 5.06 | 34 |
| Q00438 | Ptbp1 | 12 | 5 | 5 | 5 | 556 | 59.3 | 9.16 | 185 |
| P62890 | Rpl30 | 18 | 2 | 2 | 2 | 115 | 12.8 | 9.63 | 75 |
| D3ZZ65 | Casp14 | 3 | 1 | 1 | 1 | 246 | 28.2 | 5.33 | 30 |
| A0A0H2UHX8 | Ilf2 | 2 | 1 | 1 | 1 | 477 | 53.1 | 5.71 | 63 |
| Q498R3 | Dnajc10 | 1 | 1 | 1 | 1 | 793 | 90.7 | 7.14 | 49 |
| Q7TPJ0 | Ssr1 | 3 | 1 | 1 | 1 | 319 | 35.6 | 4.45 | 33 |
| A0A8L2QJM1 | Krt80 | 3 | 2 | 2 | 2 | 502 | 55.7 | 7.09 | 85 |
| D4A3K5 | H1-1 | 11 | 3 | 3 | 1 | 214 | 22 | 10.99 | 92 |
| P00762 | Prss1 | 8 | 1 | 9 | 1 | 246 | 25.9 | 4.89 | 133 |
| A6KCA2 | Fkbp11 | 5 | 1 | 1 | 1 | 201 | 22.2 | 9.25 | 63 |
| D3ZBN0 | H1-5 | 10 | 3 | 3 | 2 | 222 | 22.6 | 10.96 | 104 |
| D4A985 | Prdm11 | 6 | 7 | 7 | 7 | 1172 | 133.7 | 6.15 | 201 |
| A0A8I5Y7R1 | Pdia6 | 14 | 6 | 6 | 6 | 481 | 52.5 | 5.2 | 274 |
| B5DFC8 | Eif3c | 9 | 7 | 8 | 7 | 911 | 105.4 | 5.78 | 187 |
| A0A0H2UHU0 | Rps25 | 21 | 4 | 4 | 4 | 142 | 15.5 | 10.11 | 152 |
| A0A8I6A888 | Uqcrc2 | 2 | 1 | 1 | 1 | 452 | 48.4 | 9.06 | 55 |
| D4A7U6 | Lsm3 | 12 | 1 | 1 | 1 | 102 | 11.8 | 4.7 | 47 |
| F1LUV3 | ENSRNOG00000066746 | 17 | 5 | 6 | 5 | 398 | 43.6 | 8.62 | 205 |
| A0A8I5ZR44 | Snrpd2 | 7 | 1 | 1 | 1 | 124 | 14.1 | 9.85 | 60 |
| Q6IFV1 | Krt14 | 16 | 8 | 16 | 1 | 485 | 52.7 | 5.16 | 533 |
| A0A8L2QCF0 | Mbp | 6 | 2 | 2 | 2 | 328 | 35.8 | 9.88 | 72 |
| A6JEP9 | Serpina9_predicted | 2 | 1 | 2 | 1 | 417 | 46.8 | 9.6 | 86 |
| A0A8I5ZJ51 | Myo5a | 1 | 1 | 1 | 1 | 1877 | 218 | 8.53 | 34 |
| G3V8V1 | Grn | 7 | 3 | 3 | 3 | 602 | 65.1 | 6.73 | 36 |
| P85834 | Tufm | 13 | 6 | 6 | 6 | 452 | 49.5 | 7.56 | 157 |
| A6JSM6 | Hic2_predicted | 2 | 1 | 1 | 1 | 622 | 67.2 | 6.35 | 27 |
| F1M577 | AABR07033607.1 | 5 | 1 | 1 | 1 | 205 | 23 | 10.27 | 51 |
| P63018 | Hspa8 | 27 | 14 | 15 | 12 | 646 | 70.8 | 5.52 | 472 |
| A6JG60 | Tagln2 | 4 | 1 | 2 | 1 | 268 | 30 | 8.53 | 57 |
| A0A0G2JWX4 | Krt2 | 7 | 6 | 9 | 3 | 685 | 69.2 | 7.69 | 268 |
| A0A8I5ZUB2 | Oscp1 | 14 | 4 | 4 | 4 | 190 | 21.2 | 10.58 | 138 |
| A0A8I6AG52 | LOC679899 | 14 | 2 | 2 | 2 | 165 | 18.4 | 9.45 | 61 |
| D3ZZ95 | LOC100361060 | 9 | 1 | 1 | 1 | 106 | 12.4 | 11.66 | 34 |
| Q4KM71 | Sfpq | 6 | 4 | 4 | 3 | 699 | 75.4 | 9.44 | 112 |
| A0A8I5ZMI8 | LOC120093742 | 11 | 7 | 7 | 3 | 552 | 59.3 | 8.34 | 219 |
| Q80YN6 | LOC641544 | 2 | 1 | 1 | 1 | 461 | 49.8 | 4.79 | 41 |
| P62738 | Acta2 | 38 | 12 | 23 | 4 | 377 | 42 | 5.39 | 609 |
| A6J2C0 | rCG_21137 | 13 | 4 | 4 | 4 | 288 | 32 | 8.68 | 100 |
| F1M6V1 | Hp1bp3 | 2 | 1 | 1 | 1 | 578 | 63.5 | 9.76 | 27 |
| B2RYP6 | Luc7l2 | 2 | 1 | 1 | 1 | 392 | 46.6 | 10.1 | 46 |
| A0A8L2QEH3 | Rps19 | 19 | 4 | 4 | 4 | 156 | 17.3 | 10.51 | 107 |
| A0A8I6AI02 | Rtcb | 4 | 2 | 2 | 2 | 505 | 55.1 | 8.34 | 62 |
| A6IDY9 | Ndufa4 | 22 | 2 | 2 | 2 | 82 | 9.3 | 9.52 | 55 |
| B0BN81 | Rps5 | 4 | 1 | 1 | 1 | 204 | 22.9 | 9.72 | 80 |
| A0A8I6A2P1 | Srsf3 | 4 | 1 | 1 | 1 | 213 | 24.4 | 9.5 | 25 |
| Q6AYL5 | Sf3b4 | 2 | 1 | 1 | 1 | 424 | 44.3 | 8.56 | 29 |
| A0A8J8XVI1 | ENSRNOG00000070169 | 13 | 1 | 1 | 1 | 125 | 13.9 | 7.24 | 20 |
| D3ZSX8 | Kat14 | 1 | 1 | 1 | 1 | 779 | 88.1 | 6.6 | 42 |
| A6HJT4 | Arf2 | 7 | 2 | 4 | 1 | 255 | 28.3 | 7.28 | 168 |
| A6KAR2 | Ddx41 | 1 | 1 | 1 | 1 | 622 | 69.8 | 6.8 | 48 |
| Q6IG08 | Krt83 | 5 | 3 | 3 | 3 | 555 | 62.2 | 7.49 | 94 |
| A0A8I6A3B1 | Ncoa4 | 2 | 1 | 1 | 1 | 641 | 72.5 | 5.9 | 69 |
| A0A8L2QDD2 | Tpm3 | 33 | 10 | 10 | 7 | 248 | 28.9 | 4.75 | 327 |
| F1MA18 | Ybx3 | 5 | 2 | 2 | 2 | 452 | 48.9 | 10.86 | 65 |
| A0A8I5Y8E5 | Ppp1r12a | 1 | 1 | 1 | 1 | 1070 | 119.4 | 5.5 | 46 |
| A0A0G2JSL0 | LOC100360846 | 4 | 1 | 1 | 1 | 238 | 25.3 | 5 | 57 |
| M0R6L4 | LOC100365839 | 11 | 3 | 3 | 3 | 264 | 29.9 | 9.73 | 98 |
| P00507 | Got2 | 3 | 1 | 1 | 1 | 430 | 47.3 | 9 | 27 |
| A0A8I5ZL35 | ENSRNOG00000063418 | 8 | 1 | 1 | 1 | 102 | 11.4 | 10.33 | 38 |
| P18420 | Psma1 | 4 | 1 | 1 | 1 | 263 | 29.5 | 6.61 | 0 |
| F1LMV6 | Dsp | 5 | 15 | 17 | 15 | 2877 | 332.2 | 6.83 | 527 |
| A0A8I6G5X8 | Hnrnph1 | 10 | 4 | 5 | 3 | 472 | 51.2 | 6.8 | 140 |
| P05964 | S100a6 | 17 | 2 | 2 | 2 | 89 | 10 | 5.48 | 85 |
| D3ZXI0 | Pycr1 | 3 | 1 | 1 | 1 | 383 | 39.7 | 7.06 | 43 |
| A0A8L2R0P3 | Aldoa | 9 | 3 | 3 | 3 | 418 | 45.1 | 7.91 | 119 |
| F1LRK9 | Ppp4r1 | 1 | 1 | 1 | 1 | 951 | 105.5 | 4.79 | 39 |
| A6I6G9 | Serpinh1 | 2 | 1 | 1 | 1 | 417 | 46.5 | 8.82 | 38 |
| A0A0G2K8V3 | Thoc2 | 1 | 1 | 1 | 1 | 1621 | 185.5 | 8.41 | 0 |
| P35427 | Rpl13a | 5 | 1 | 1 | 1 | 203 | 23.5 | 11.02 | 63 |
| P39951 | Cdk1 | 6 | 2 | 2 | 2 | 297 | 34.1 | 8.41 | 95 |
| Q5XIH7 | Phb2 | 9 | 3 | 3 | 3 | 299 | 33.3 | 9.83 | 100 |
| Q09073 | Slc25a5 | 18 | 6 | 6 | 6 | 298 | 32.9 | 9.73 | 167 |
| Q5M7U6 | Actr2 | 3 | 1 | 2 | 1 | 394 | 44.7 | 6.74 | 94 |
| A0A8I6A8J5 | rCG_26455 | 2 | 1 | 1 | 1 | 310 | 34.6 | 6.52 | 45 |
| A0A8I6GLV2 | Nt5c2 | 2 | 1 | 1 | 1 | 586 | 67.7 | 6.74 | 29 |
| A0A8I5ZXA1 | Myl12a | 20 | 4 | 5 | 4 | 208 | 24 | 5.33 | 214 |
| A0A8L2QA00 | Arpc5l | 16 | 2 | 2 | 2 | 153 | 17.1 | 6.8 | 76 |
| G3V6D3 | Atp5f1b | 27 | 11 | 11 | 11 | 529 | 56.3 | 5.27 | 319 |
| Q9QZA2 | Pdcd6ip | 4 | 3 | 3 | 3 | 873 | 96.6 | 6.52 | 115 |
| A6KSD6 | rCG_42490 | 42 | 8 | 10 | 8 | 185 | 20.9 | 5.31 | 323 |
| Q8CFC1 | Riox2 | 2 | 1 | 1 | 1 | 465 | 53.2 | 6.9 | 45 |
| P07895 | Sod2 | 4 | 1 | 1 | 1 | 222 | 24.7 | 8.81 | 44 |
| A0A8I6ALD4 | Tjp2 | 2 | 2 | 2 | 2 | 1239 | 139.7 | 7.24 | 57 |
| Q6TUH8 | LOC306079 | 7 | 2 | 2 | 2 | 354 | 38.3 | 10.15 | 71 |
| F7FDP3 | Fus | 14 | 6 | 6 | 6 | 518 | 52.6 | 9.36 | 107 |
| A0A8I5ZP60 | Snw1 | 3 | 2 | 2 | 2 | 536 | 61.4 | 9.5 | 70 |
| A0A8L2Q4X8 | Gemin4 | 3 | 1 | 1 | 1 | 332 | 37.3 | 5.71 | 39 |
| P0DMW0 | Hspa1a | 7 | 4 | 4 | 2 | 641 | 70.1 | 5.82 | 108 |
| F1M953 | Hspa9 | 27 | 17 | 21 | 17 | 679 | 73.7 | 6.16 | 530 |
| F1LPV0 | Nars1 | 1 | 1 | 1 | 1 | 558 | 64.1 | 5.86 | 58 |
| A0A8I6ACB9 | Dlst | 7 | 3 | 3 | 3 | 464 | 50.4 | 8.53 | 144 |
| A6HZL1 | Fkbp2_predicted | 7 | 1 | 1 | 1 | 171 | 18.4 | 9.14 | 56 |
| A0A8I5ZKV9 | Ddx5 | 23 | 14 | 15 | 8 | 617 | 69.4 | 9.11 | 464 |
| Q6AXV4 | Samm50 | 2 | 1 | 1 | 1 | 469 | 51.9 | 6.8 | 18 |
| Q6P502 | Cct3 | 4 | 2 | 2 | 2 | 545 | 60.6 | 6.64 | 65 |
| P97536 | Cand1 | 1 | 1 | 1 | 1 | 1230 | 136.3 | 5.78 | 58 |
| A0A8I5ZX14 | Tgm1 | 1 | 1 | 1 | 1 | 835 | 91.9 | 6.47 | 38 |
| A0A0G2JSG8 | Ubd | 29 | 4 | 4 | 4 | 161 | 18 | 8.44 | 121 |
| A0A8I5ZKF7 | Anxa1 | 6 | 2 | 2 | 2 | 362 | 40.7 | 7.72 | 105 |
| Q71DI1 |  | 20 | 2 | 2 | 2 | 110 | 11.3 | 6.54 | 93 |
| A0A8I5Y6U8 | Psmb1 | 4 | 1 | 1 | 1 | 240 | 26.4 | 7.8 | 32 |
| P18418 | Calr | 2 | 1 | 1 | 1 | 416 | 48 | 4.49 | 52 |
| D3ZLH9 | LOC680385 | 7 | 3 | 4 | 1 | 402 | 46.5 | 9.64 | 214 |
| A0A8I6ATC2 | Lgals3 | 4 | 1 | 1 | 1 | 278 | 29 | 8.69 | 20 |
| F1M7P8 | Spryd3 | 2 | 1 | 1 | 1 | 448 | 50.4 | 6.37 | 47 |
| P20788 | Uqcrfs1 | 3 | 1 | 1 | 1 | 274 | 29.4 | 8.87 | 29 |
| A0A0G2K5T2 | Plec | 0 | 2 | 2 | 2 | 4692 | 534 | 5.91 | 72 |
| A0A8I5ZMC0 | Fth1 | 18 | 4 | 4 | 4 | 204 | 23.4 | 6.27 | 122 |
| A0A8I6GG73 | Calm2 | 5 | 1 | 1 | 1 | 193 | 21.7 | 4.56 | 57 |
| M0RBB1 | Alyref | 7 | 1 | 1 | 1 | 256 | 27 | 11.15 | 35 |
| A6JNR5 | Ilf3 | 5 | 5 | 5 | 5 | 910 | 97.5 | 8.95 | 131 |
| A0A8I6A9C1 | Immt | 3 | 2 | 2 | 2 | 776 | 86.2 | 5.85 | 62 |
| A0A8L2QBS3 | Eif5a | 5 | 1 | 1 | 1 | 167 | 18 | 5.78 | 49 |
| Q63279 | Krt19 | 16 | 7 | 7 | 3 | 403 | 44.6 | 5.31 | 208 |
| A0A0G2JZI0 | Safb | 2 | 2 | 2 | 2 | 942 | 105.7 | 5.38 | 74 |
| A0A8L2QZ13 | Rhoa | 3 | 1 | 1 | 1 | 248 | 27.6 | 8.09 | 40 |
| A0A8I5ZXU0 | Mtrex | 1 | 1 | 1 | 1 | 1109 | 125.2 | 6.27 | 55 |
| B2RZ79 | Iscu | 4 | 1 | 1 | 1 | 167 | 18 | 9.29 | 39 |
| G3V826 | Tkt | 4 | 2 | 2 | 2 | 661 | 71.7 | 7.64 | 68 |
| Q4FZY0 | Efhd2 | 11 | 2 | 2 | 2 | 239 | 26.7 | 5.07 | 114 |
| D4AC36 | Eif3f | 16 | 5 | 5 | 5 | 361 | 38 | 5.45 | 195 |
| M0R9X8 | Dync1h1 | 0 | 1 | 1 | 1 | 4646 | 531.9 | 6.42 | 34 |
| A0A8L2Q7W8 | Gars1 | 2 | 1 | 1 | 1 | 729 | 81.8 | 6.6 | 43 |
| E9PSX8 | Sipa1 | 1 | 1 | 1 | 1 | 1040 | 112.6 | 6.47 | 17 |
| A6JIA5 | Eif3s10 | 5 | 7 | 7 | 7 | 1354 | 163.1 | 6.84 | 193 |
| F2Z3Q8 | Kpnb1 | 1 | 1 | 1 | 1 | 876 | 97.1 | 4.78 | 56 |
| A0A8I6ABZ7 | Dhx9 | 4 | 5 | 5 | 5 | 1392 | 150.1 | 6.89 | 190 |
| A0A8I6AM55 | Lima1 | 6 | 5 | 6 | 5 | 773 | 85.8 | 6.19 | 243 |
| A0A8I5Y1C4 | AABR07021736.1 | 11 | 1 | 1 | 1 | 75 | 8.4 | 9.76 | 57 |
| A0A0G2K0W9 | Psma7 | 4 | 1 | 1 | 1 | 254 | 28.4 | 8.46 | 58 |
| A0A0G2JZ52 | Hnrnpu | 13 | 7 | 7 | 7 | 803 | 87.9 | 6.09 | 181 |
| Q10758 | Krt8 | 13 | 7 | 9 | 3 | 483 | 54 | 6 | 238 |
| Q3T1K5 | Capza2 | 12 | 3 | 3 | 3 | 286 | 32.9 | 5.85 | 102 |
| F1LX07 | Slc25a12 | 1 | 1 | 1 | 1 | 676 | 74.3 | 8.4 | 48 |
| A0A8I6G6Q3 | Serbp1 | 7 | 2 | 2 | 2 | 423 | 47 | 9.19 | 128 |
| D4ACB8 | Cct8 | 8 | 4 | 4 | 4 | 548 | 59.6 | 5.54 | 102 |
| A0A8I5ZQG4 | Rpl24 | 8 | 1 | 2 | 1 | 157 | 17.8 | 11.24 | 104 |
| Q4G061 | Eif3b | 7 | 4 | 5 | 4 | 797 | 90.9 | 5.08 | 136 |
| A0A8I6ACT0 | LOC103694176 | 3 | 2 | 2 | 2 | 573 | 60.8 | 6 | 74 |
| A0A8L2R4T1 | Jup | 11 | 7 | 8 | 7 | 749 | 82.3 | 6.38 | 285 |
| A0A8I6A5F1 | Pabpn1 | 9 | 3 | 3 | 3 | 320 | 34.2 | 5.17 | 118 |
| Q6P2A7 |  | 3 | 1 | 1 | 1 | 428 | 47.5 | 7.15 | 31 |
| G3V7Q7 | Iqgap1 | 1 | 2 | 2 | 2 | 1657 | 188.7 | 6.43 | 75 |
| A0A8I5ZV49 | Flna | 8 | 19 | 19 | 19 | 2647 | 281 | 6.05 | 434 |
| A0A8I5Y8E3 | Tubb4b | 13 | 6 | 9 | 1 | 547 | 60.6 | 5.17 | 333 |
| A0A8I6A4L1 | Mycbp2 | 0 | 1 | 1 | 1 | 4844 | 529.8 | 7.15 | 41 |
| F7EPV5 | Rpl28 | 18 | 3 | 3 | 3 | 165 | 18.5 | 12.02 | 89 |
| Q80X08 | Washc2 | 4 | 5 | 5 | 5 | 1328 | 145.1 | 4.77 | 171 |
| P83883 | Rpl36a | 9 | 2 | 2 | 2 | 106 | 12.4 | 10.58 | 70 |
| A0A8L2UK94 | Slc25a17 | 5 | 3 | 3 | 3 | 641 | 72.1 | 8.62 | 73 |
| A0A8I6GLU8 | Tardbp | 2 | 1 | 1 | 1 | 414 | 44.5 | 6.7 | 50 |
| P69897 | Tubb5 | 16 | 6 | 9 | 1 | 444 | 49.6 | 4.89 | 337 |
| A6JSC0 | RGD1566176_predicted | 3 | 3 | 3 | 3 | 1405 | 148.6 | 9.06 | 99 |
| A0A8L2UJT5 | Rps23 | 6 | 1 | 1 | 1 | 181 | 19.9 | 10.71 | 44 |
| B2GV06 | Oxct1 | 1 | 1 | 1 | 1 | 520 | 56.2 | 8.47 | 43 |
| O88656 | Arpc1b | 8 | 2 | 2 | 2 | 372 | 41 | 8.35 | 83 |
| A0A8L2Q9C5 | Tsg101 | 2 | 1 | 1 | 1 | 422 | 47.4 | 7.69 | 35 |
| A6IT06 | Sh3bgrl3_predicted | 11 | 1 | 1 | 1 | 93 | 10.4 | 9.73 | 51 |
| A0A8I6GD73 | Rpl18 | 13 | 3 | 3 | 3 | 254 | 28.9 | 11.43 | 41 |
| A0A8I6G659 | H2az2 | 13 | 2 | 2 | 1 | 143 | 15.2 | 10.29 | 81 |
| A0A8J8YIF6 | Rpl7a | 13 | 4 | 4 | 4 | 294 | 32.8 | 10.71 | 125 |
| A0A0G2JST3 | Krt1 | 5 | 4 | 5 | 3 | 625 | 64.7 | 7.93 | 193 |
| A6HQW0 | Shmt2 | 2 | 1 | 1 | 1 | 521 | 57.5 | 8.05 | 49 |
| A0A0G2K235 | Rab1a | 3 | 1 | 1 | 1 | 323 | 36.4 | 9.85 | 54 |
| P63164 | Snrpn | 3 | 1 | 1 | 1 | 240 | 24.6 | 11.19 | 38 |
| P42123 | Ldhb | 3 | 1 | 1 | 1 | 334 | 36.6 | 6.05 | 62 |
| A0A8I6AFZ5 | Srsf7 | 5 | 1 | 2 | 1 | 238 | 27.4 | 11.82 | 94 |
| G3V7Y3 | Atp5f1d | 4 | 1 | 1 | 1 | 229 | 24 | 7.37 | 31 |
| D3ZAN3 | Ganab | 1 | 1 | 1 | 1 | 797 | 90.5 | 6.2 | 46 |
| D3ZD97 | Dhx15 | 2 | 1 | 1 | 1 | 795 | 90.9 | 7.46 | 31 |
| A0A0G2JXI9 | ENSRNOG00000068034 | 10 | 3 | 3 | 3 | 186 | 20.7 | 10.42 | 74 |
| Q641Y0 | Ddost | 5 | 2 | 2 | 2 | 441 | 48.9 | 5.97 | 35 |
| P14604 | Echs1 | 3 | 1 | 1 | 1 | 290 | 31.5 | 8.13 | 25 |
| A6JM43 | Vim | 29 | 13 | 14 | 12 | 466 | 53.7 | 5.12 | 443 |
| Q5RJR8 | Lrrc59 | 3 | 1 | 1 | 1 | 307 | 34.8 | 9.52 | 41 |
| A0A8I6A289 | LOC108349691 | 13 | 2 | 2 | 2 | 136 | 15.3 | 11 | 70 |
| A0A8I6G5P0 | Eif2s2 | 3 | 1 | 1 | 1 | 347 | 39.9 | 5.91 | 58 |
| A0A0G2K2V6 | Krt10 | 12 | 8 | 15 | 6 | 559 | 56.8 | 5.11 | 522 |
| P05197 | Eef2 | 1 | 1 | 1 | 1 | 858 | 95.2 | 6.83 | 59 |
| M0R735 | Syncrip | 3 | 2 | 2 | 2 | 602 | 67.7 | 8.53 | 120 |
| A6JFB7 | rCG_30479 | 18 | 4 | 4 | 4 | 268 | 31.3 | 10.93 | 113 |
| A0A8I6G747 | Ptma | 9 | 1 | 1 | 1 | 114 | 12.8 | 3.95 | 57 |
| Q6AYA1 | Gar1 | 4 | 1 | 1 | 1 | 226 | 23 | 10.92 | 40 |
| A6JXL0 | rCG_32122 | 3 | 1 | 1 | 1 | 307 | 35.6 | 9.7 | 51 |
| P10860 | Glud1 | 6 | 4 | 4 | 4 | 558 | 61.4 | 8 | 125 |
| A6HK13 | RGD1308813 | 2 | 1 | 1 | 1 | 483 | 55.6 | 4.84 | 78 |
| A0A8L2QBJ7 | Fbxo11 | 2 | 2 | 2 | 2 | 933 | 104.2 | 7.05 | 68 |
| D4A4Q6 | Hoatz | 15 | 1 | 1 | 1 | 168 | 19.2 | 8.51 | 0 |
| P63102 | Ywhaz | 17 | 4 | 4 | 3 | 245 | 27.8 | 4.79 | 157 |
| Q64599 | LOC286987 | 9 | 2 | 2 | 2 | 216 | 24.1 | 7.66 | 73 |
| A0A0G2K4H7 | Krt78 | 1 | 1 | 1 | 1 | 1013 | 107.8 | 7.14 | 28 |
| Q9QXQ0 | Actn4 | 8 | 7 | 8 | 5 | 911 | 104.8 | 5.44 | 177 |
| Q794E4 | Hnrnpf | 13 | 4 | 5 | 3 | 415 | 45.7 | 5.49 | 195 |
| Q5FVM4 | Nono | 4 | 2 | 2 | 1 | 476 | 54.9 | 8.95 | 58 |
| A0A8I5ZMM6 | Atp5po | 5 | 1 | 1 | 1 | 216 | 23.8 | 9.94 | 80 |
| A0A8I6ANH7 | Washc1 | 9 | 4 | 4 | 4 | 484 | 52.3 | 5.52 | 106 |
| D3ZN03 | LOC100362751 | 17 | 2 | 2 | 2 | 115 | 11.8 | 4.59 | 62 |
| P62859 | Rps28 | 46 | 3 | 3 | 3 | 69 | 7.8 | 10.7 | 98 |
| A0A8I6A7A4 | Ddx3y | 11 | 7 | 8 | 6 | 674 | 75.7 | 7.11 | 278 |
| Q63570 | Psmc4 | 2 | 1 | 1 | 1 | 418 | 47.4 | 5.21 | 21 |
| A0A8L2RBV8 | Rpl39 | 14 | 1 | 1 | 1 | 73 | 8.9 | 12.25 | 0 |
| A0A8I6G5G7 | Kxd1 | 19 | 4 | 6 | 4 | 232 | 26.8 | 9.83 | 159 |
| Q5XIF6 | Tuba4a | 9 | 4 | 4 | 1 | 448 | 49.9 | 5.06 | 116 |
| Q9WU06 | Avil | 6 | 5 | 5 | 5 | 819 | 91.8 | 5.34 | 164 |
| A0A8I6AI37 | Snrpd3 | 7 | 1 | 1 | 1 | 139 | 15.3 | 9.32 | 92 |
| F7F0R0 | Tmod3 | 6 | 2 | 2 | 2 | 359 | 40.1 | 4.79 | 59 |
| E9PT66 | Sf3b3 | 1 | 1 | 1 | 1 | 1217 | 135.5 | 5.26 | 18 |
| Q5XIC6 | Psmd12 | 2 | 1 | 1 | 1 | 456 | 52.9 | 7.36 | 38 |
| P11232 | Txn | 21 | 2 | 2 | 2 | 105 | 11.7 | 4.92 | 111 |
| A0A0G2JYA4 | LOC100362453 | 3 | 1 | 1 | 1 | 309 | 35.5 | 5.43 | 33 |
