# Supplementary material for: Mechanistic insights into the role of FAT10 in modulating NCOA4-mediated ferroptosis in pancreatic acinar cells during acute pancreatitis
Source: Cell Death Dis. 2025 May 15;16(1):385. doi: 10.1038/s41419-025-07715-9 (PMC12081885; doi:10.1038/s41419-025-07715-9)

Fig.1I

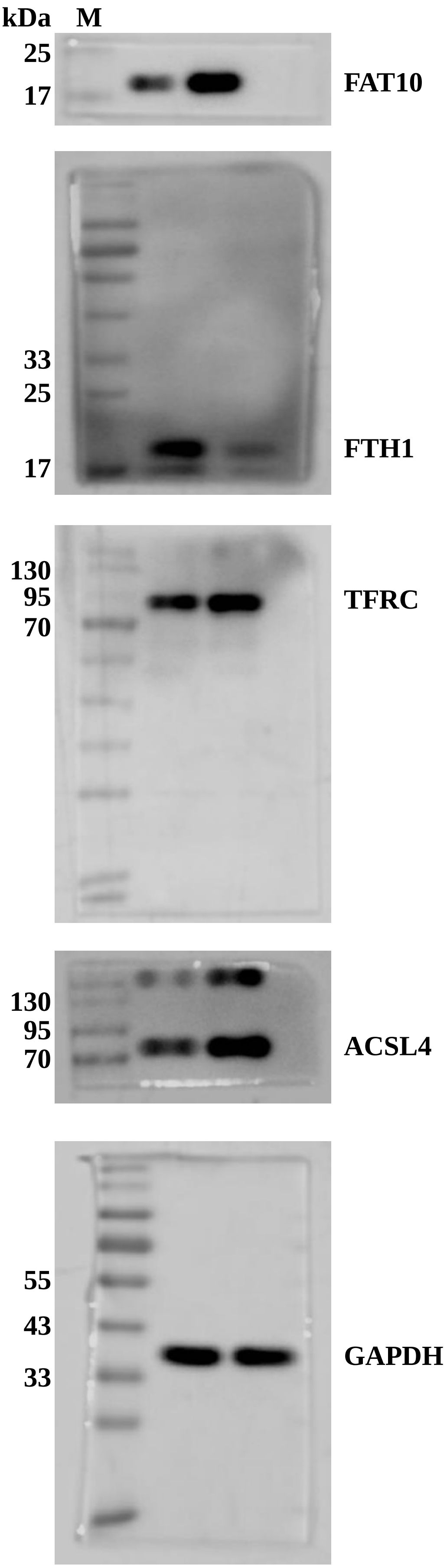
kDa
M
25
17
FAT10
33
25
17
FTH1
130
95
70
TFRC
130
95
70
ACSL4
55
43
33
GAPDH

Fig.2A

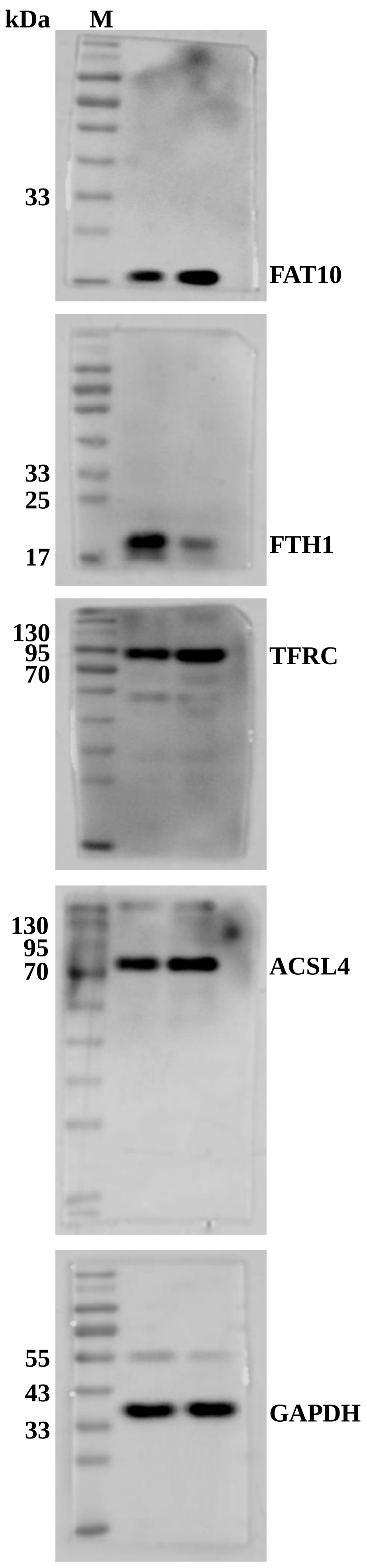
kDa
M
33
FAT10
33
25
17
FTH1
130
95
70
TFRC
130
95
70
ACSL4
55
43
33
GAPDH

Fig. 2E

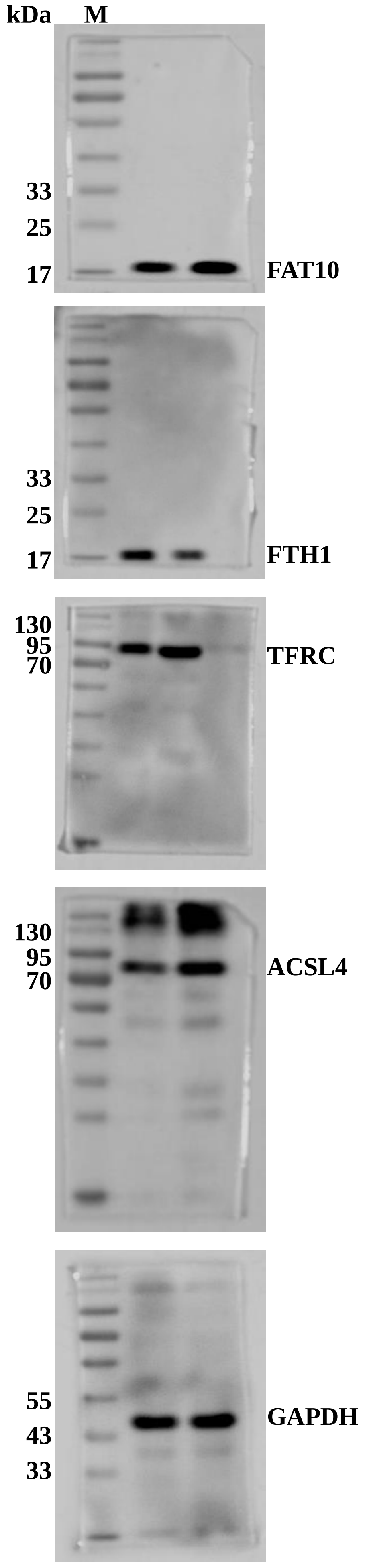
kDa
M
33
25
17
FAT10
33
25
17
FTH1
130
95
70
TFRC
130
95
70
ACSL4
55
43
33
GAPDH

Fig.2I

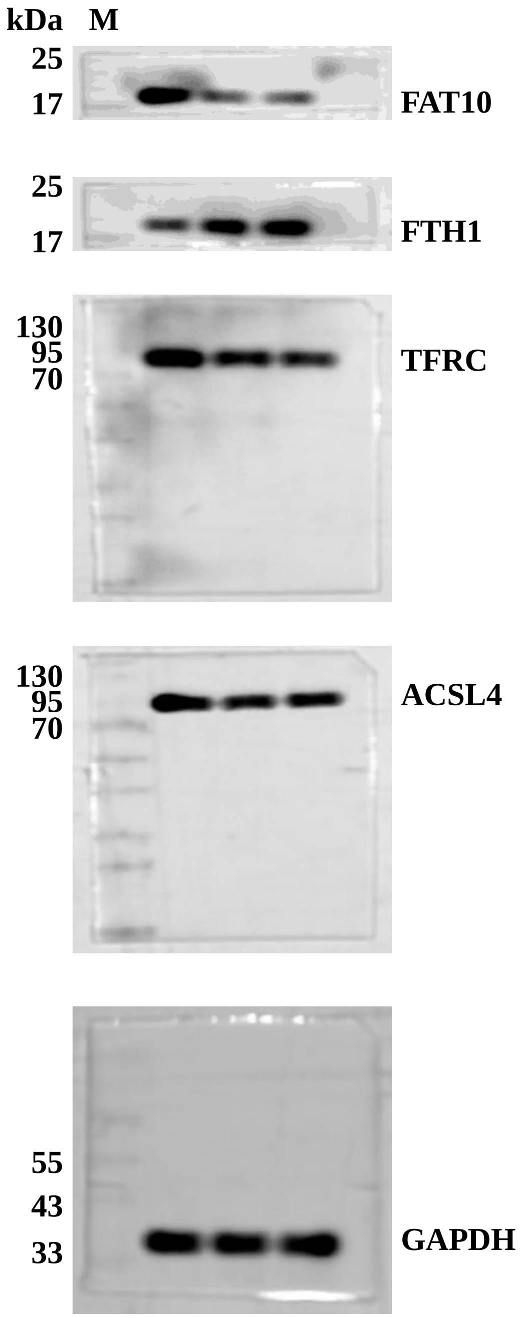
kDa
M
25
17
FAT10
25
17
FTH1
130
95
70
TFRC
130
95
70
ACSL4
55
43
33
GAPDH

Fig.2M

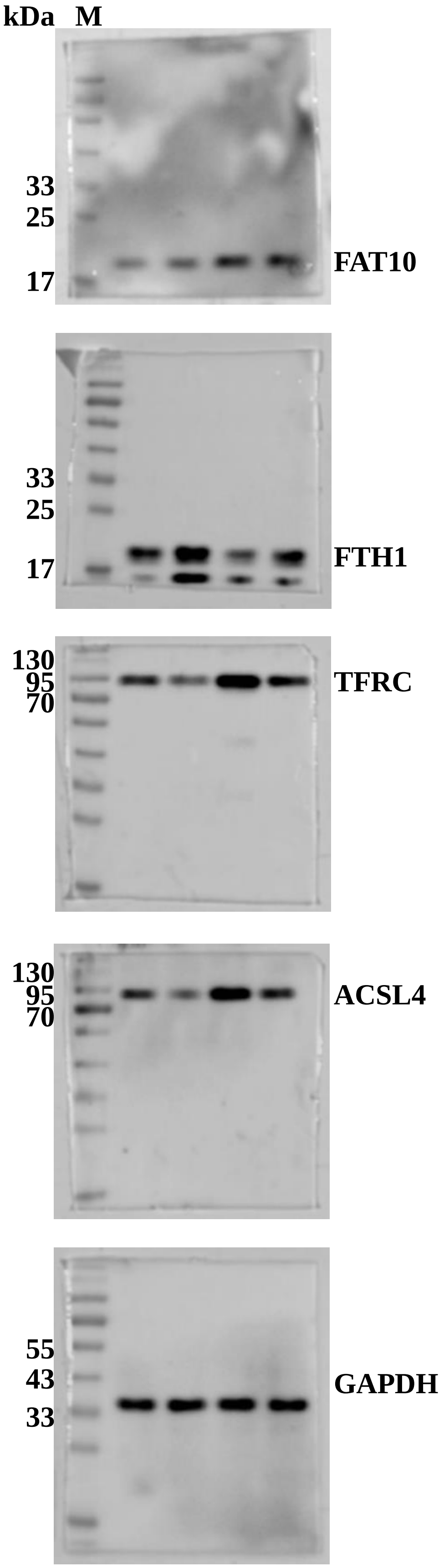

kDa
M
33
25
17
FAT10
33
25
17
FTH1
130
95
70
TFRC
130
95
70
ACSL4
55
43
33
GAPDH

Fig.2N

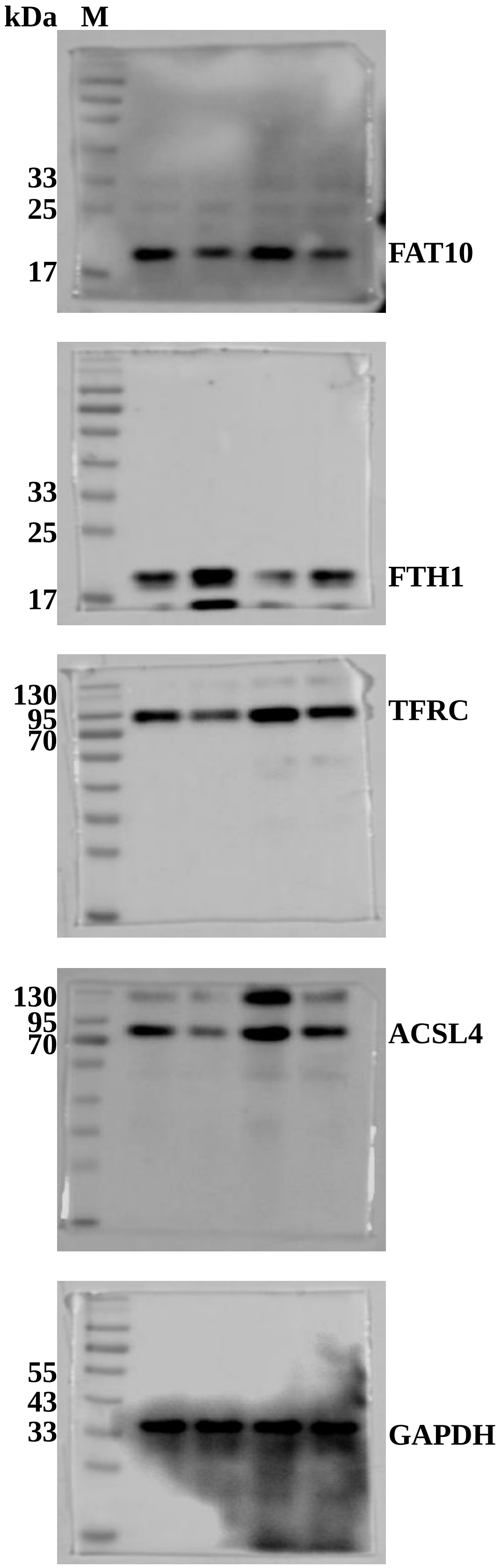

kDa
M
33
25
17
FAT10
33
25
17
FTH1
130
95
70
TFRC
130
95
70
ACSL4
55
43
33
GAPDH

Fig.3A

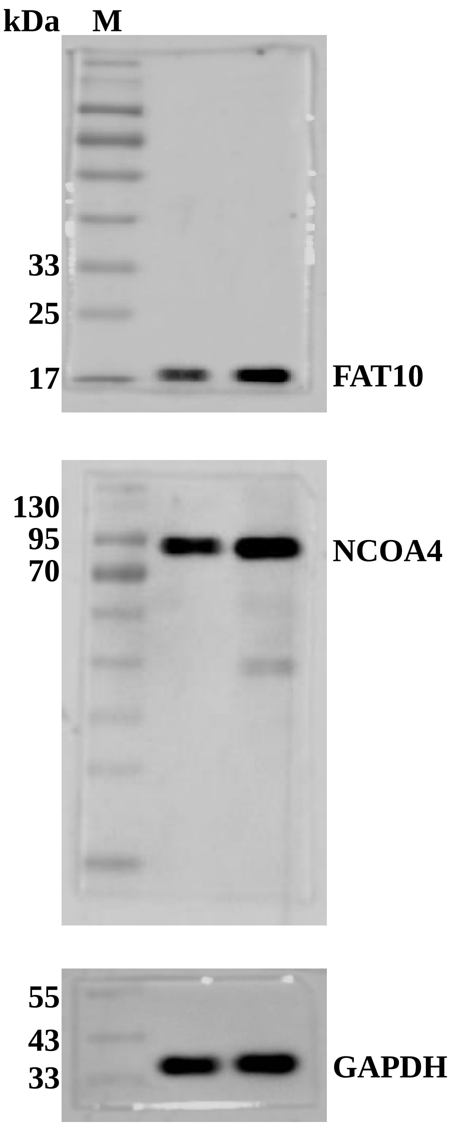
kDa
M
33
25
17
FAT10
130
95
70
NCOA4
55
43
33
GAPDH

Fig.3B

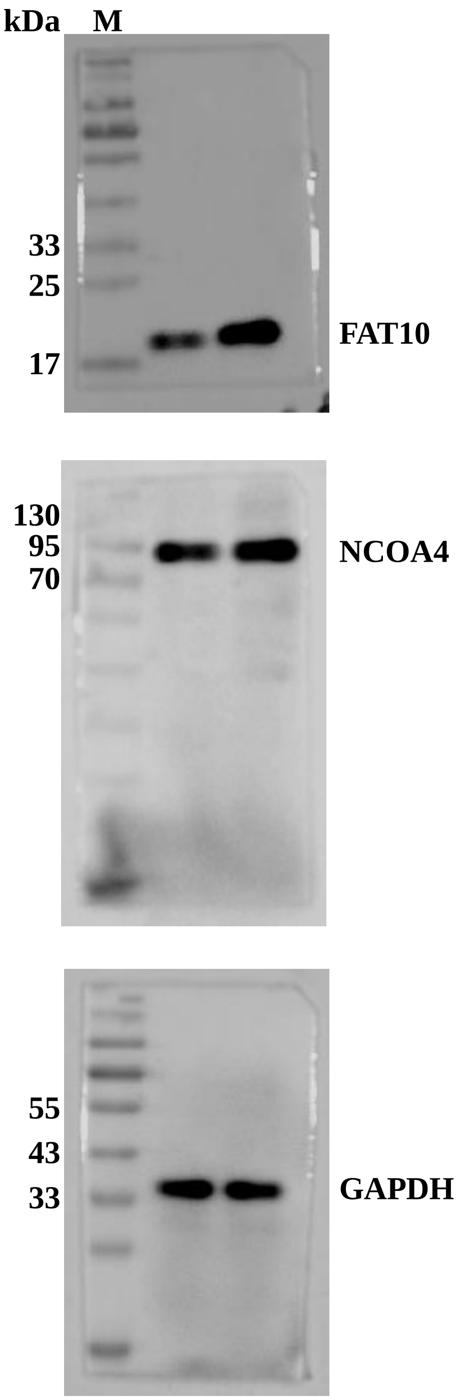
kDa
M
33
25
17
FAT10
130
95
70
NCOA4
55
43
33
GAPDH

Fig.3C

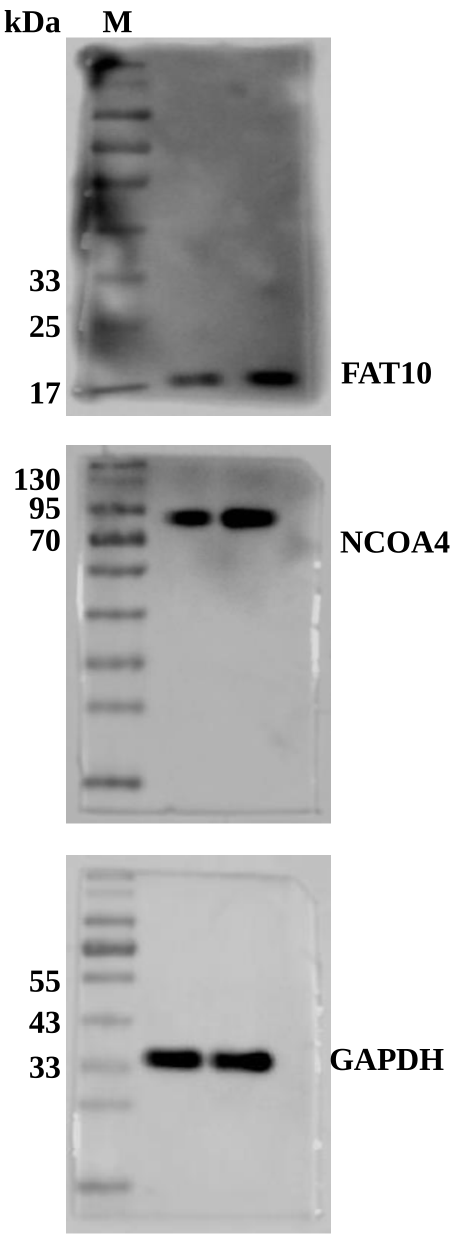
kDa
M
33
25
17
FAT10
130
95
70
NCOA4
55
43
33
GAPDH

Fig.3D

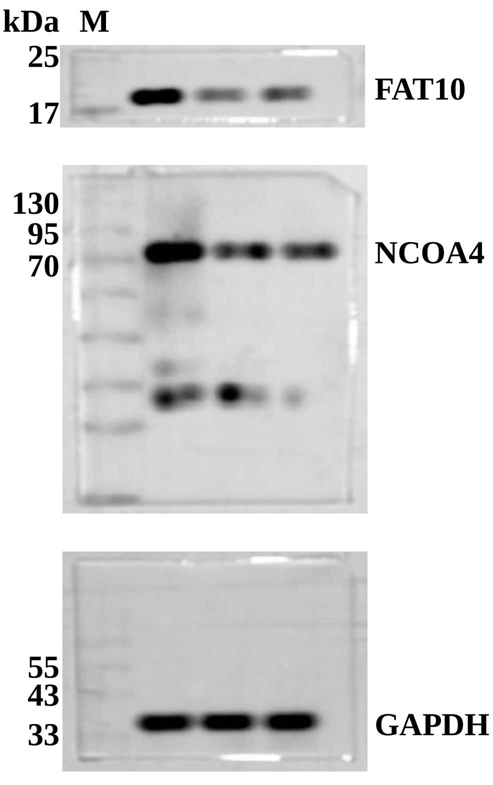
kDa
M
25
17
FAT10
130
95
70
NCOA4
55
43
33
GAPDH

Fig.3E

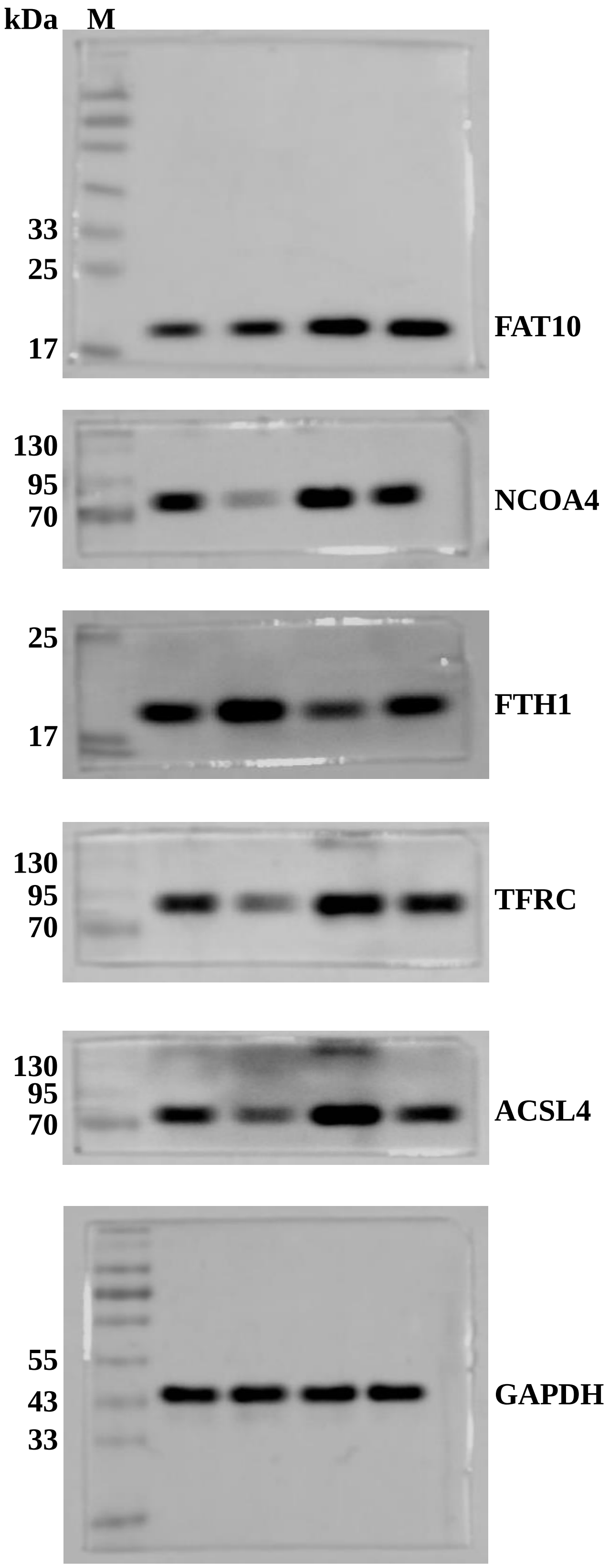
kDa
M
33
25
17
FAT10
130
95
70
NCOA4
25
17
FTH1
130
95
70
TFRC
130
95
70
ACSL4
55
43
33
GAPDH

Fig.3H

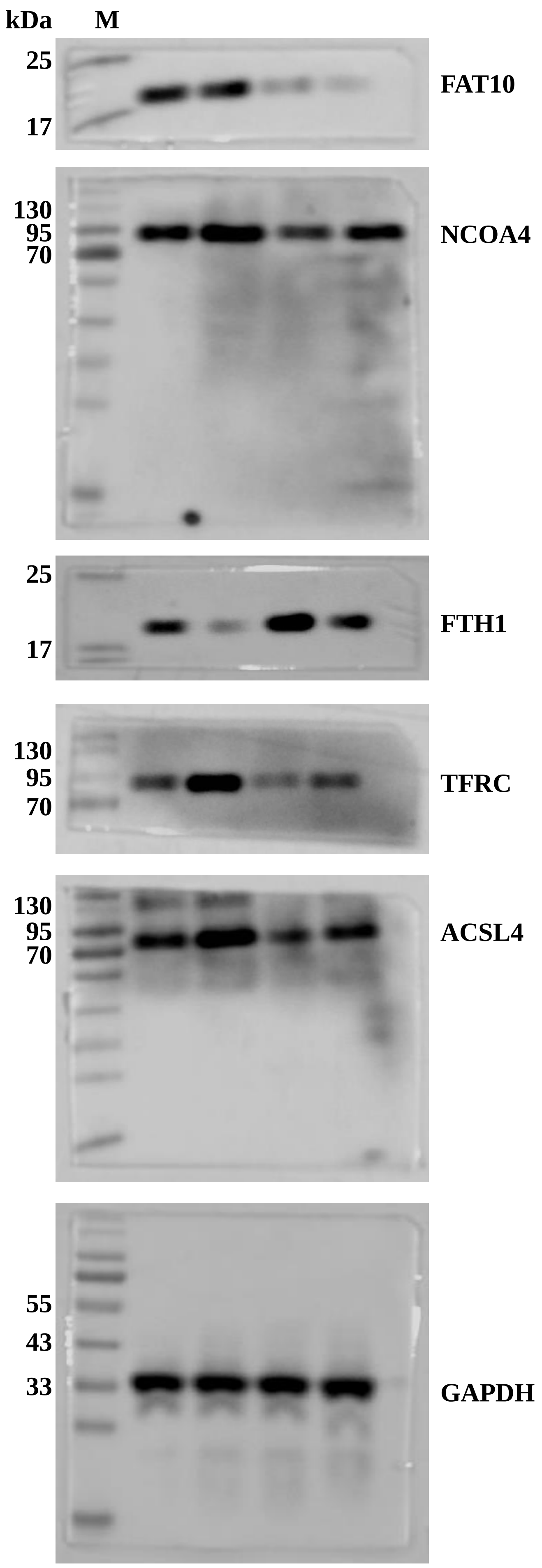
kDa
M
25
17
FAT10
130
95
70
NCOA4
25
17
FTH1
130
95
70
TFRC
130
95
70
ACSL4
55
43
33
GAPDH

Fig.3K

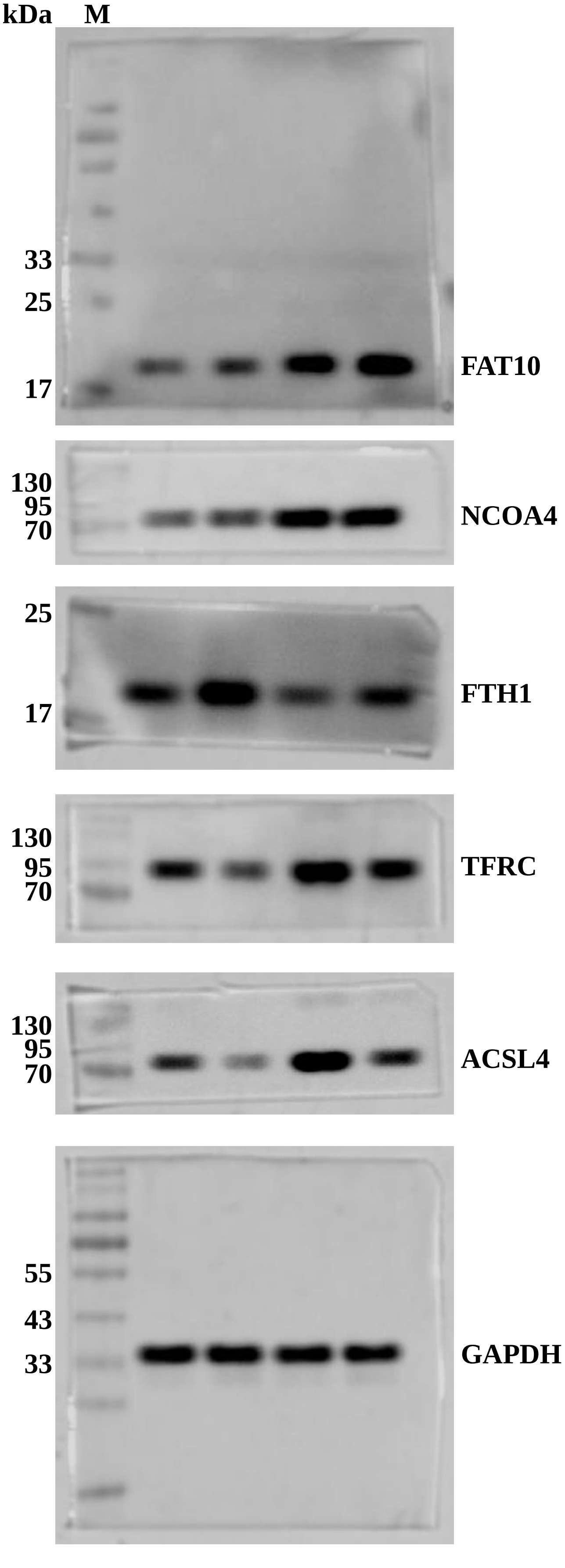
kDa
M
33
25
17
FAT10
130
95
70
NCOA4
25
17
FTH1
130
95
70
TFRC
130
95
70
ACSL4
55
43
33
GAPDH

Fig.4A

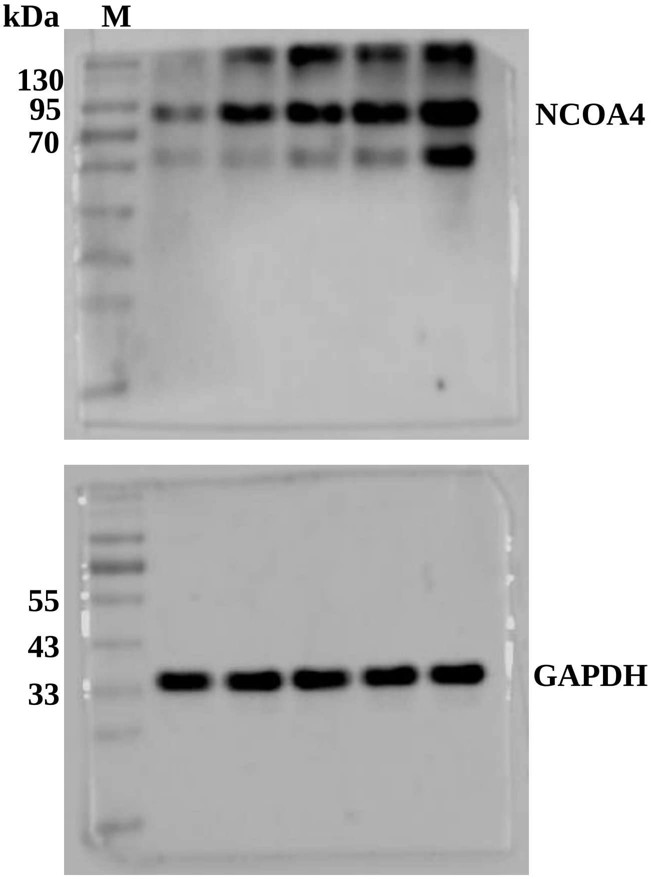

Fig.4C

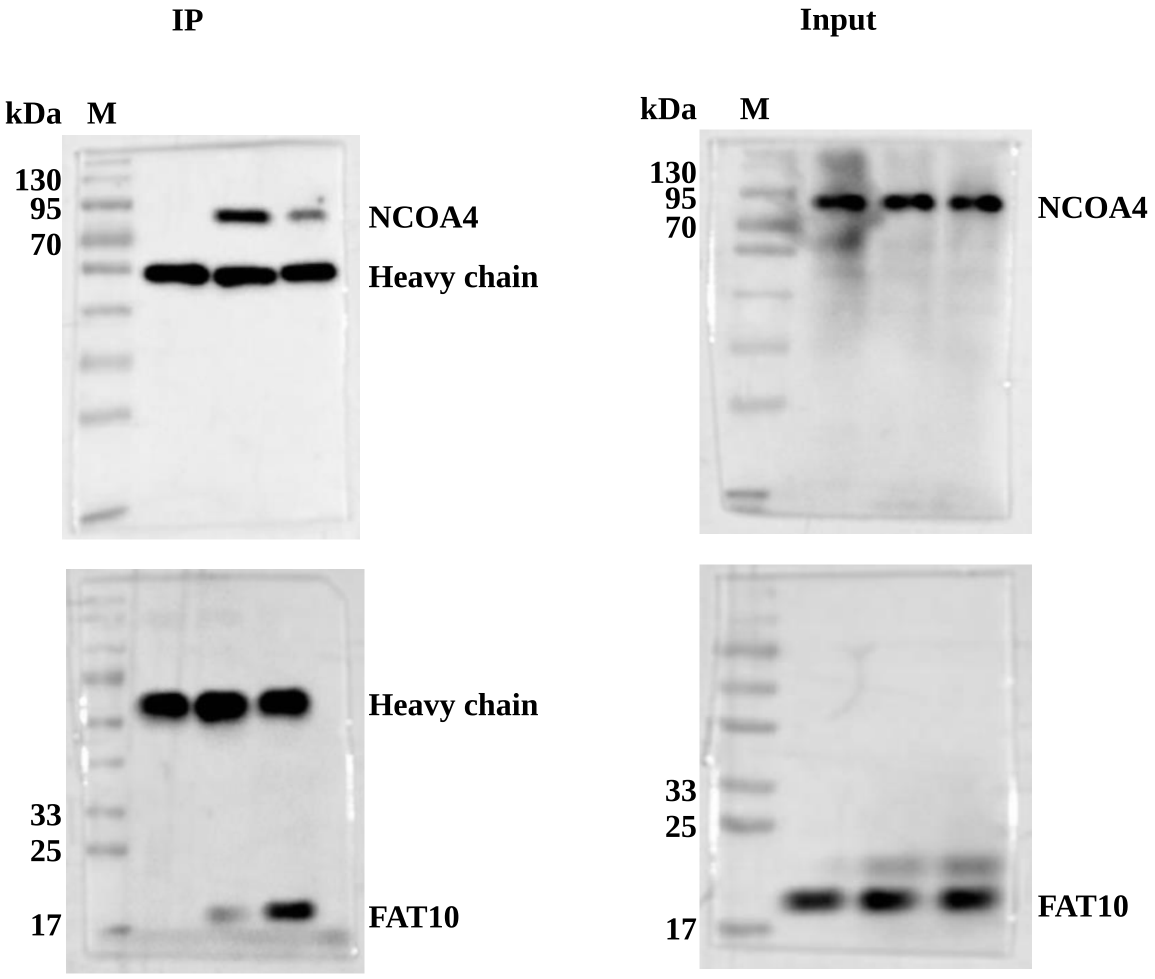
IP
Input
kDa
M
130
95
70
NCOA4
Heavy chain
Heavy chain
33
25
17
FAT10
kDa
M
130
95
70
NCOA4
33
25
17
FAT10

Fig.4D

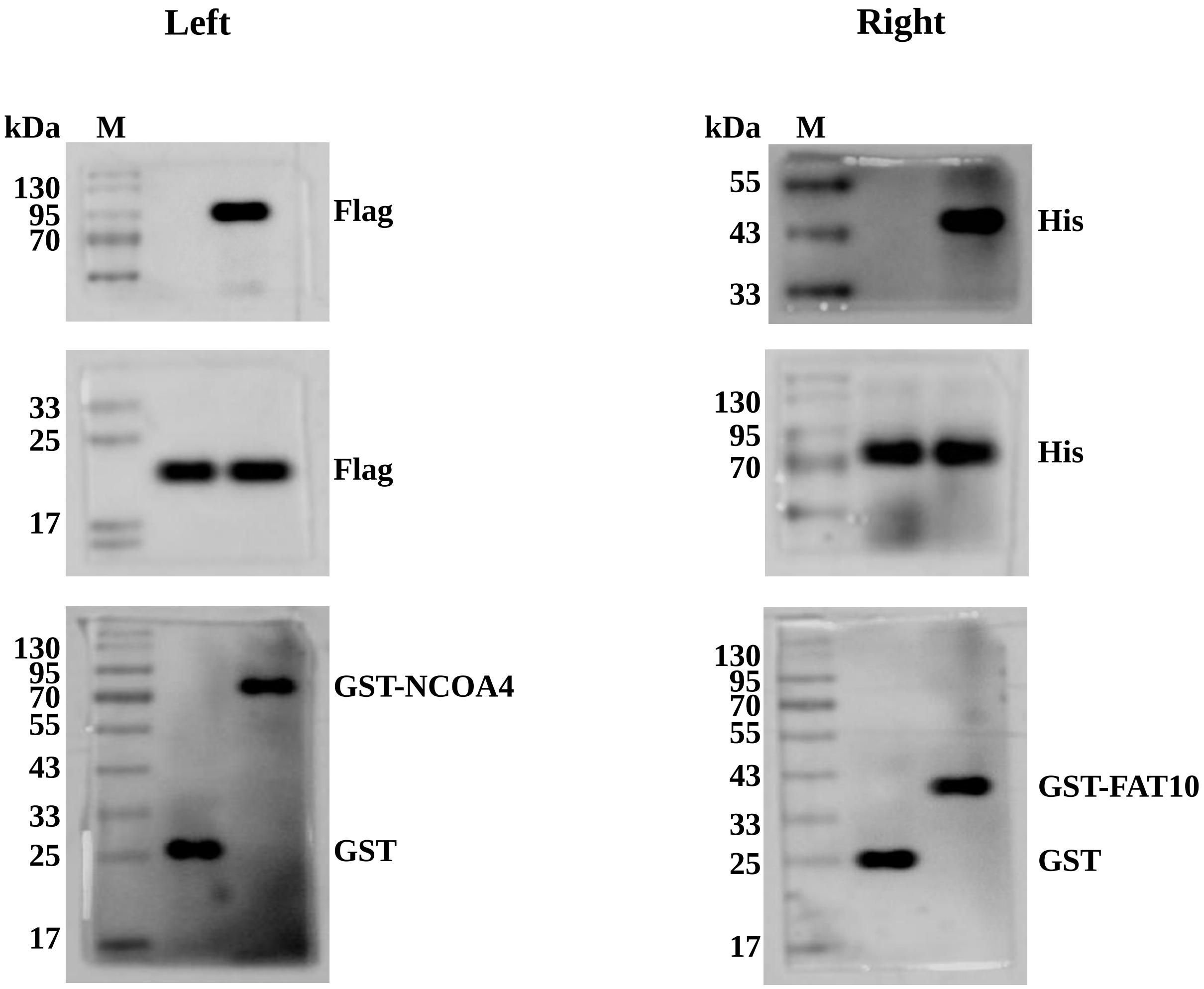

Left
Right
kDa
M
130
95
70
Flag
33
25
17
Flag
130
95
70
55
43
33
25
17
GST-NCOA4
GST
kDa
M
55
43
33
His
130
95
70
His
130
95
70
55
43
33
25
17
GST-FAT10
GST

Fig.4F

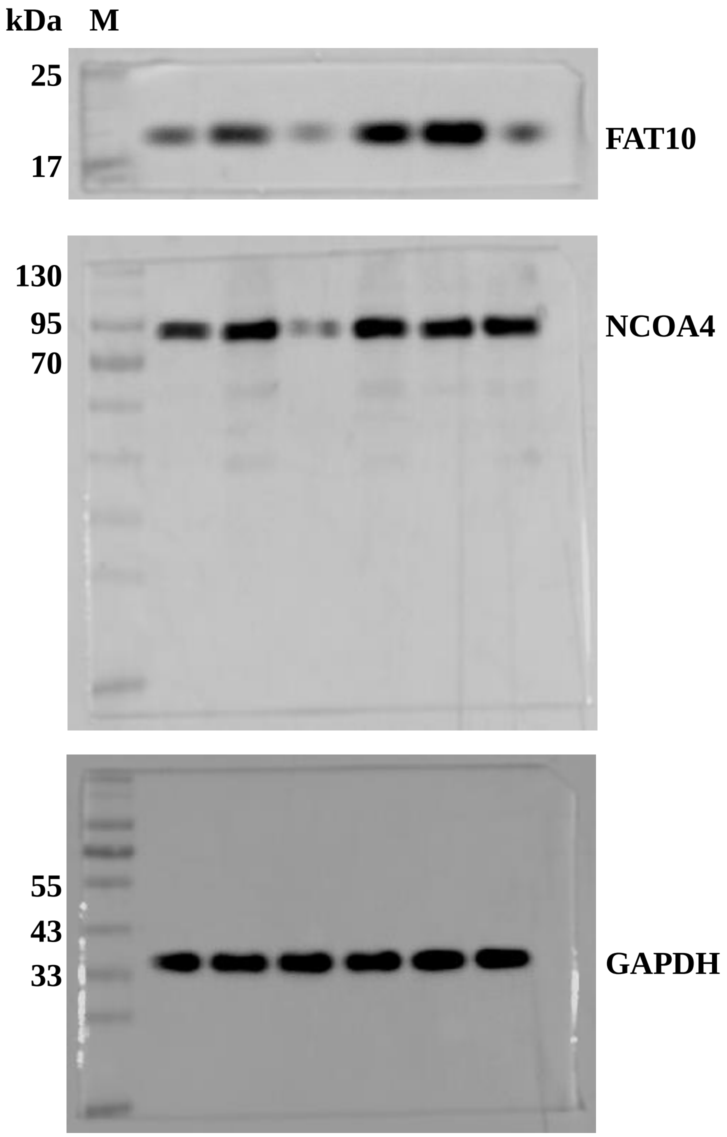

Fig.4G

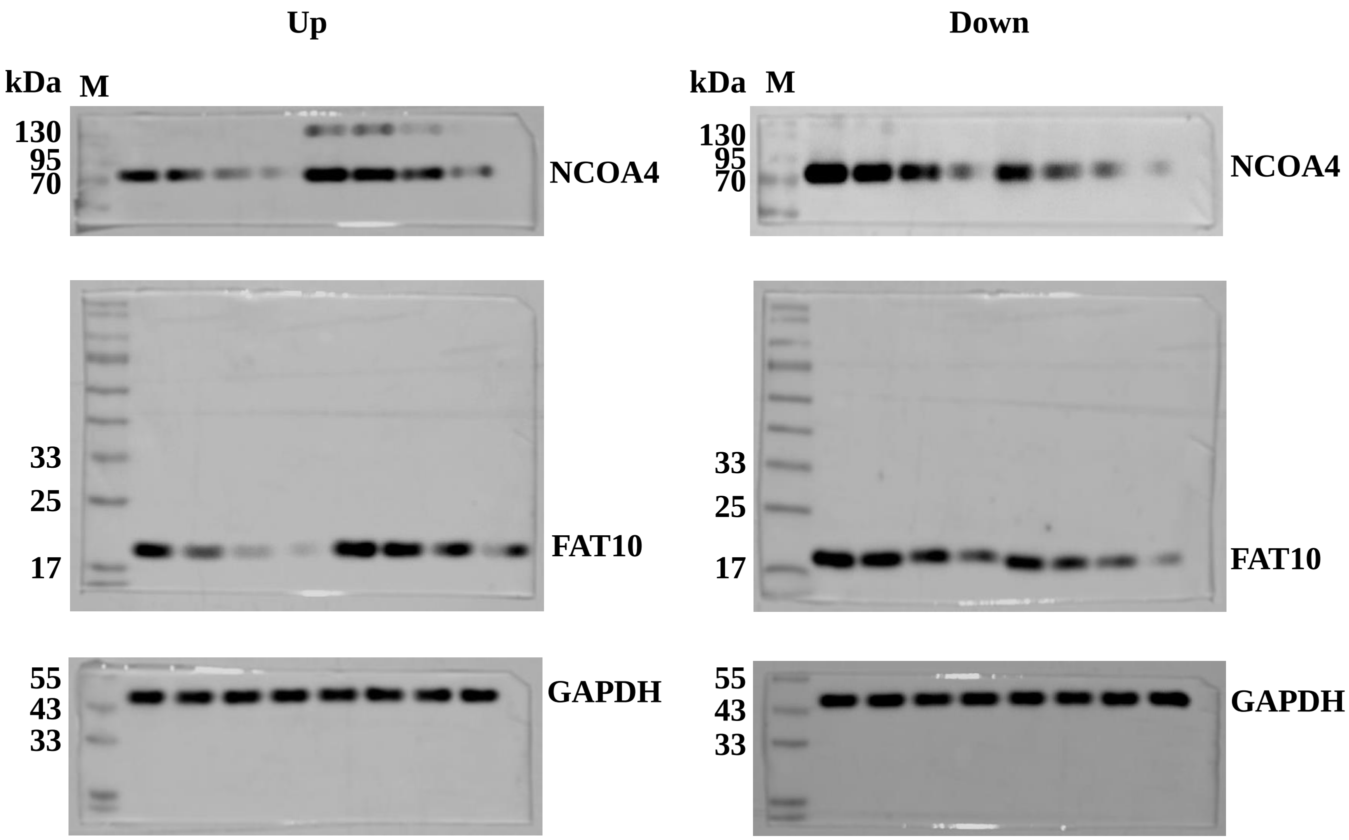

Up
Down
kDa
M
130
95
70
NCOA4
33
25
17
FAT10
55
43
33
GAPDH
kDa
M
130
95
70
NCOA4
33
25
17
FAT10
55
43
33
GAPDH

Fig.4H

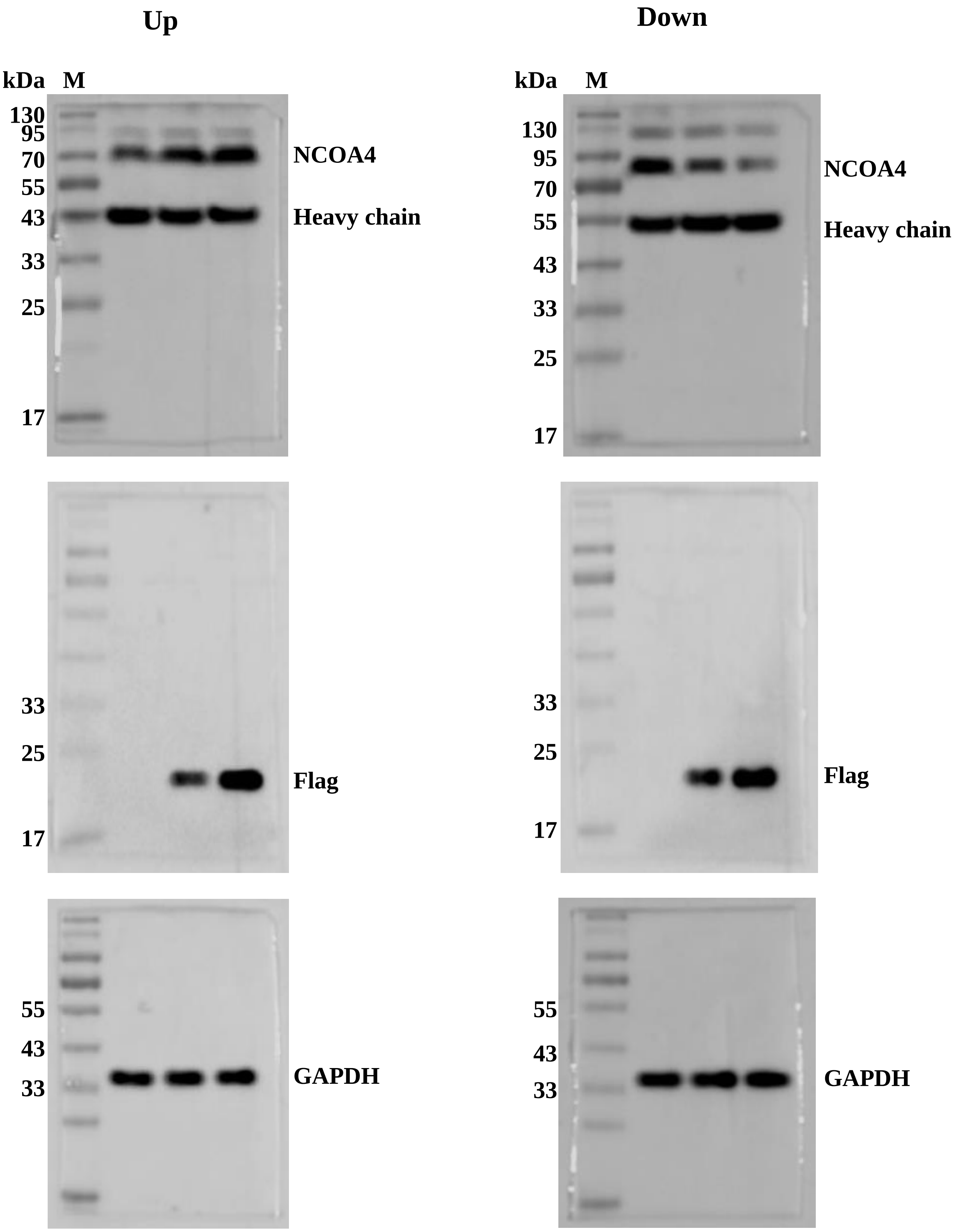
Up
Down
kDa
M
130
95
70
55
43
33
25
17
NCOA4
Heavy chain
kDa
M
130
95
70
55
43
33
25
17
NCOA4
Heavy chain
33
25
17
Flag
33
25
17
Flag
55
43
33
GAPDH
55
43
33
GAPDH

Fig.4I Left

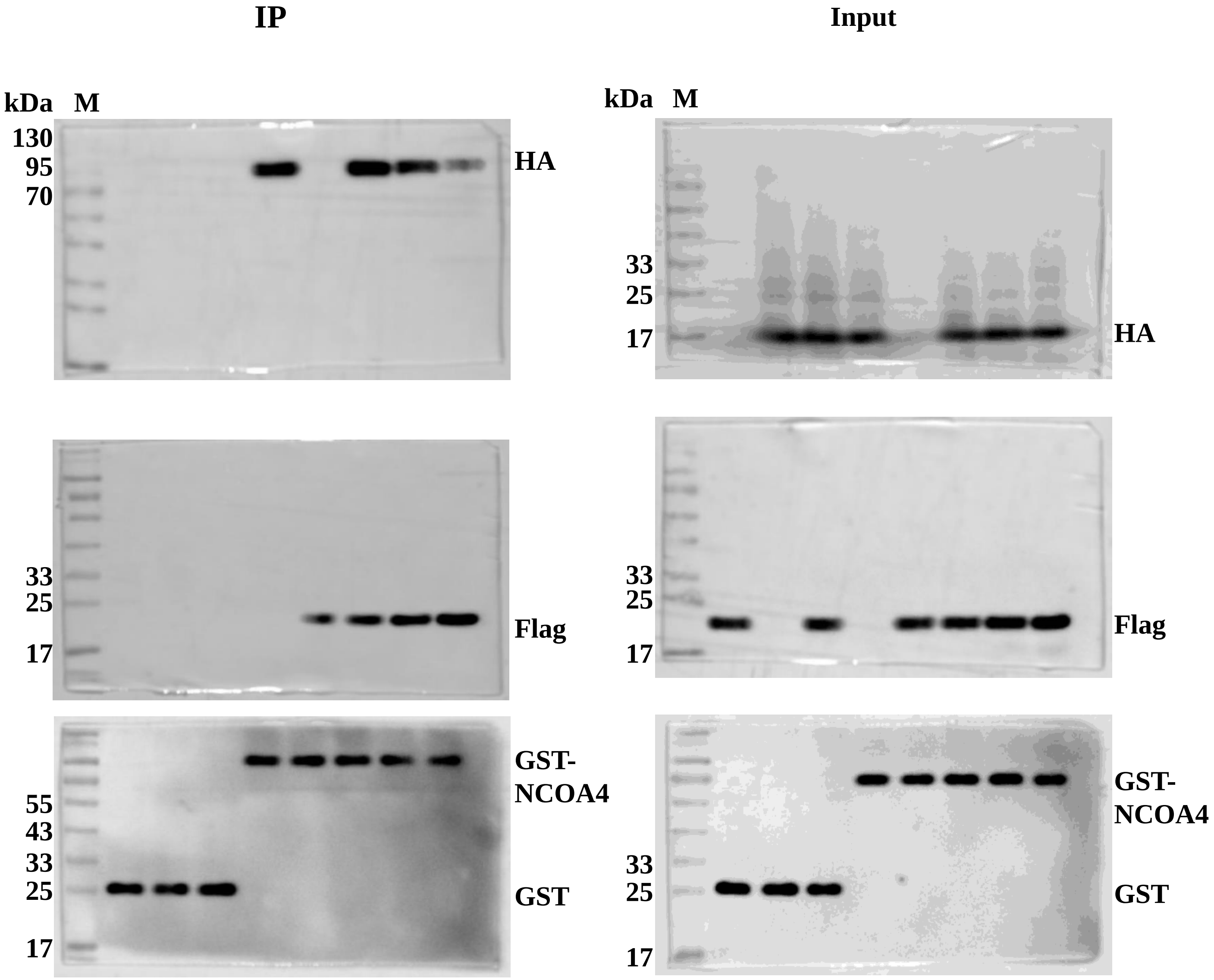

Fig.4I Right

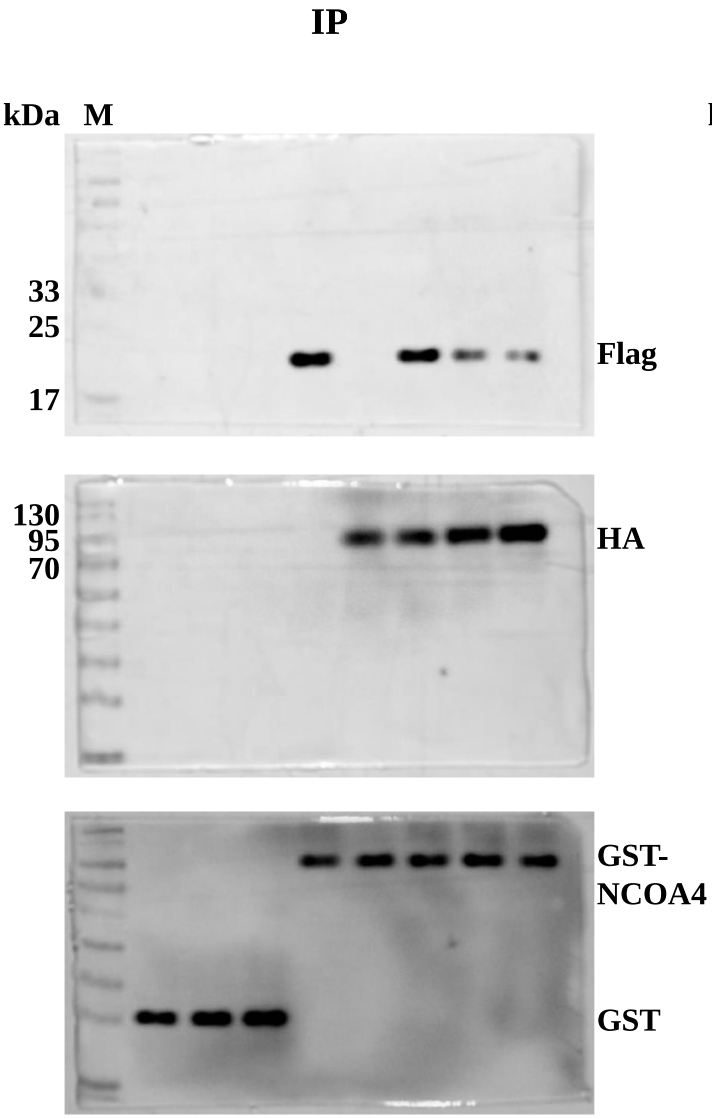
IP
kDa
M
33
25
17
Flag
130
95
70
HA
GST-
NCOA4
GST

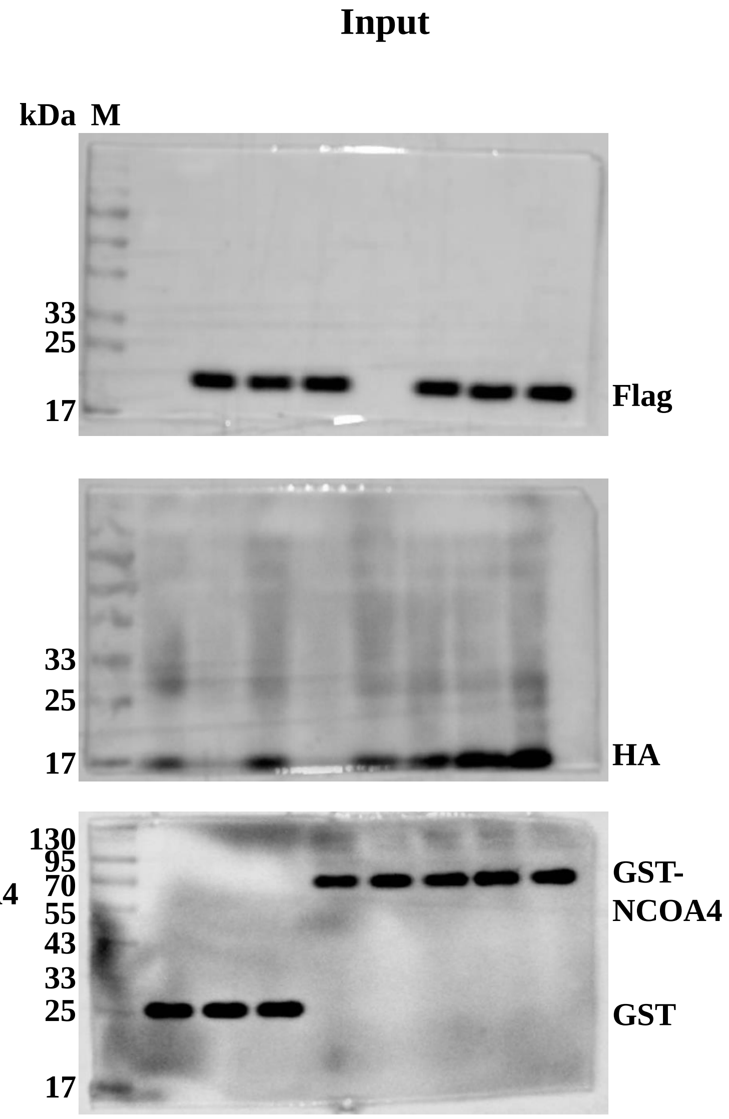
Input
kDa
M
33
25
17
Flag
33
25
17
HA
130
95
70
55
43
33
25
17
GST-
NCOA4
GST

Fig.4J

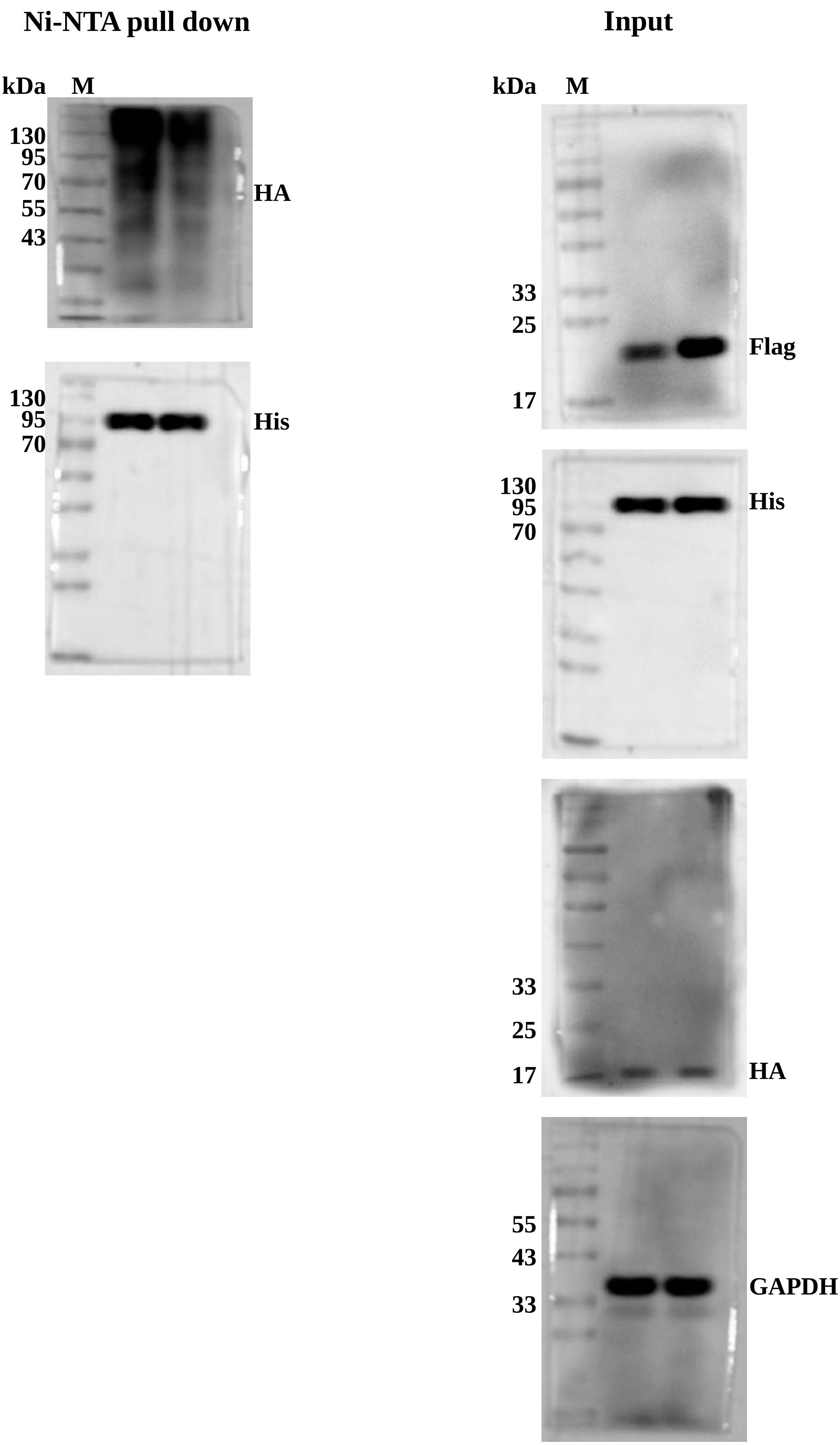
Ni-NTA pull down
Input
kDa
M
130
95
70
55
43
HA
130
95
70
His
kDa
M
33
25
17
Flag
130
95
70
His
33
25
17
HA
55
43
33
GAPDH

Fig.4K

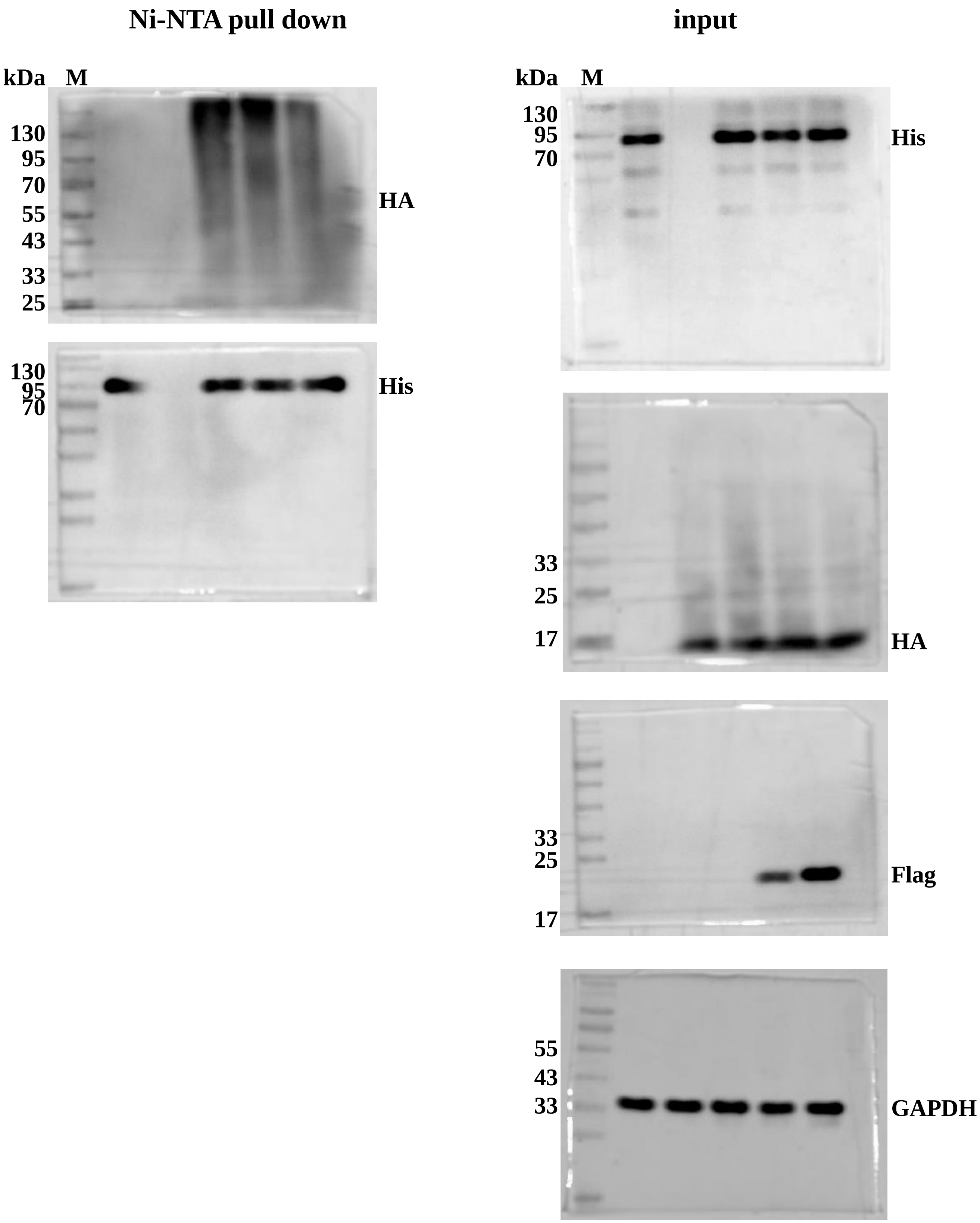
Ni-NTA pull down
input
kDa
M
130
95
70
55
43
33
25
HA
130
95
70
His
kDa
M
130
95
70
His
33
25
17
HA
33
25
17
Flag
55
43
33
GAPDH

Fig.5A Left

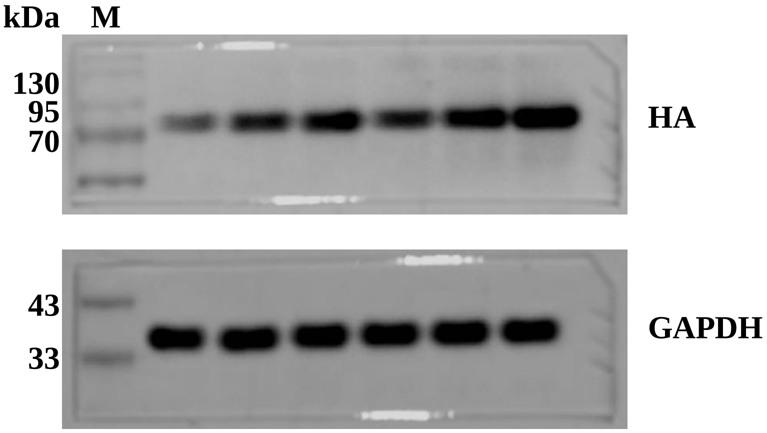

Fig.5A Right

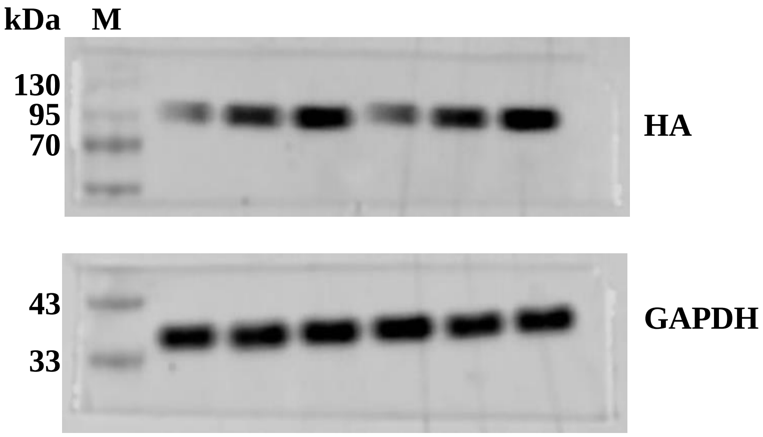

Fig.5B

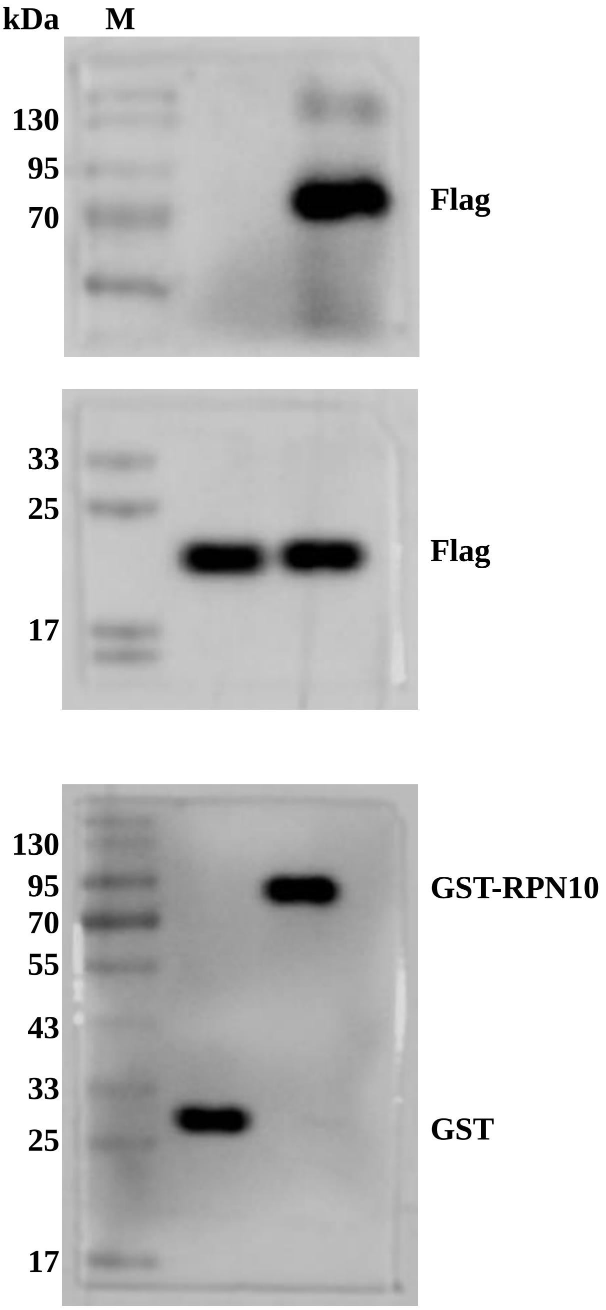

kDa
M
130
95
70
Flag
33
25
17
Flag
130
95
70
55
43
33
25
17
GST-RPN10
GST

Fig.5D Up

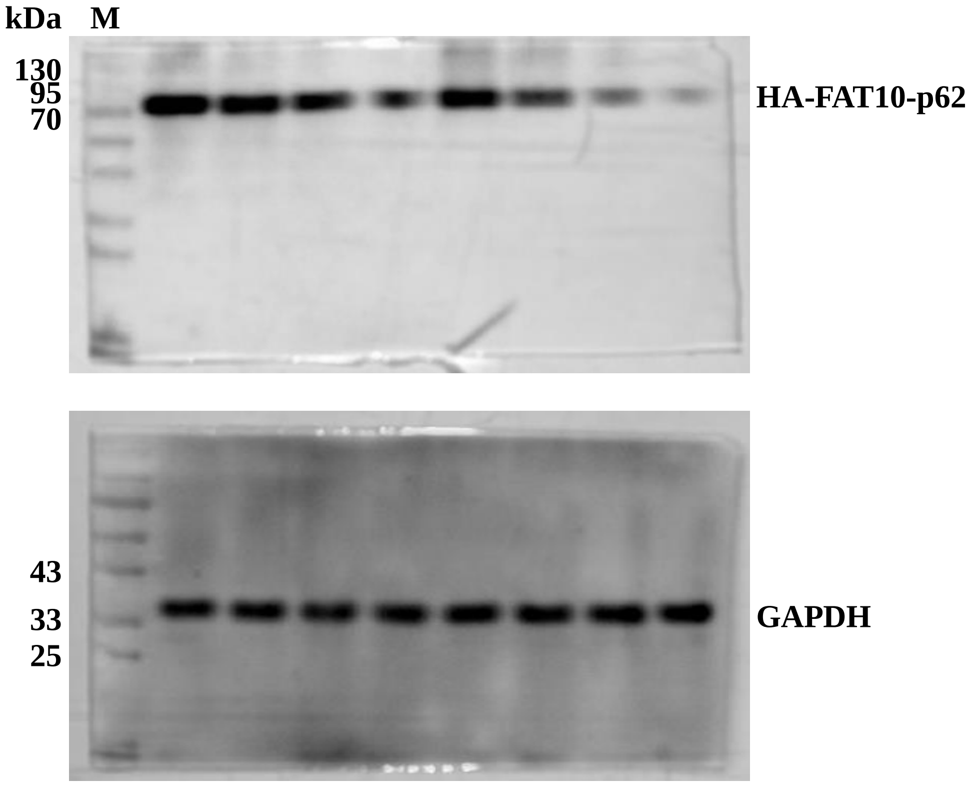

Fig.5D Down

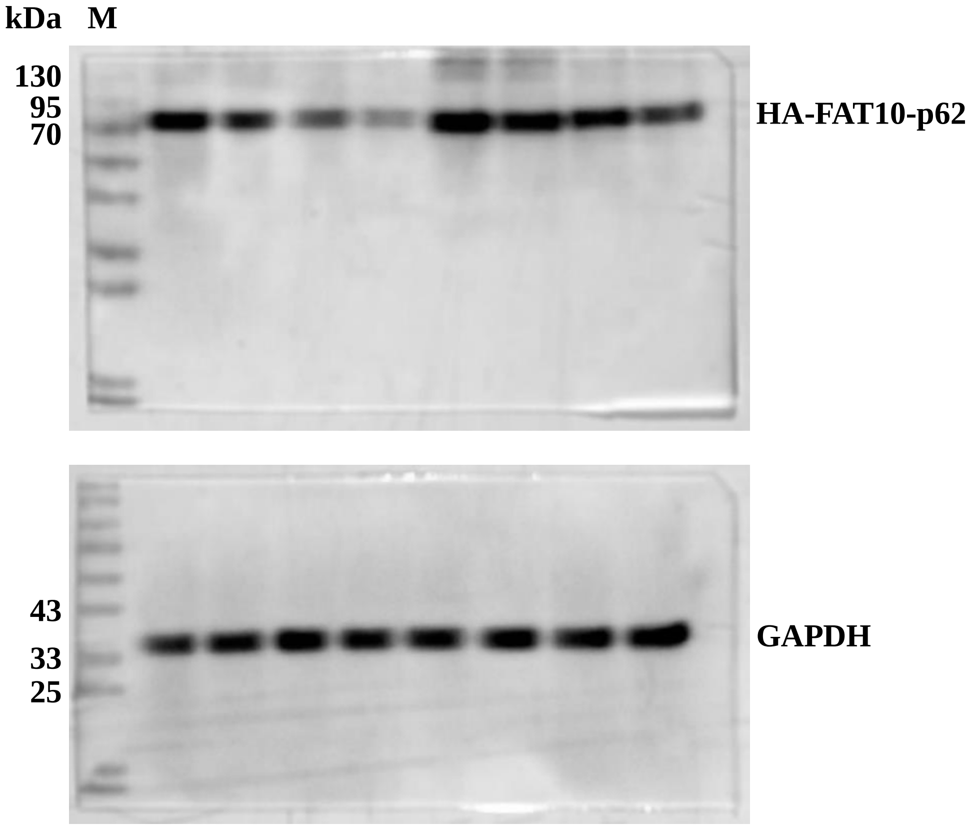

Fig.5E Up

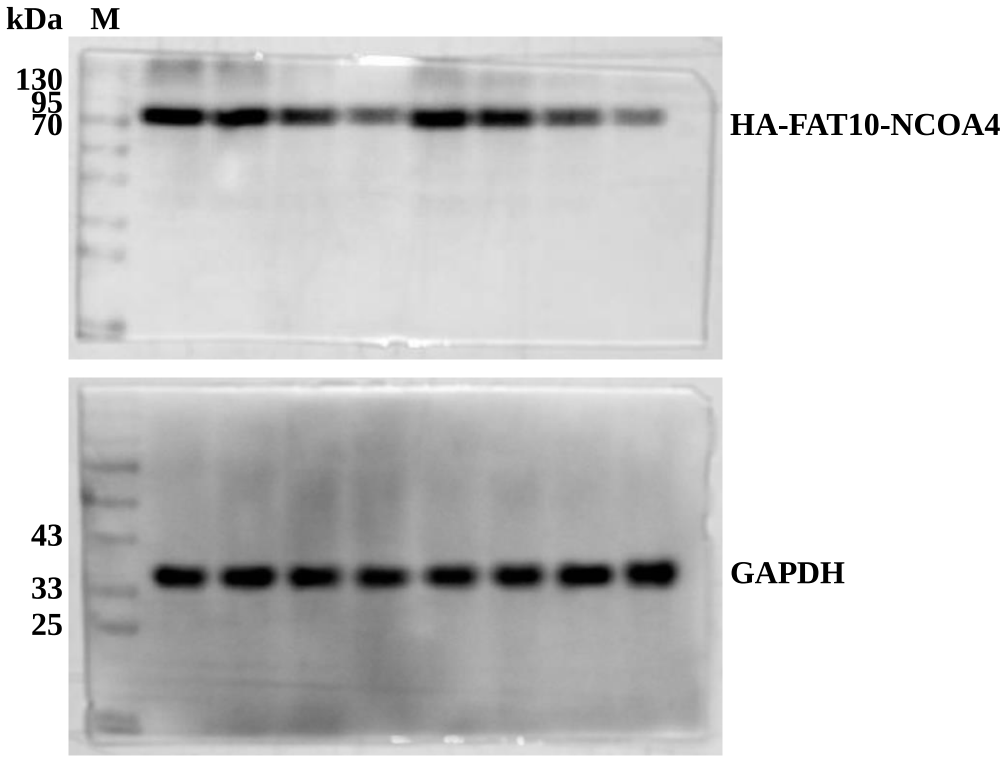

Fig.5E Down

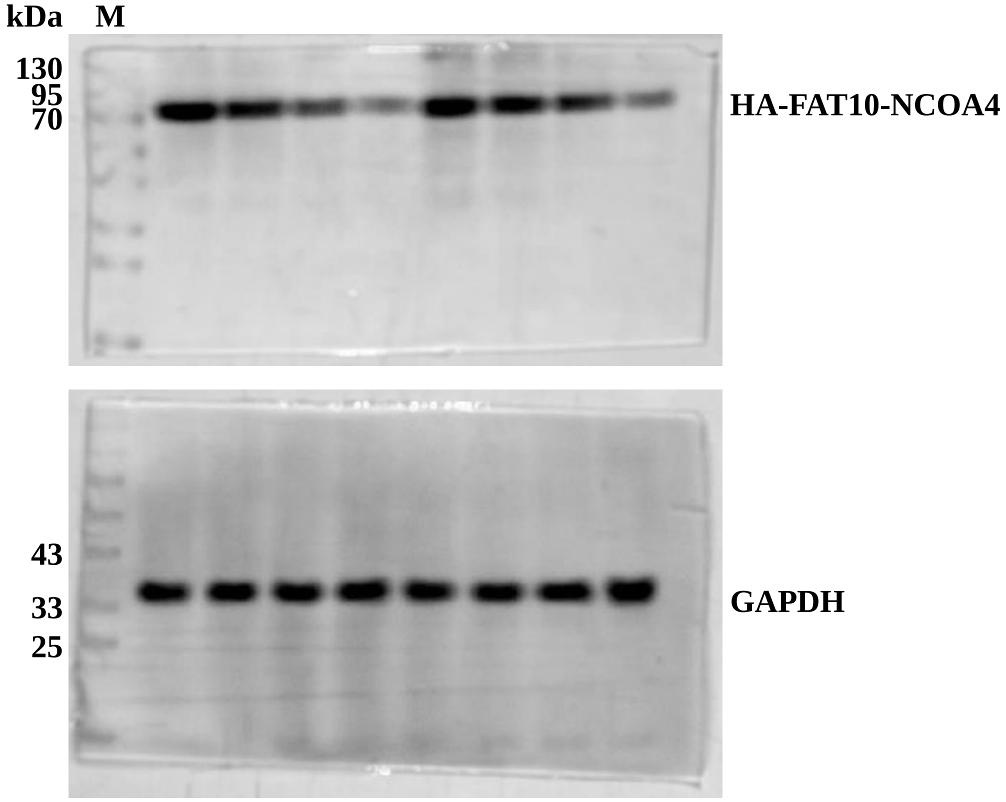

Fig.6I

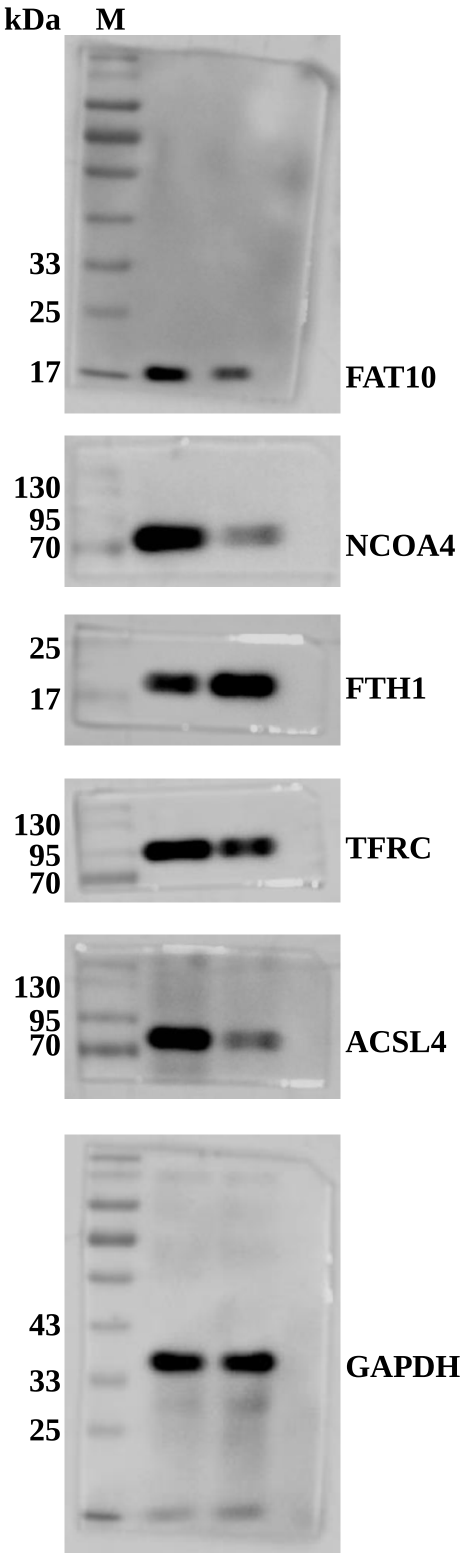
kDa
M
33
25
17
FAT10
130
95
70
NCOA4
25
17
FTH1
130
95
70
TFRC
130
95
70
ACSL4
43
33
25
GAPDH

Fig.6L

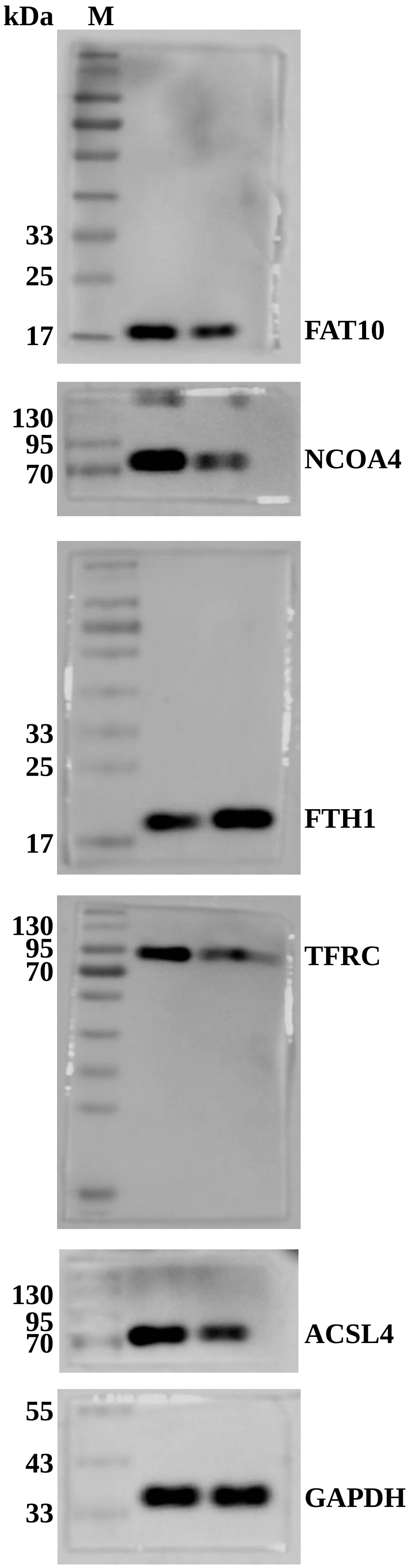
kDa
M
33
25
17
FAT10
130
95
70
NCOA4
33
25
17
FTH1
130
95
70
TFRC
130
95
70
ACSL4
55
43
33
GAPDH

Supplementary Fig. 2A

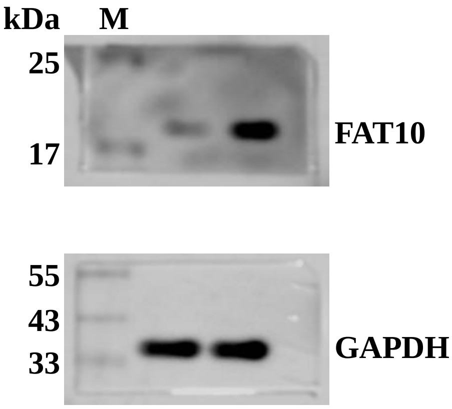

Supplementary Fig. 2H

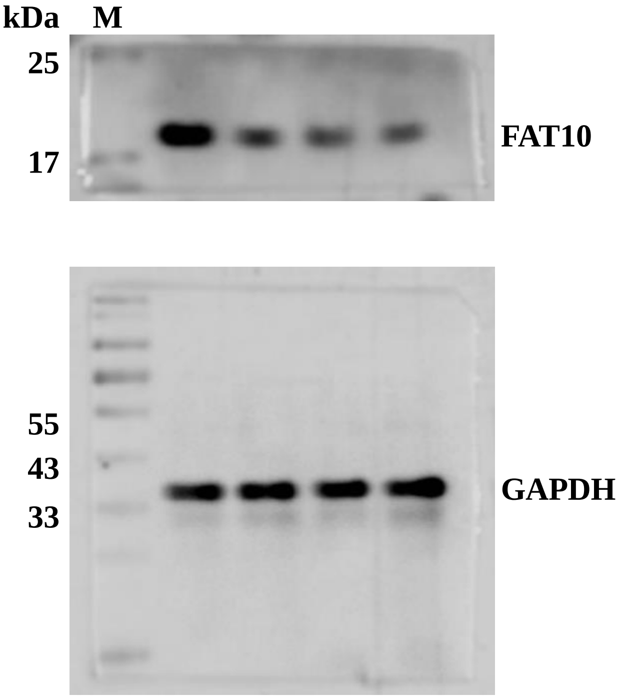

Supplementary Fig.4A

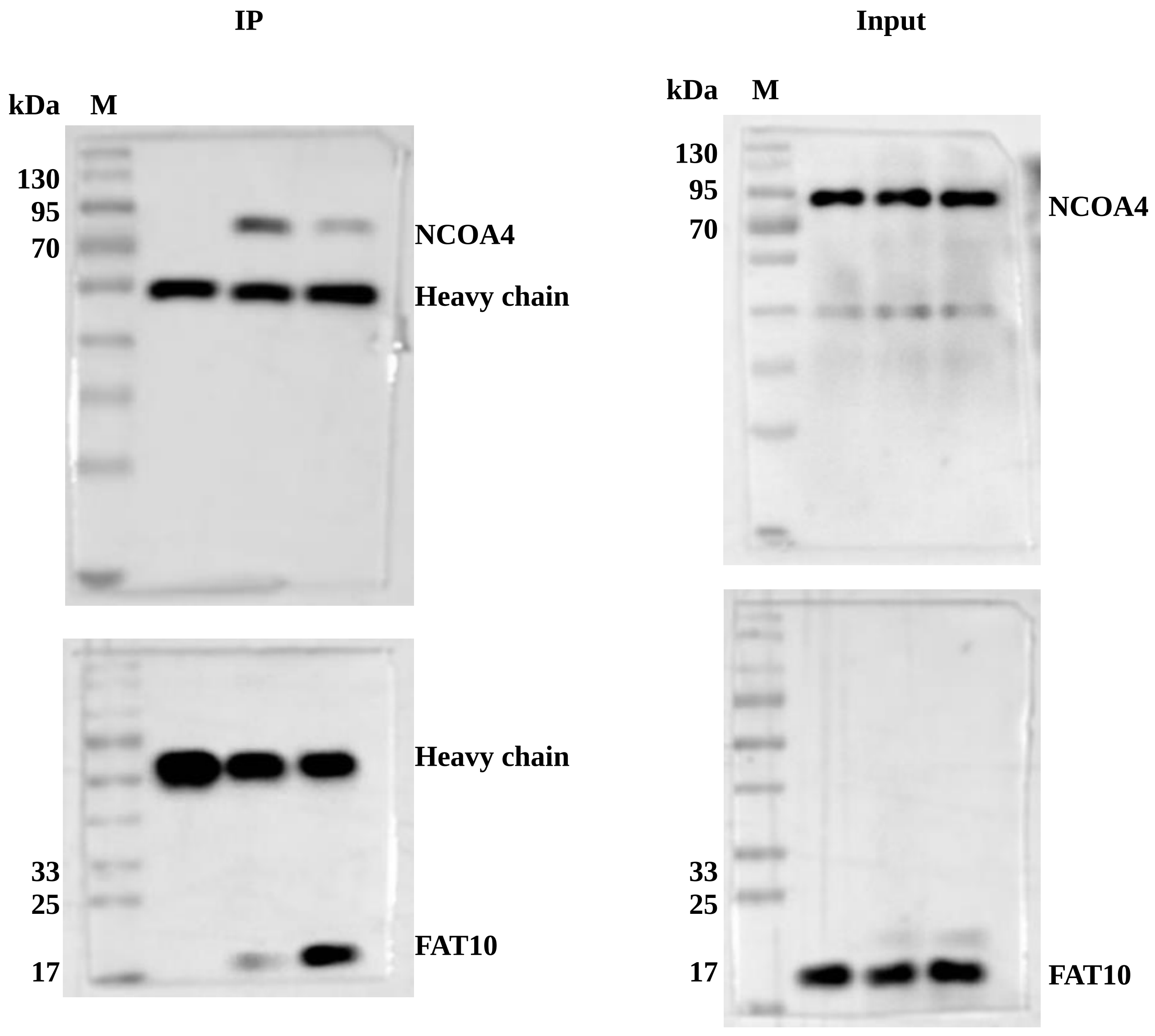

Supplementary Fig. 6A

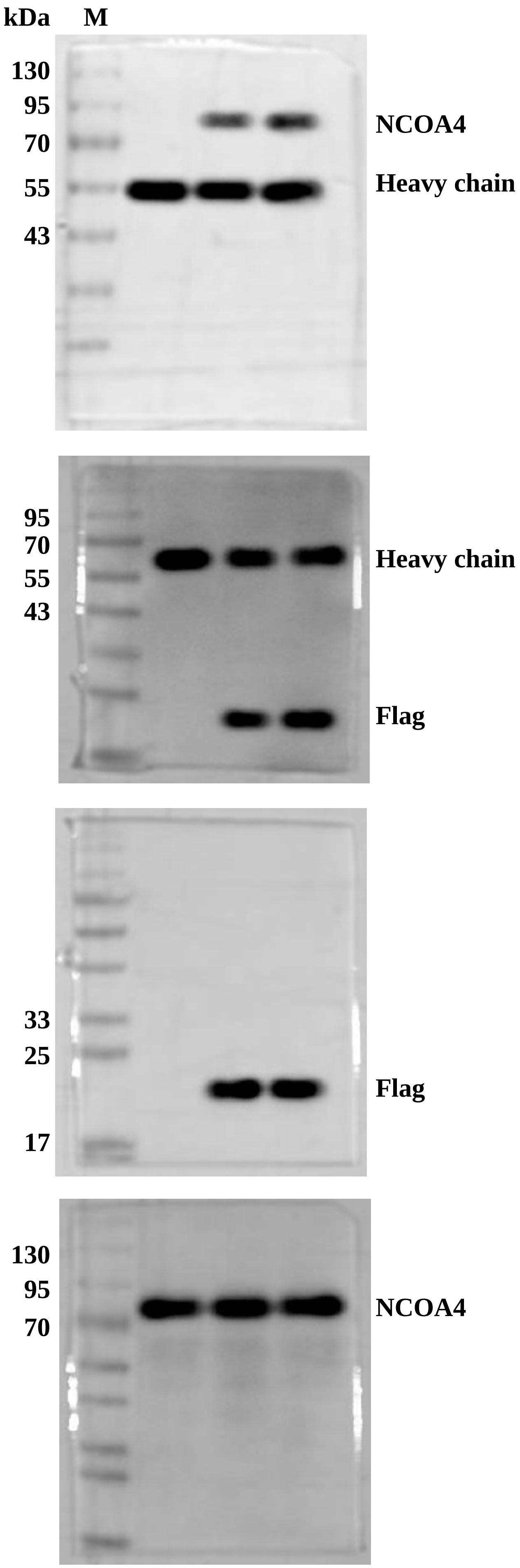

Supplementary Fig. 6B

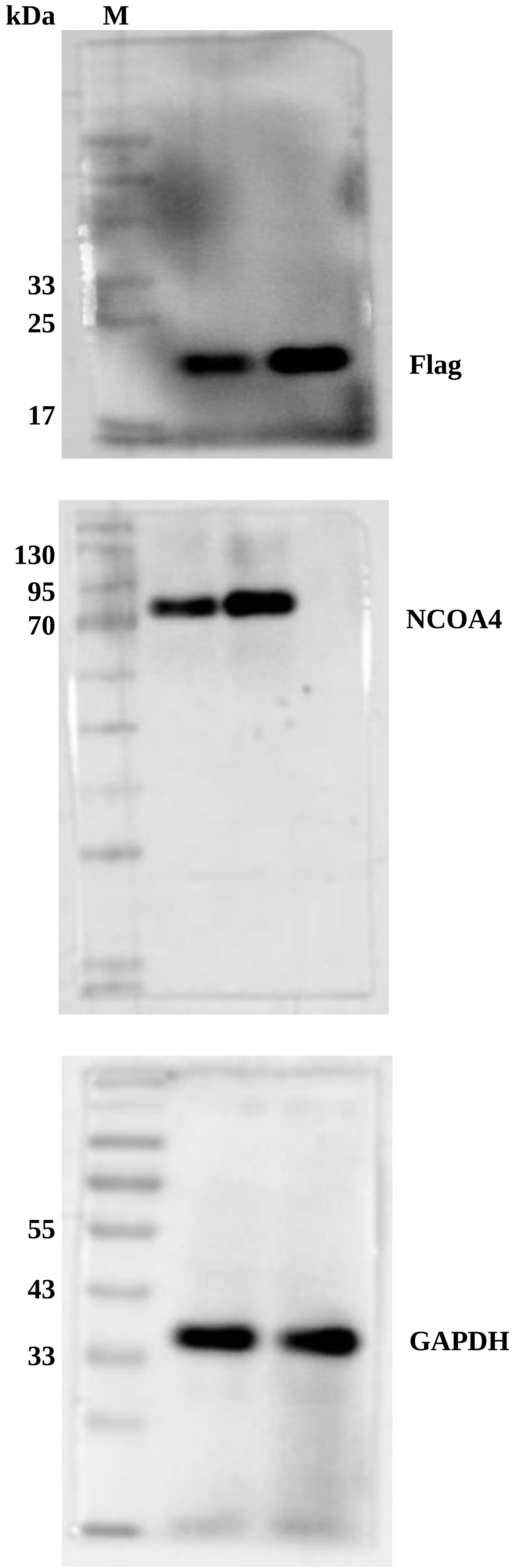

Supplementary Fig. 6D

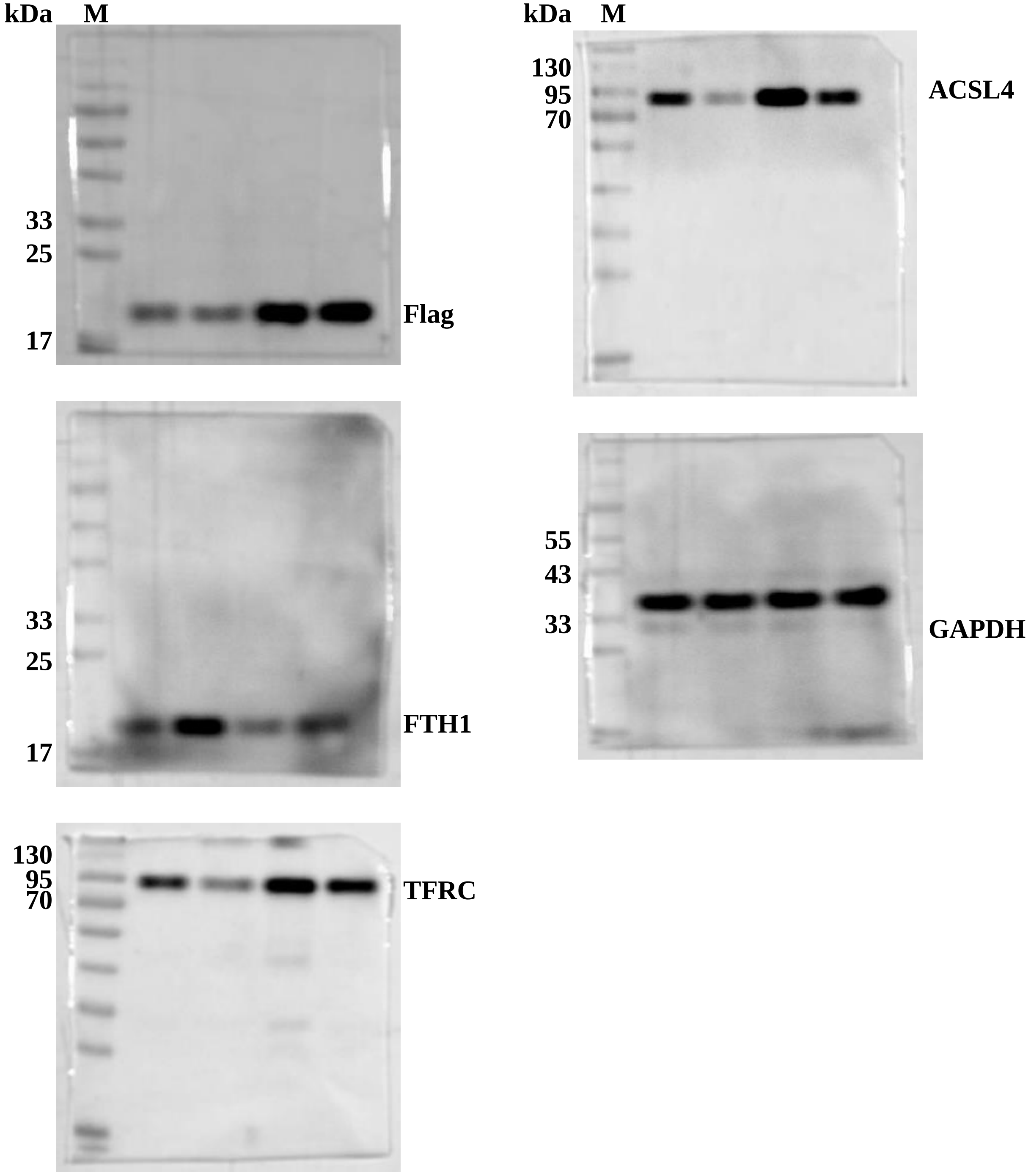

Supplementary Fig. 6G

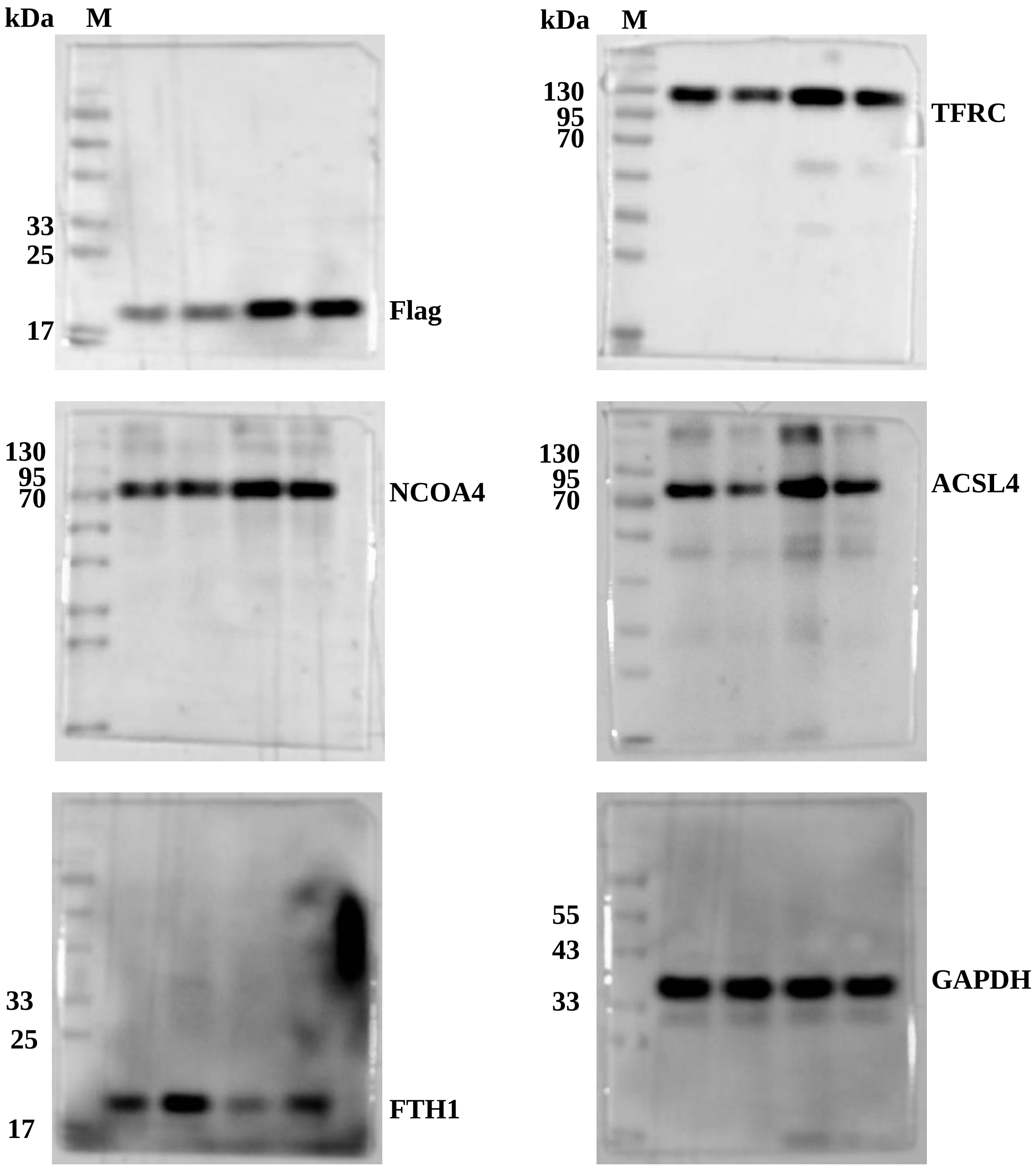

Supplement: Supplementary file 5 — Supplemental material-original figure [file 41419_2025_7715_MOESM5_ESM.pdf]
